# Hydrogen Peroxide Elicits Constriction of Skeletal Muscle Arterioles by Activating the Arachidonic Acid Pathway

**DOI:** 10.1371/journal.pone.0103858

**Published:** 2014-08-05

**Authors:** Viktória Csató, Attila Pető, Ákos Koller, István Édes, Attila Tóth, Zoltán Papp

**Affiliations:** 1 Division of Clinical Physiology, Institute of Cardiology, University of Debrecen, Debrecen, Hungary; 2 Research Centre for Molecular Medicine, University of Debrecen, Debrecen, Hungary; 3 Department of Pathophysiology and Gerontology, University of Pécs, Pécs, Hungary; 4 Department of Pathophysiology, Semmelweis University, Budapest, Hungary; 5 Department of Physiology, New York Medical College, Valhalla, New York, United States of America; University of Hull, United Kingdom

## Abstract

**Aims:**

The molecular mechanisms of the vasoconstrictor responses evoked by hydrogen peroxide (H_2_O_2_) have not been clearly elucidated in skeletal muscle arterioles.

**Methods and Results:**

Changes in diameter of isolated, cannulated and pressurized gracilis muscle arterioles (GAs) of Wistar-Kyoto rats were determined under various test conditions. H_2_O_2_ (10–100 µM) evoked concentration-dependent constrictions in the GAs, which were inhibited by endothelium removal, or by antagonists of phospholipase A (PLA; 100 µM 7,7-dimethyl-(5Z,8Z)-eicosadienoic acid), protein kinase C (PKC; 10 µM chelerythrine), phospholipase C (PLC; 10 µM U-73122), or Src family tyrosine kinase (Src kinase; 1 µM Src Inhibitor-1). Antagonists of thromboxane A2 (TXA2; 1 µM SQ-29548) or the non-specific cyclooxygenase (COX) inhibitor indomethacin (10 µM) converted constrictions to dilations. The COX-1 inhibitor (SC-560, 1 µM) demonstrated a greater reduction in constriction and conversion to dilation than that of COX-2 (celecoxib, 3 µM). H_2_O_2_ did not elicit significant changes in arteriolar Ca^2+^ levels measured with Fura-2.

**Conclusions:**

These data suggest that H_2_O_2_ activates the endothelial Src kinase/PLC/PKC/PLA pathway, ultimately leading to the synthesis and release of TXA2 by COX-1, thereby increasing the Ca^2+^ sensitivity of the vascular smooth muscle cells and eliciting constriction in rat skeletal muscle arterioles.

## Introduction

Among its many important roles, H_2_O_2_ is involved as a signalling molecule in the physiological regulation of the vascular diameter. Moreover, H_2_O_2_ can contribute to the development of a vascular dysfunction in hypertension [1], [2], diabetes [3], [4] and atherosclerosis [5]. Nevertheless, the vascular signalling pathways mobilized by H_2_O_2_ have not been fully elucidated.

H_2_O_2_ can be produced by endothelial cells, smooth muscle cells and fibroblasts [6], [7], under both physiological and pathological conditions. Moreover, significant amounts of H_2_O_2_ are released by activated leukocytes under inflammatory conditions [8]. Numerous enzyme systems, including NAD(P)H oxidase [9], [10], the mitochondrial respiratory chain, xanthine oxidase, uncoupled endothelial nitric oxide (NO) synthase, cytochrome P-450 enzymes, lipoxygenase and the cyclooxigenases [11]–[16], can generate the superoxide anion (O_2_^−^), which is then reduced to H_2_O_2_. There can be a great variation in the extracellular concentration of H_2_O_2_, but it can probably reach 0.3 mM [8], [17], [18].

H_2_O_2_ has been shown to act as an endothelium-derived hyperpolarizing factor (EDHF) in several vascular beds, including porcine coronary arterioles, mouse mesenteric arterioles, rat ophthalmic arteries and rat coronary arterioles [19]–[23]. It has been proposed that, as an EDHF, H_2_O_2_ contributes to the development of functional hyperaemia in human coronary and mesenteric arterioles [24], [25]. Another important role ascribed to H_2_O_2_ is the mediation of flow-induced dilation in human coronary arterioles [26], [27] and as such it may provide an important back-up dilator mechanism when levels of NO are reduced [28]. In contrast, H_2_O_2_ results in vasoconstriction in the rat aorta [29], [30] and renal artery [31], the rabbit pulmonary artery [32] and the canine basilar arterioles [33], [34]. Surprisingly, H_2_O_2_ has also been shown to exert a concentration-dependent biphasic effect (*i.e.* vasoconstriction followed by vasodilation) in the skeletal muscle and mesenteric arterioles of the rat [8], [35].

Previous studies have revealed certain fragments of the signalling cascades responsible for the H_2_O_2_-evoked vascular constrictions and dilations in various species and preparations. Thus, H_2_O_2_ has been shown to evoke vasodilation by activation of arachidonic acid (AA) metabolism and subsequent cyclic adenosine monophosphate production in canine cerebral arteries [36]. Moreover, H_2_O_2_ has been claimed to activate the NO/cyclic guanosine monophosphate pathway in rat skeletal muscle arterioles and in the rabbit aorta [8], [37]. Increased cGMP levels lead to the release of endothelium-derived dilator prostaglandins in porcine coronary arterioles [38], whereas the endothelium-independent relaxation to H_2_O_2_ in porcine coronary arterioles involves the activation of K^+^ channels [39]–[42]. Similarly to the above vasodilatory mechanisms, it is hypothesized that in different vessel types/species several distinct signalling molecules can contribute to the H_2_O_2_-evoked constrictor effects, including COX products [8], [29], [30], [43], tyrosine kinases [29], [34] and mitogen-activated protein kinase [34], [44], [45]. Moreover, these pathways may mobilize intracellular Ca^2+^-dependent mechanisms in vascular smooth muscle cells to evoke vasoconstriction [29], [34], although the activation of Ca^2+^-independent alternative pathways cannot be excluded [46].

Taken together, H_2_O_2_ apparently activates complex second messenger systems in the vascular endothelium and smooth muscle cells to evoke vasoconstriction, although the exact signalling pathway and its ability to change intracellular Ca^2+^ concentrations are not well understood. In the present study, therefore, we investigated the acute effects of H_2_O_2_ on the diameter of arterioles isolated from rat skeletal muscle and rat coronaries, the signal transduction pathway initiating H_2_O_2_-evoked vasoconstriction, and the changes in vascular smooth muscle intracellular Ca^2+^ concentrations induced by H_2_O_2_.

## Methods

### Ethical statement

All procedures employed in this work conformed to strictly Directive 2010/63/EU of the European Parliament and were approved by the Ethical Committee of the University of Debrecen.

### Animals, anaesthesia and tissue dissection

Experiments were performed on male Wistar rats (approximately 10 weeks of age, weighing 250–350 g, obtained from Toxi-Coop Toxicological Research Centre, Dunakeszi, Hungary). The animals were fed a standard chow and drank tap water *ad libitum*. For the study, animals were anaesthetized with an intraperitoneal injection of sodium pentobarbital (150 mg/kg). All efforts were made to minimize the suffering of the animals. The gracilis muscle and the heart were removed and placed in silicone-coated petri dishes containing cold (0–4°C) Krebs solution (in mM: 110 NaCl, 5.0 KCl, 2.5 CaCl_2_, 1.0 MgSO_4_, 1.0 KH_2_PO_4_, 5.0 glucose and 24.0 NaHCO_3_) equilibrated with a gaseous mixture of 5% CO_2_, 10% O_2_ and 85% N_2_ at pH 7.4.

### Materials and drugs

The TXA2 agonist (U46619) was obtained from Calbiochem (Billerica, MA, USA), and the TXA2 inhibitor (SQ-29548) from BioMarker Kft. (Gödöllő, Hungary). All other chemicals were from Sigma-Aldrich (St. Louis, MO, USA) and were kept under the conditions prescribed by the manufacturer. All reported concentrations are the final concentrations in the organ chamber.

### Isolation of arterioles and measurement of vascular diameter

Arterioles were isolated and cannulated as described previously [47]. Briefly, gracilis muscle arterioles and the second branch of the septal coronary artery (both ∼1.5 mm long) running intramuscularly were isolated through the use of microsurgical instruments and an operating microscope and transferred into an organ chamber containing two glass micropipettes filled with Krebs solution. The arterioles were cannulated at both ends and the micropipettes were connected via silicone tubing to a pressure servo control system (Living Systems Instrumentation, St. Albans, VT, USA) to set the intraluminal pressure at 80 mmHg. The temperature was maintained at 37°C by a temperature controller. Changes in internal arteriolar diameter were recorded continuously with a video microscope system (Topica CCD camera).

### Experimental protocols

In response to the intraluminal pressure of 80 mmHg the isolated arterioles spontaneously developed a substantial myogenic tone without the use of any exogenous constrictor agents (a decrease from an initial diameter of 205±5 µm to 149±5 µm (n = 99 arterioles from 82 different animals) and from 170±14 µm to 107±7 µm (n = 17 arterioles from 17 different animals) in the skeletal and coronary arterioles of the rat, respectively).

Cumulative concentrations of acetylcholine (1 nM–10 µM) were used to test the vasomotor function of the endothelium. The smooth muscle function was tested with norepinephrine (skeletal muscle artery) or serotonin (coronary artery, 1 nM–10 µM). H_2_O_2_ solutions were prepared immediately before the experiments and were stored on ice. In the first series of experiments, cumulative concentrations of H_2_O_2_ (1 µM–10 mM) were added to the skeletal muscle arterioles (n = 6 arterioles from 6 different animals) or coronary arterioles (n = 7 arterioles from 7 different animals) and the responses to the H_2_O_2_ were determined and diameters were recorded 60 s after the application of each H_2_O_2_ concentration. During measurements, the changes in the pH of the chamber containing H_2_O_2_ were also checked. The pH of the control solutions did not vary significantly with the final concentration of H_2_O_2_ (pH 7.52±0.03 in the absence of H_2_O_2_, pH 7.58±0.03 in the presence of 10 mM H_2_O_2_, n = 3). To study the kinetics of diameter changes, various concentrations of H_2_O_2_ (10, 30, 100 and 300 µM) were used (600 s treatment duration, diameter measured every 10 s, n = 3–5 arterioles from 11 different animals at each concentration). In some groups of experiments, the endothelium was removed by air perfusion of the arterioles (denudation, n = 6 arterioles from 6 different animals). Successful endothelium denudation was verified by the loss of dilation in response to acetylcholine (96±5% dilation before and 0.3±0.2% after endothelium removal), whereas a maintained smooth muscle function was confirmed through the use of norepinephrine (62±6% constriction before and 55±6% after endothelium removal).

The effects of H_2_O_2_ on the diameter of the arterioles were also measured in the presence (15–30-min preincubation) of a PKC inhibitor (chelerythrine, 10 µM, n = 5 arterioles from 5 different animals), a PLC inhibitor (U73122, 10 µM, n = 4 arterioles from 4 different animals), a PLA inhibitor (7,7-dimethyl-(5Z,8Z)-eicosadienoic acid, 100 µM, n = 5 arterioles from 5 different animals), a Src kinase inhibitor (Src inhibitor-1, 5 µM, n = 5 arterioles from 5 different animals), a COX-1 and COX-2 inhibitor (indomethacin, 10 µM, n = 5 arterioles from 4 different animals), a COX-1-selective inhibitor (SC-560, 1 µM, n = 5 arterioles from 3 different animals), a COX-2- selective inhibitor (celecoxib, 3 µM, n = 4 arterioles from 4 different animals), another COX-2-selective inhibitor (NS-398, 10 µM, n = 3 arterioles from 3 different animals) and a TXA2 receptor inhibitor (SQ-29548, 1 µM, n = 10 arterioles from 10 different animals). The inhibitors were dissolved in dimethyl sulphoxide (DMSO), ethanol or in water. The maximum concentration of non-aqueous solvent (DMSO or ethanol) in the organ chamber was 0.1%. The solvents alone had no vascular effects.

At the end of the experiments, the maximum (passive) arteriolar diameter was determined in the absence of extracellular Ca^2+^ at an intraluminal pressure of 80 mmHg.

### Parallel measurement of vascular diameter and intracellular Ca^2+^ concentrations

Simultaneous measurements of intracellular Ca^2+^ and arteriolar diameter were performed as described previously [48], [49]. Briefly, isolated and cannulated arterioles (n = 9 arterioles from 6 animals) were incubated for 60 min in the presence of physiological buffer solution containing 1% bovine serum albumin and 5 µM Fura-2AM fluorescent Ca^2+^ indicator dye. Intracellular Ca^2+^ concentrations were measured with an Incyte IM system (Intracellular Imaging Inc, Cincinnati, OH, USA). Fura-2 fluorescence (recorded every 2–5 s) was excited alternately by 340 and 380 nm light, while the emitted fluorescence was detected at 510 nm by selecting at least 1000 pixels within the arteriolar wall. Arteriolar Ca^2+^ concentrations were assessed via the Fura-2 fluorescence ratio (F_340/380_), and in these assays the outer arteriolar diameters were determined for each recorded image. The exact dimensions of the sampling region depended on the ongoing treatment: and it was variable in different vessels. The average dimensions of the sampling region were 285±15 µm×105±6 µm.

### Data analysis and statistical procedures

The diameters of arterioles are shown as means±SEM. Arteriolar constriction was expressed as the change in the baseline initial diameter (id, immediately before the addition of H_2_O_2_) as a percentage of the baseline diameter measured at an intraluminal pressure of 80 mmHg. Arteriolar dilation was calculated as the percentage change from the baseline id (immediately before the addition of H_2_O_2_) to the “passive” diameter in the absence of extracellular Ca^2+^. Statistical analyses were performed with GraphPad Prism 5.0 Software (La Jolla, CA, USA) by the Student's *t*-test and by ANOVA (Dunnett's *post hoc* test). *P*<0.05 was considered statistically significant.

## Results

### H_2_O_2_-induced arteriolar responses

**Figure 1:**
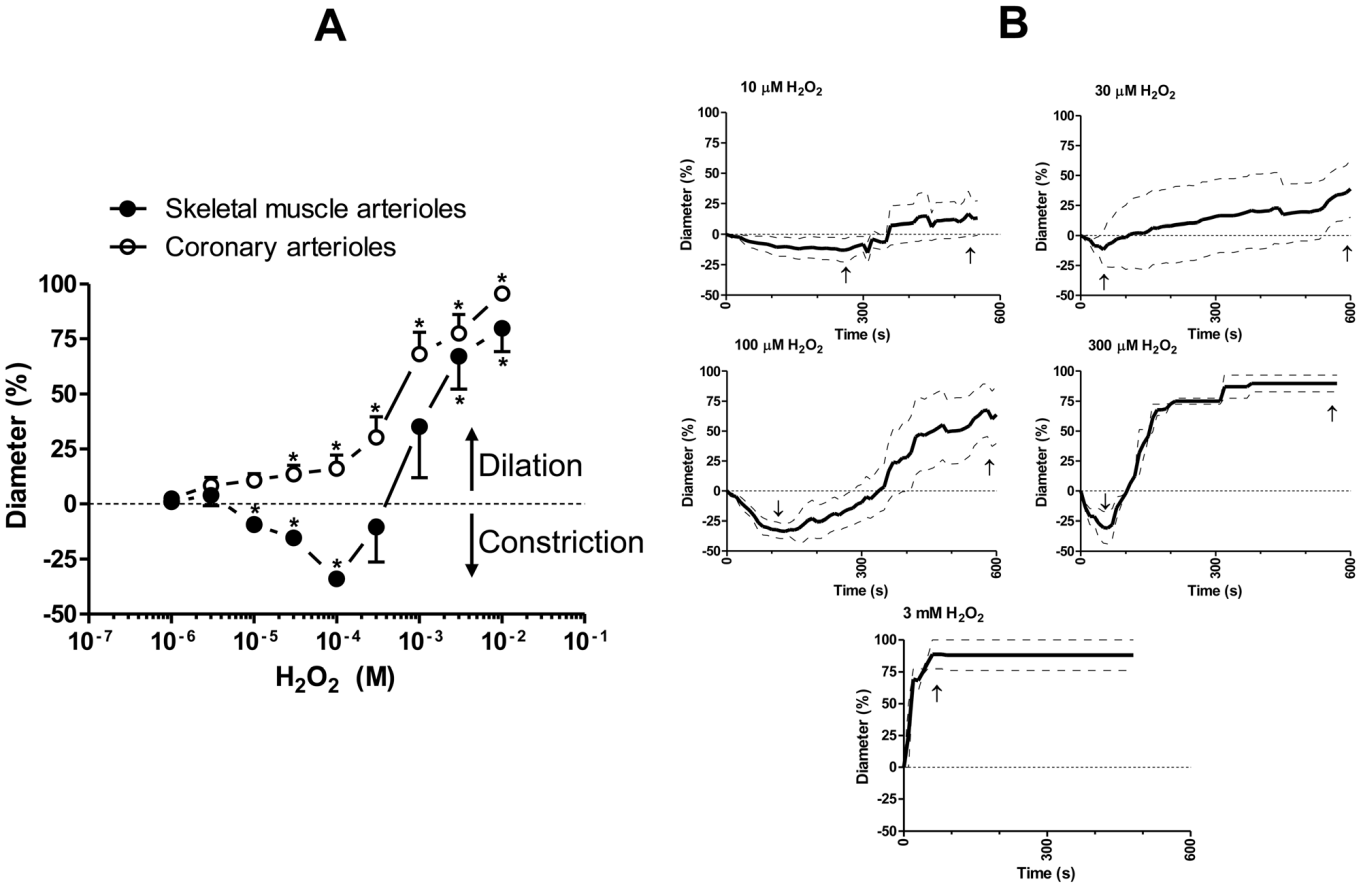
Effects of H_2_O_2_ on arterioles isolated from skeletal muscle and heart. H_2_O_2_ (1 µM–10 mM) was added to isolated, cannulated, skeletal muscle (initial diameter (id: 191±17 µm, n = 6 arterioles from 6 different animals) or coronary arterioles (id: 110±18 µm, n = 7 arterioles from 7 different animals) with intact endothelium. The arteriolar diameter was recorded and concentration-response (cumulative application) relationships were determined (panel A). Changes in relative arteriolar diameter are shown. Relative diameter changes during vasodilations were expressed as percentages of the difference between the maximum passive diameter (maximum dilation: 100%, determined in the absence of extracellular Ca^2+^) and initial diameter with positive values, while during constrictions they were expressed relative to the initial diameter (illustrated at 0% on the y axis) with negative values. Asterisks denote significant differences from the initial values. The kinetics of H_2_O_2_-evoked responses was studied in isolated skeletal muscle arterioles (panel B; means±SEM with solid and dashed lines, respectively). The effects of the indicated concentrations of H_2_O_2_ were recorded for 600 s in the continuous presence of H_2_O_2_ (n = 3–5 arterioles at each concentration from 11 different animals). The positions of maximum constrictions and dilations are illustrated by arrows.

Increasing concentrations of H_2_O_2_ evoked a concentration-dependent biphasic effect in the skeletal muscle arterioles: lower concentrations (10–100 µM) of H_2_O_2_ produced vasoconstriction (maximum at 100 µM, 34±3% constriction, *P*<0.001 *vs.* id, Fig. 1A, Table 1), whereas higher concentrations (3–10 mM) of H_2_O_2_ resulted in vasodilation (maximum at 10 mM, 80±11% dilation, *P*<0.001 *vs.* id). In contrast, H_2_O_2_ evoked only vasodilation in the coronary arterioles (maximum at 10 mM, 96±3% dilation, *P* = 0.01). The kinetics of the H_2_O_2_-evoked changes in the diameter of the skeletal muscle arterioles was also tested. Although the H_2_O_2_-evoked vasoconstrictions were mostly transient, vasoconstrictions at lower H_2_O_2_ concentrations (10 µM and 30 µM) were not followed by significant vasodilations (Fig. 1B). In contrast, 100 µM or 300 µM H_2_O_2_ caused time-dependent biphasic changes: after the initial vasoconstriction, a substantial vasodilation developed. Application of 3 mM H_2_O_2_ resulted in substantial vasodilation without initial vasoconstriction.

**Table 1:** Effects of different inhibitors and endothelium removal on the H_2_O_2_-induced responses.

Type of arteriole	Rat skeletal muscle arterioles											Rat coronary arterioles	
Treatment	Control	None/Control	SQ-29548	Indomethacin	7,7-Dimethyl-(5Z,8Z) eicosadienoic acid	Chelerythrine	U-73122	Src inhibitor-1	Celecoxib	SC-560	NS-398	Control	SQ-29548
No. of experiments	6	7	5	5	5	5	4	5	4	5	3	7	5
Initial diameter	191±17	110±18	109±12	111±2	130±11	121±12	133±3	138±11	148±13	122±9	156±8	110±18	109±12
Diameter after inhibitor	-	-	108±12	111±3	130±11	164±11	126±10	143±12	146±13	113±14	155±8	-	108±12
Diameter after 100 µM H_2_O_2_	128±15	128±20	117±18	130±4	120±11	157±12 *	132±18	133±14 *	135±16	131±17*	142±9*	128±20	117±18
Diameter after 10 mM H_2_O_2_	248±7	200±25	142±13	151±3 *	175±8 *	179±5 *	175±12 *	187±5 *	180±11*	191±7*	215±13*	200±25	142±13
Passive diameter	261±8	205±27	143±12	156±5 *	176±8 *	185±4 *	179±12*	190±4 *	185±11*	200±4*	218±13*	205±27	143±12

Effects of various treatments on the diameter of isolated, cannulated, pressurized (80 mmHg) arterioles of the rat. The tissue sources of the arteriolar beds are indicated (coronary arterioles or skeletal muscle arterioles). Diameters are shown as means±S.E.M. in absolute values (µm). The number of experiments performed is also indicated. Arteriolar diameters are shown at the beginning of the experiments (initial diameter) and after treatment with 100 µM (maximum constrictor dose in the control) or 10 mM (maximum dilator dose in the control) H_2_O_2_. The effects of preincubations with the inhibitors (diameter after the inhibitor) and the maximum diameter of the vessels (passive diameter) are also shown. Significant effects of the treatments on the arteriolar diameters are indicated by asterisks (paired t-test relative to the initial diameter).

### Role of the endothelium in H_2_O_2_-induced vasoconstriction

**Figure 2:**
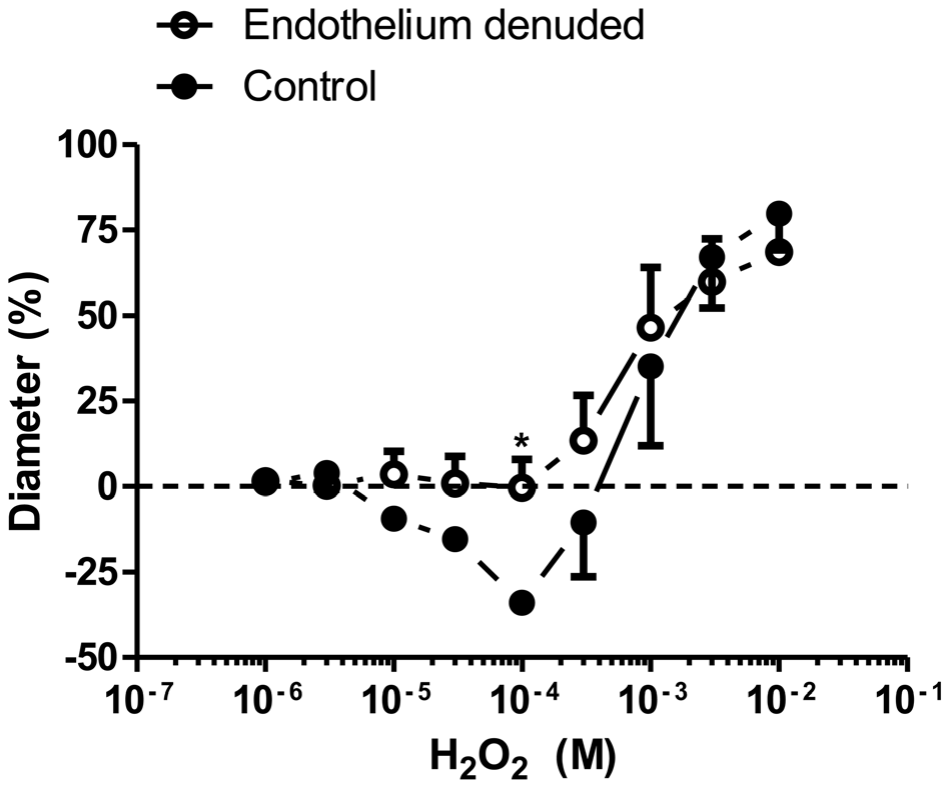
H_2_O_2_-induced vasoconstrictions are mediated by the endothelium in skeletal muscle arterioles. H_2_O_2_ concentration-response relationships were determined (as given in Fig. 1A) in intact (control, closed symbols, n = 6 from 6 different animals) and endothelium-denuded arterioles (id: 131±10 µm, open symbols, n = 5 arterioles from 5 different animals). The asterisk denotes a significant difference from the control.

The H_2_O_2_-induced constriction was abolished in the endothelium-denuded skeletal muscle arterioles (0±8% constriction at 100 µM H_2_O_2_, *P* = 0.03 *vs.* control; Fig. 2A), but the dilations were not affected (69±10% dilation at 10 mM H_2_O_2_).

### H_2_O_2_ stimulated endothelial signalling processes, leading to the activation of COX

**Figure 3:**
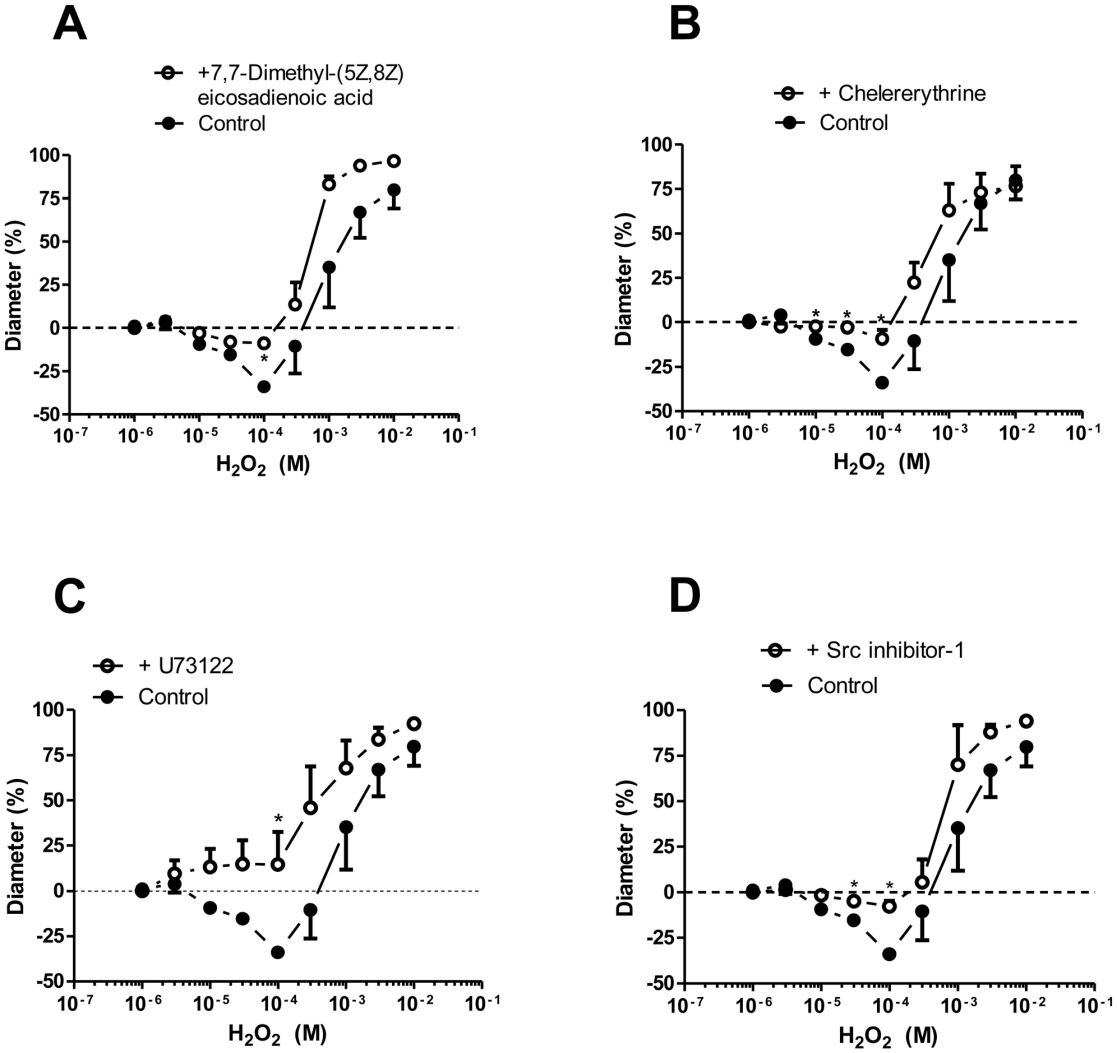
Endothelial mechanisms of H_2_O_2_-evoked vasoconstriction of skeletal muscle arterioles. Arteriolar diameter was recorded in response to H_2_O_2_ without pretreatment (control, as given in Fig. 1A, closed symbols) or after test incubations (open symbols) for at least 15 min in the presence of PLA inhibitor 7,7-dimethyl-(5Z,8Z)-eicosadienoic acid (100 µM, n = 5 arterioles from 5 different animals, id:130±11 µm; panel A), or in the presence of PKC inhibitor chelerythrine (10 µM, n = 5 arterioles from 5 different animals, id: 164±11 µm; panel B), or in the presence of PLC inhibitor U-73122 (10 µM, n = 4 arterioles from 4 different animals, id: 126±10 µm; panel C), or in the presence of Src kinase inhibitor Src inhibitor-1 (5 µM, n = 5 arterioles from 5 different animals, id: 143±12 µm; panel D). Asterisks denote significant differences from the control.

The H_2_O_2_-evoked vasoconstriction was inhibited by the application of the PLA antagonist (7,7-dimethyl-(5Z,8Z)-eicosadienoic acid, 7±2% constriction, *P<*0.005 *vs.* control; Fig. 3A), the PKC antagonist (chelerythrine, 9±4% constriction at 100 µM H_2_O_2_, *P<*0.005 *vs.* control; Fig. 3B), the PLC inhibitor (U-73122, 15±18% dilation, *P<*0.05 *vs.* control, Fig. 3C) or the Src kinase antagonist (Src inhibitor-1, 8±3% vasoconstriction, *P<*0.005 *vs.* control; Fig. 3D).

### Effects of non-selective and selective COX inhibition on H_2_O_2_-induced arteriolar responses

**Figure 4:**
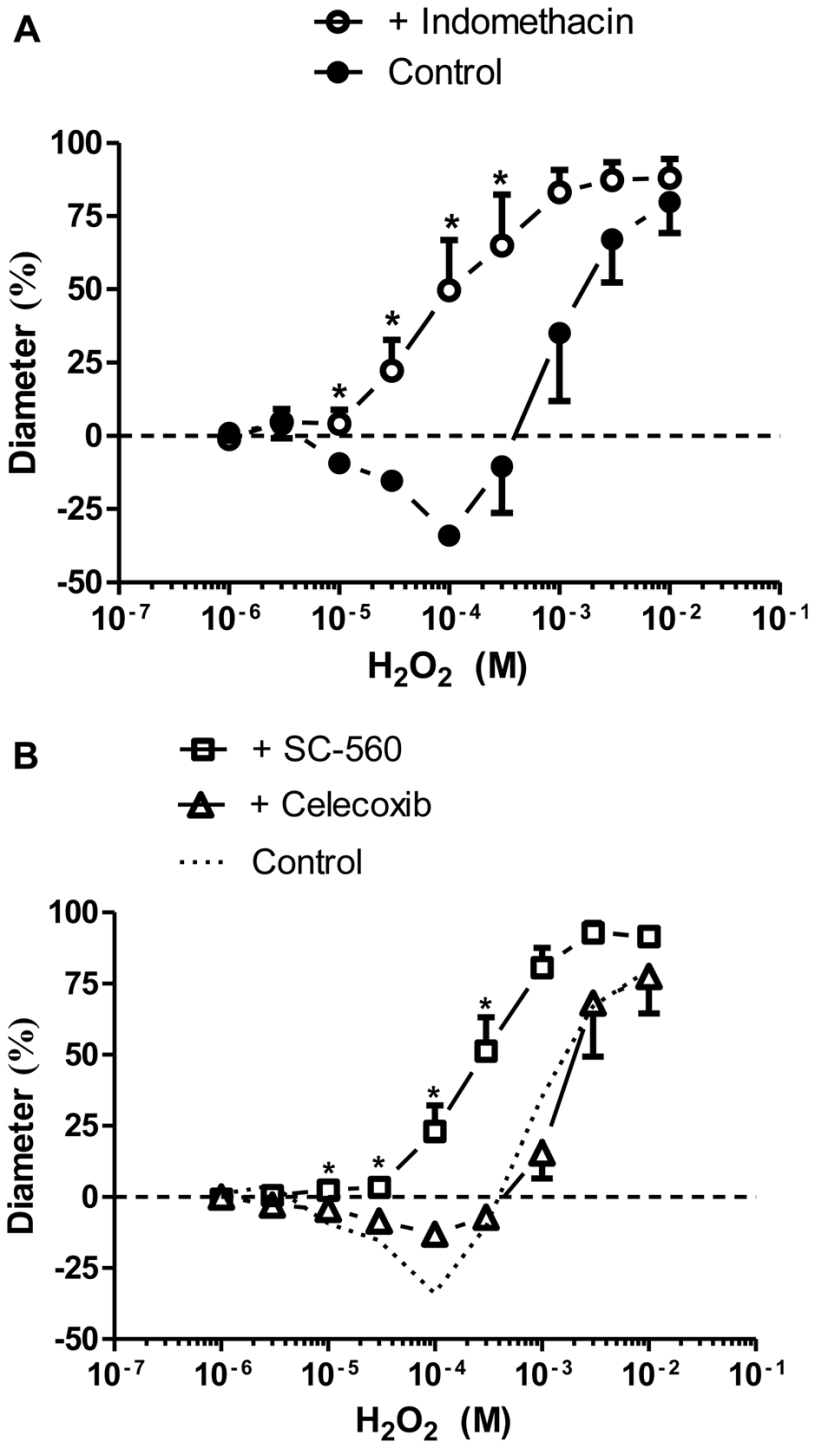
H_2_O_2_-induced vasoconstriction is mediated by COX-1. Arteriolar constrictions (control, as given in Fig. 1A, closed symbols) were prevented in the presence of the non-specific COX inhibitor indomethacin (10 µM, n = 5 arterioles from 4 different animals, preincubation for 30 min, id: 111±3 µm, open symbols; panel A). Panel B: The roles of COX isoforms in H_2_O_2_-evoked responses were studied by comparing vascular diameters in the absence of COX inhibitors (dotted line) with those in the presence of COX-1 inhibitor SC-560 (1 µM, n = 5 arterioles from 3 different animals, id: 113±14 µm; open squares) or with COX-2 inhibitor celecoxib (3 µM, n = 4 arterioles from 4 different animals, id: 146±13 µm; open triangles). Asterisks denote significant differences from the control.

The H_2_O_2_-induced constrictions were converted to dilations in the presence of a non-selective COX inhibitor (indomethacin, 41±17% dilation at 100 µM H_2_O_2_, *P<*0.005 *vs.* control; Fig. 4A). In separate experiments, we investigated the specific roles of COX-1 and COX-2 in the mediation of the H_2_O_2_-evoked vascular responses. It emerged that the selective COX-1 inhibitor SC-560 abolished the constriction induced by H_2_O_2_ (23±9% dilation at 100 µM H_2_O_2,_ *P<*0.05 *vs.* control; Fig. 4B) and converted it to dilation, whereas the inhibitory effect of the COX-2 antagonist celecoxib was not significant (13±4% constriction at 100 µM H_2_O_2,_ *P>*0.05 *vs.* control). Moreover, another COX-2 specific antagonist, NS-398 (10 µM, n = 3 arterioles from 3 different animals), did not prevent the H_2_O_2_-evoked vasoconstrictions either (8±1% constriction at 100 µM H_2_O_2_, *P*>0.05 *vs.* control; Figure S1).

### H_2_O_2_-evoked effector mechanisms leading to vasconstrictive responses

**Figure 5:**
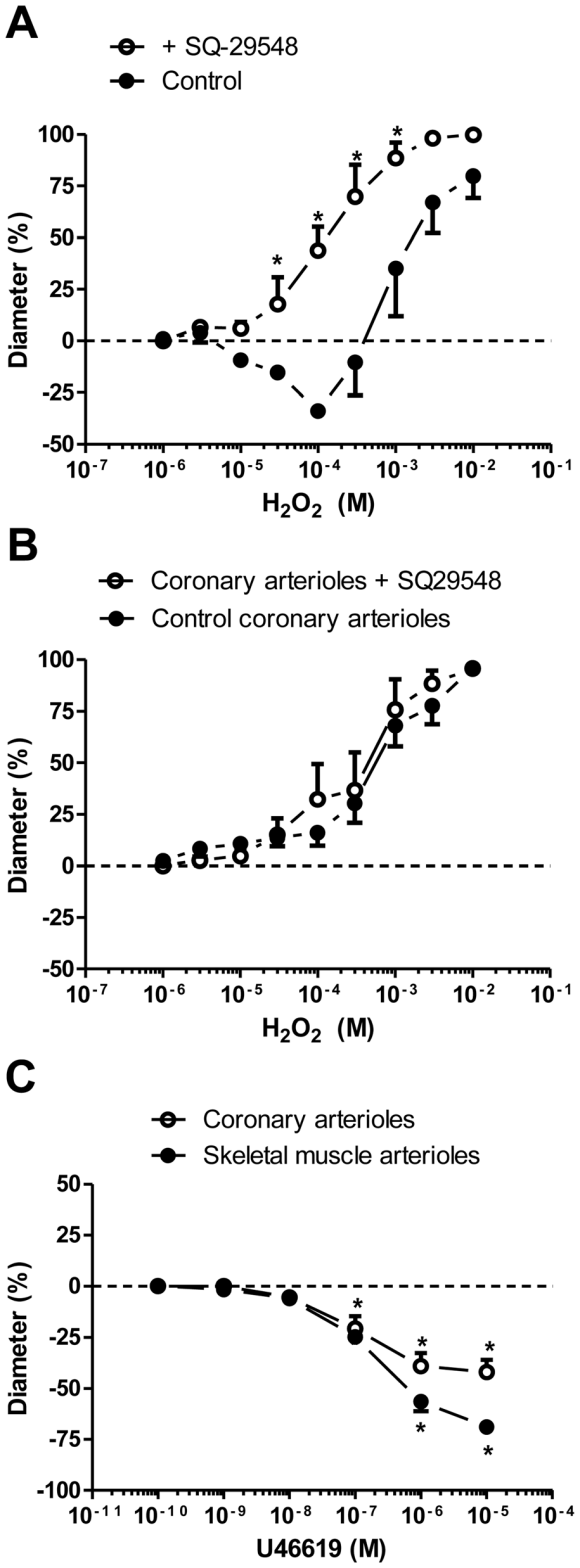
H_2_O_2_-induced vasoconstriction is mediated by TXA2. The role of TXA2 receptors was tested by comparing H_2_O_2_-induced vascular responses under control conditions (closed symbols) with those in the presence of TXA2 receptor antagonist SQ-29548 (1 µM, n = 10 arterioles from 10 different animals, 15-min preincubation) in skeletal muscle arterioles (panel A, open symbols; id: 133±7 µm, asterisks denote significant differences from the control) and in coronary arterioles (panel B, open symbols; id: 108±12 µm). Panel C: The presence of functional TXA2 receptors was verified by the application of TXA2 receptor agonist U46619 (0.1 nM–10 µM) in skeletal muscle (closed symbols; id: 189±7 µm, n = 5 arterioles from 5 different animals) and coronary arterioles (open symbols; id: 119±12 µm, n = 5 arterioles from 5 different animals). Asterisks denote significant differences from the initial diameter.

The H_2_O_2_-evoked vasoconstriction in the skeletal muscle arterioles was abolished and converted to dilation (36±11% dilation at 100 µM H_2_O_2_, *P<*0.005 *vs.* control; Fig. 5A) by TXA2 receptor inhibition (SQ-29548). In contrast, the same treatment did not affect the H_2_O_2_-evoked dilation in the coronary arterioles (96±2% dilation at 10 mM H_2_O_2_; Fig. 5B). Activation of the TXA2 receptors with the stable analogue of TXA2, U46619, resulted in constriction of both the skeletal muscle (69±2%, n = 5, *P<*0.002 *vs*. id; Fig. 5C) and the coronary arterioles (42±6%, *P* = 0.002 *vs*. id; Fig. 5C).

### Characterization of H_2_O_2_-evoked changes in intracellular Ca^2+^ concentrations of vascular smooth muscle cells

**Figure 6:**
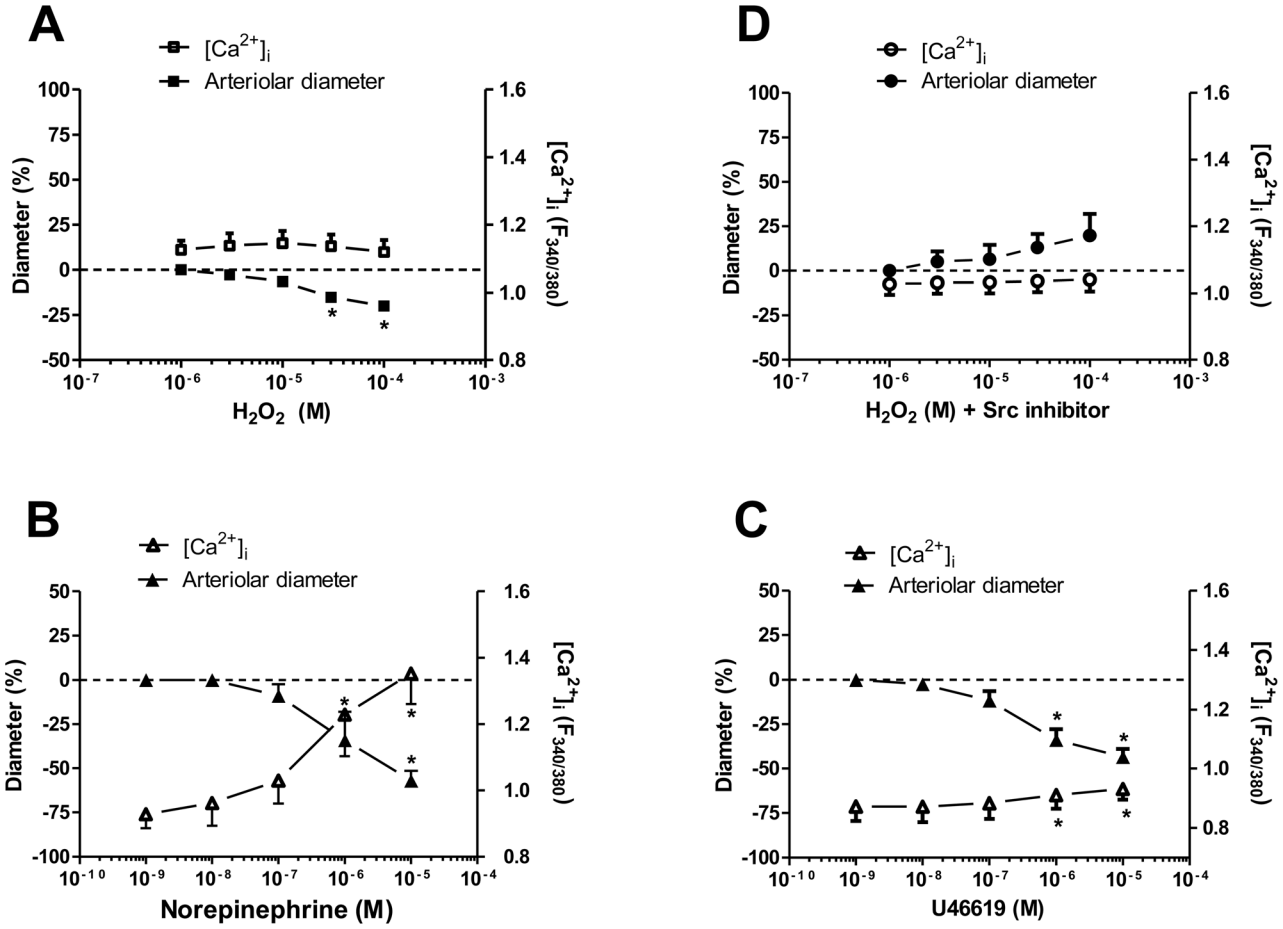
H_2_O_2_ increases the Ca^2+^ sensitivity of force production in vascular smooth muscle cells. The changes in intracellular Ca^2+^ levels and arteriolar diameters were studied in skeletal muscle arterioles under control conditions (panel A; n = 5 arterioles from 3 different animals), or after treatment with norepinephrine (panel B; n = 5 arterioles from 3 different animals), or by addition of the TXA2 receptor agonist U46619 (0.1 nM–10 µM; panel C; n = 5 arterioles from 4 different animals). Experiments were also performed in the presence of H_2_O_2_ together with Src kinase inhibitor, (Src inhibitor-1, 5 µM n = 4 arterioles from 3 different animals, 20-min preincubation; panel D). Asterisks denote significant differences from the initial values.

The H_2_O_2_-evoked vasoconstriction was not accompanied by significant changes in the F_340/380_ ratio signal in the range of H_2_O_2_ concentrations between 1 µM and 100 µM (Fig. 6A). However the norepinephrine (10 µM)-induced vasoconstriction was accompanied by a significant increase in F_340/380_ (from 0.96±0.04 to 1.36±0.07, *P* = 0.001; Fig. 6B). Moreover, the U46619-evoked peak in F_340/380_ was significantly smaller than that evoked by norepinephrine (0.93±0.04 *vs.* 1.36±0.07, respectively, *P*<0.05) despite their largely comparable vasoconstrictive responses (to 44±5% *vs.* 57±6%, respectively, *P*>0.05; Fig. 6C). In another set of experiments, the H_2_O_2_-evoked changes in vascular diameter and Ca^2+^ concentration were measured in the presence of an Src kinase inhibitor (Src inhibitor-1), where vasoconstriction was inhibited by this inhibitor, and F_340/380_ did not change (Fig. 6D). In arterioles with intact endothelium the acetylcholine-induced vasodilation was accompanied by a significant decrease in F_340/380_ (from 1.05±0.05 to 0.89±0.04, *P*<0.05, n = 5).

## Discussion

As far as we are aware this is the first study that has revealed the signalling mechanisms of H_2_O_2_-induced vasoconstriction in the skeletal muscle arterioles of the rat. Besides confirming some steps identified earlier in different vascular preparations, we have now supplemented the signalling cascade with additional molecular interactions. Thus, we have shown that H_2_O_2_ promotes endothelial Src activation and that it leads ultimately to an increased Ca^2+^ sensitivity of force production in vascular smooth muscle cells.

A number of attempts have been made to investigate the mechanism of H_2_O_2_-evoked vasodilation [8], [36], [39], [40], but much less is known as regards the mechanism of H_2_O_2_-evoked vasoconstriction. H_2_O_2_ can modulate the vascular diameter in the rat renal artery [31], the canine basilar artery [50], the porcine coronary arterioles [38] and the rabbit aorta [37] in an endothelium-dependent manner. It may also display endothelium-independent effects in human coronary arterioles [26], canine coronary arterioles [51] and the rat aorta [29]. In the present study, H_2_O_2_-induced vasoconstriction was completely inhibited by endothelium denudation or by inhibition of the TXA2 receptor. Our observations suggest that H_2_O_2_ causes the generation of TXA2 in the endothelium, leading to vasoconstriction [31]–[33], and also that H_2_O_2_ may elicit endothelium-dependent dilation in skeletal muscle arterioles when the TXA2-mediated vasoconstriction is blocked. In contrast, H_2_O_2_-evoked vasodilation in the coronary arterioles was not influenced by a TXA2 inhibitor, although the activation of TXA2 receptors with U46619 resulted in vasoconstriction in both the coronary and the skeletal muscle arterioles. These results suggest that TXA2 receptors are present in both types of vessel, but H_2_O_2_ activates different signalling pathways. It evokes TXA2 synthesis and release from endothelial cells in the skeletal muscle arterioles, but has no such effect in the coronary arterioles.

PLA is responsible for the generation of AA (the substrate of COX) in various vascular preparations [52]. In our study, H_2_O_2_-evoked vasoconstriction was inhibited in the presence of the PLA antagonist (7,7-dimethyl-(5Z,8Z)-eicosadienoic acid, 100 µM), suggesting a role for PLA in the H_2_O_2_-induced vasomotor response. This observation is in accordance with the findings reported by Gao *et al.* on rat mesenteric arterioles [35]. The activation of PLA can be a consequence of PKC-mediated phosphorylation [53]. Indeed, preincubation of skeletal muscle arterioles with the PKC antagonist chelerythrine (10 µM) resulted in a significantly reduced H_2_O_2_-evoked constriction. PKC can be activated by the diacylglycerols released by PLC [54], and inhibition of PLC by U73122 (10 µM) resulted in a significantly decreased H_2_O_2_-mediated vasoconstriction. It might be argued that inhibition of the PKC pathway (*e.g.* PLC and PKC inhibition) can affect TXA2 receptor stimulation-evoked constrictions independently of the endothelial effects of H_2_O_2_. However, PLC inhibition was without effects on the constrictions evoked by the TXA2 receptor agonist U46619 (Figure S2), suggesting an upstream (endothelial) target in H_2_O_2_-mediated constriction.

The H_2_O_2_-evoked activation of PLC was earlier shown to be mediated by Src kinase in mouse embryonic fibroblasts [55]. Indeed, the constrictor effects of H_2_O_2_ in skeletal muscle arterioles were inhibited in the presence of an Src kinase antagonist. Moreover, H_2_O_2_-evoked vasoconstriction was completely inhibited by the non-specific COX antagonist indomethacin. These results are in line with previous findings [8], [29], [31], [35], [56], [57]. Furthermore, the H_2_O_2_-induced vasoconstriction was also fully inhibited in the presence of a specific COX-1 antagonist, while it was not influenced significantly by a specific COX-2 antagonist, suggesting a prominent role of COX-1 in H_2_O_2_-evoked vasoconstriction.

Taken together, the H_2_O_2_-induced constriction component was largely abolished by inhibitors of PLA, PKC, PLC and Src kinases, indicating a complex network of intracellular signalling in the H_2_O_2_ response. Interestingly, H_2_O_2_-evoked vasoconstriction was also prevented in the absence of endothelium. These findings, together with concordant previous observations by others [31]-[33], implicate a sequence of signalling events in the endothelial layer during H_2_O_2_-evoked vasoconstrictions. Nevertheless, alternative mechanisms cannot be excluded.

TXA2 receptors are expressed in numerous cell types, including vascular smooth muscle cells [58]. TXA2 receptors can couple with G_q_ protein, thereby activating the PLC pathway, giving rise to Ca^2+^ release and PKC activation (a Ca^2+^-dependent pathway) [59], [60]. However, TXA2 also binds to G_12_ proteins [60], leading to the activation of Rho-kinase-mediated signalling (a Ca^2+^-independent pathway), and hence to Ca^2+^ sensitization of the contractile protein machinery [59]. Nevertheless, G_12_ proteins may also evoke vasoconstriction by promoting Ca^2+^ entry through another Ca^2+^-dependent mechanism, as has been demonstrated in the rat caudal arterial smooth muscle [61]. Our experimental results indicated that H_2_O_2_-evoked vasoconstrictions were not accompanied by significant increases in intracellular Ca^2+^ concentration. In contrast, the treatment with norepinephrine increased the intracellular Ca^2+^ concentration in parallel with a significant decrease in arteriolar diameter. In comparison, the TXA2 receptor agonist U46619-evoked vasoconstriction was accompanied by a significantly lower increase in intracellular Ca^2+^ concentration than that evoked by norepinephrine, supporting our hypothesis that H_2_O_2_ increases the Ca^2+^ sensitivity of the vascular smooth muscle, rather than stimulating Ca^2+^ entry into smooth muscle cells. Similar conclusions were reached in previous studies, where the H_2_O_2_-induced constriction of isolated rabbit [32], [46] or porcine (36) pulmonary arterioles was not influenced by extracellular Ca^2+^ removal. Although the explanation of the apparent increase in vascular Ca^2+^ sensitivity is beyond the scope of this study, we speculate that the potential mechanism may involve the inhibition of myosin light chain phosphatase via Rho-associated kinase (ROCK) or PKC, leading to increased phosphorylation of LC20 (myosin regulatory light chain) [62]. Alternatively, vascular Ca^2+^ sensitization of constriction could be elicited by dynamic regulation of the actin cytoskeleton by PKC and ROCK [63].

It is rather difficult to estimate the real concentration of H_2_O_2_ in vascular beds *in vivo*. Nevertheless, it has been shown that in certain pathological conditions it may reach relatively high levels (up to about 0.3 mM) [8], [17], [18]. In this study, the use of even higher concentrations of H_2_O_2_ (up to 10 mM) allowed us to characterize the biphasic vascular effects of H_2_O_2_. Lower concentrations of H_2_O_2_ evoked vasodilation in coronary arterioles, but elicited the constriction of skeletal muscle arterioles. This is consistent with the previous finding an important regulatory role of H_2_O_2_ as an EDHF in the coronary microcirculation [20], [21], [26], and the conclusion that, H_2_O_2_ cannot be regarded as an EDHF in skeletal muscle arterioles under physiological conditions [8]. It is unclear whether H_2_O_2_ concentrations reach levels high enough to evoke vasodilation and hence to increase the skeletal muscle blood flow under pathological conditions (*e.g.* inflammation).

**Figure 7:**
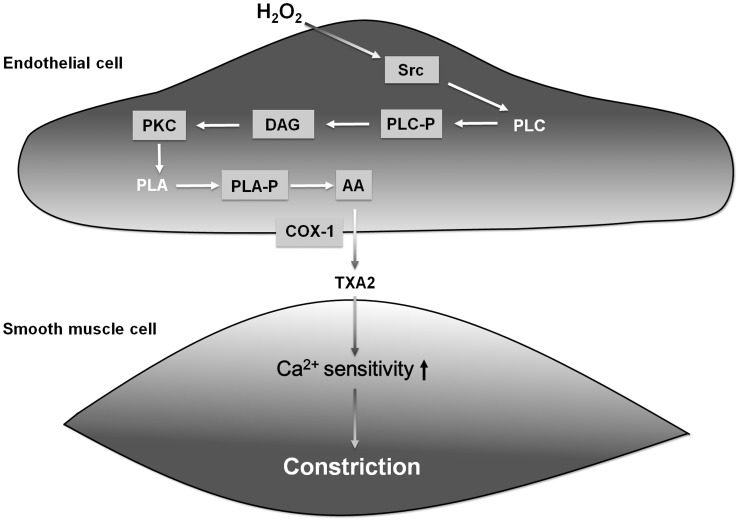
Proposed molecular mechanisms of H_2_O_2_-evoked vasoconstriction, based on the present study. H_2_O_2_ may induce both vasodilation and vasoconstriction, depending on the applied H_2_O_2_ concentration, vessel type, species and experimental protocol (*e.g.* exposure time). Our data imply that H_2_O_2_ elicits vasoconstriction by activating Src kinase, which activates the phospholipase C (PLC), protein kinase (PKC), phospholipase A (PLA) and cyclooxygenase (COX) pathway, leading to the production of thromboxane A2 (TXA2), which increases the Ca^2+^ sensitivity of the vascular smooth muscle in skeletal muscle arterioles of the rat (DAG: diacylglycerol).

The findings of the present study suggest that H_2_O_2_ activates an endothelial signalling pathway, leading to the synthesis of TXA2, which then activates its receptors of smooth muscle cells, leading to an increase in the Ca^2+^ sensitivity of their contractile protein machinery. Figure 7 summarizes the detailed mechanisms identified or confirmed in the present study that lead to H_2_O_2_-evoked constrictions of the skeletal muscle arterioles. Elucidation of these details of this H_2_O_2_-induced signalling not only adds to our knowledge of H_2_O_2_-induced vasomotor responses, but may also furnish novel molecular targets for the treatment of H_2_O_2_-driven vascular dysfunctions.

## Supporting Information

Figure S1 **Effects of different COX-2 specific inhibitors on H_2_O_2_-induced vasoconstriction.** The lack of the effects of COX-2 in the vasoconstriction evoked by H_2_O_2_ was confirmed by using another COX-2-specific inhibitor, NS-398 (10 µM, n = 3 arterioles from 3 different animals, id: 155±8 µm; closed triangles). The effects of celecoxib are indicated by open triangles (3 µM celecoxib, n = 4 arterioles from 4 different animals, id: 146±13 µm); the dotted line denotes the control.
(TIF)

Figure S2 **PLC inhibition had no effects on the constrictions evoked by the TXA2 receptor agonist.** PLC inhibition (10 µM U73122) significantly decreased the constriction evoked by norepinephrine (n = 4 arterioles from 2 different animals, id: 170±10 µm and 154±8 µm; panel A), but did not influence the constrictions evoked by increasing concentrations of the TXA2 receptor agonist U46619 in skeletal muscle arterioles (n = 5 arterioles from 4 different animals, id: 171±10 µm and 154±8 µm; panel B). Means±SEM are plotted. Asterisks denote significant differences from the control.
(TIF)

File S1 **Data in supporting information file.**
(PDF)

## References

[1] Montezano AC, Touyz RM (2012) Molecular mechanisms of hypertension—reactive oxygen species and antioxidants: a basic science update for the clinician. Can J Cardiol 28: 288–295. 22445098 10.1016/j.cjca.2012.01.017

[2] Lacy F, Kailasam MT, O′Connor DT, Schmid-Schonbein GW, Parmer RJ (2000) Plasma hydrogen peroxide production in human essential hypertension: role of heredity, gender, and ethnicity. Hypertension 36: 878–884. 11082160 10.1161/01.hyp.36.5.878

[3] Erdei N, Bagi Z, Edes I, Kaley G, Koller A (2007) H_2_O_2_ increases production of constrictor prostaglandins in smooth muscle leading to enhanced arteriolar tone in Type 2 diabetic mice. Am J Physiol Heart Circ Physiol 292: H649–656. 16997891 10.1152/ajpheart.00596.2006

[4] Shi Y, So KF, Man RY, Vanhoutte PM (2007) Oxygen-derived free radicals mediate endothelium-dependent contractions in femoral arteries of rats with streptozotocin-induced diabetes. Br J Pharmacol 152: 1033–1041. 17767168 10.1038/sj.bjp.0707439 PMC2095103

[5] Hulsmans M, Van Dooren E, Holvoet P (2012) Mitochondrial reactive oxygen species and risk of atherosclerosis. Curr Atheroscler Rep 14: 264–276. 22350585 10.1007/s11883-012-0237-0

[6] Brandes RP, Kreuzer J (2005) Vascular NADPH oxidases: molecular mechanisms of activation. Cardiovasc Res 65: 16–27. 15621030 10.1016/j.cardiores.2004.08.007

[7] Cai H (2005) Hydrogen peroxide regulation of endothelial function: origins, mechanisms, and consequences. Cardiovasc Res 68: 26–36. 16009356 10.1016/j.cardiores.2005.06.021

[8] Cseko C, Bagi Z, Koller A (2004) Biphasic effect of hydrogen peroxide on skeletal muscle arteriolar tone via activation of endothelial and smooth muscle signaling pathways. J Appl Physiol 97: 1130–1137. 15208297 10.1152/japplphysiol.00106.2004

[9] Cai H, Griendling KK, Harrison DG (2003) The vascular NAD(P)H oxidases as therapeutic targets in cardiovascular diseases. Trends Pharmacol Sci 24: 471–478. 12967772 10.1016/S0165-6147(03)00233-5

[10] Nedeljkovic ZS, Gokce N, Loscalzo J (2003) Mechanisms of oxidative stress and vascular dysfunction. Postgrad Med J 79: 195–199 quiz 198–200. 12743334 10.1136/pmj.79.930.195 PMC1742679

[11] Mueller CF, Laude K, McNally JS, Harrison DG (2005) ATVB in focus: redox mechanisms in blood vessels. Arterioscler Thromb Vasc Biol 25: 274–278. 15514203 10.1161/01.ATV.0000149143.04821.eb

[12] Zhang DX, Gutterman DD (2007) Mitochondrial reactive oxygen species-mediated signaling in endothelial cells. Am J Physiol Heart Circ Physiol 292: H2023–2031. 17237240 10.1152/ajpheart.01283.2006

[13] Briones AM, Touyz RM (2010) Oxidative stress and hypertension: current concepts. Curr Hypertens Rep 12: 135–142. 20424957 10.1007/s11906-010-0100-z

[14] Touyz RM (2004) Reactive oxygen species, vascular oxidative stress, and redox signaling in hypertension: what is the clinical significance? Hypertension 44: 248–252. 15262903 10.1161/01.HYP.0000138070.47616.9d

[15] Halliwell B, Gutteridge JM (1984) Oxygen toxicity, oxygen radicals, transition metals and disease. Biochem J 219: 1–14. 6326753 10.1042/bj2190001 PMC1153442

[16] Taniyama Y, Griendling KK (2003) Reactive oxygen species in the vasculature: molecular and cellular mechanisms. Hypertension 42: 1075–1081. 14581295 10.1161/01.HYP.0000100443.09293.4F

[17] Root RK, Metcalf JA (1977) H_2_O_2_ release from human granulocytes during phagocytosis. Relationship to superoxide anion formation and cellular catabolism of H_2_O_2_: studies with normal and cytochalasin B-treated cells. J Clin Invest 60: 1266–1279. 199619 10.1172/JCI108886 PMC372483

[18] Liu X, Zweier JL (2001) A real-time electrochemical technique for measurement of cellular hydrogen peroxide generation and consumption: evaluation in human polymorphonuclear leukocytes. Free Radic Biol Med 31: 894–901. 11585708 10.1016/s0891-5849(01)00665-7

[19] Matoba T, Shimokawa H, Nakashima M, Hirakawa Y, Mukai Y, et al (2000) Hydrogen peroxide is an endothelium-derived hyperpolarizing factor in mice. J Clin Invest 106: 1521–1530. 11120759 10.1172/JCI10506 PMC387255

[20] Yada T, Shimokawa H, Hiramatsu O, Kajita T, Shigeto F, et al (2003) Hydrogen peroxide, an endogenous endothelium-derived hyperpolarizing factor, plays an important role in coronary autoregulation in vivo. Circulation 107: 1040–1045. 12600919 10.1161/01.cir.0000050145.25589.65

[21] Matoba T, Shimokawa H, Morikawa K, Kubota H, Kunihiro I, et al (2003) Electron spin resonance detection of hydrogen peroxide as an endothelium-derived hyperpolarizing factor in porcine coronary microvessels. Arterioscler Thromb Vasc Biol 23: 1224–1230. 12763764 10.1161/01.ATV.0000078601.79536.6C

[22] Koller A, Bagi Z (2004) Nitric oxide and H_2_O_2_ contribute to reactive dilation of isolated coronary arterioles. Am J Physiol Heart Circ Physiol 287: H2461–2467. 15319207 10.1152/ajpheart.00295.2004

[23] Wagenfeld L, von Domarus F, Weiss S, Klemm M, Richard G, et al (2013) The effect of reactive oxygen species on the myogenic tone of rat ophthalmic arteries with and without endothelium. Graefes Arch Clin Exp Ophthalmol 251: 2339–2344. 23744487 10.1007/s00417-013-2387-3

[24] Liu Y, Bubolz AH, Mendoza S, Zhang DX, Gutterman DD (2011) H_2_O_2_ is the transferrable factor mediating flow-induced dilation in human coronary arterioles. Circ Res 108: 566–573. 21233456 10.1161/CIRCRESAHA.110.237636 PMC3108183

[25] Matoba T, Shimokawa H, Kubota H, Morikawa K, Fujiki T, et al (2002) Hydrogen peroxide is an endothelium-derived hyperpolarizing factor in human mesenteric arteries. Biochem Biophys Res Commun 290: 909–913. 11798159 10.1006/bbrc.2001.6278

[26] Miura H, Bosnjak JJ, Ning G, Saito T, Miura M, et al (2003) Role for hydrogen peroxide in flow-induced dilation of human coronary arterioles. Circ Res 92: e31–40. 12574154 10.1161/01.res.0000054200.44505.ab

[27] Liu Y, Zhao H, Li H, Kalyanaraman B, Nicolosi AC, et al (2003) Mitochondrial sources of H_2_O_2_ generation play a key role in flow-mediated dilation in human coronary resistance arteries. Circ Res 93: 573–580. 12919951 10.1161/01.RES.0000091261.19387.AE

[28] Cai H (2005) NAD(P)H oxidase-dependent self-propagation of hydrogen peroxide and vascular disease. Circ Res 96: 818–822. 15860762 10.1161/01.RES.0000163631.07205.fb

[29] Yang ZW, Zheng T, Zhang A, Altura BT, Altura BM (1998) Mechanisms of hydrogen peroxide-induced contraction of rat aorta. Eur J Pharmacol 344: 169–181. 9600652 10.1016/s0014-2999(97)01576-8

[30] Rodriguez-Martinez MA, Garcia-Cohen EC, Baena AB, Gonzalez R, Salaices M, et al (1998) Contractile responses elicited by hydrogen peroxide in aorta from normotensive and hypertensive rats. Endothelial modulation and mechanism involved. Br J Pharmacol 125: 1329–1335. 9863664 10.1038/sj.bjp.0702200 PMC1565706

[31] Gao YJ, Lee RM (2005) Hydrogen peroxide is an endothelium-dependent contracting factor in rat renal artery. Br J Pharmacol 146: 1061–1068. 16231001 10.1038/sj.bjp.0706423 PMC1751245

[32] Sheehan DW, Giese EC, Gugino SF, Russell JA (1993) Characterization and mechanisms of H_2_O_2_-induced contractions of pulmonary arteries. Am J Physiol 264: H1542–1547. 8498568 10.1152/ajpheart.1993.264.5.H1542

[33] Katusic ZS, Schugel J, Cosentino F, Vanhoutte PM (1993) Endothelium-dependent contractions to oxygen-derived free radicals in the canine basilar artery. Am J Physiol 264: H859–864. 8456988 10.1152/ajpheart.1993.264.3.H859

[34] Yang ZW, Zheng T, Wang J, Zhang A, Altura BT, et al (1999) Hydrogen peroxide induces contraction and raises [Ca^2+^]_i_ in canine cerebral arterial smooth muscle: participation of cellular signaling pathways. Naunyn Schmiedebergs Arch Pharmacol 360: 646–653. 10619181 10.1007/s002109900128

[35] Gao YJ, Hirota S, Zhang DW, Janssen LJ, Lee RM (2003) Mechanisms of hydrogen-peroxide-induced biphasic response in rat mesenteric artery. Br J Pharmacol 138: 1085–1092. 12684264 10.1038/sj.bjp.0705147 PMC1573754

[36] Iida Y, Katusic ZS (2000) Mechanisms of cerebral arterial relaxations to hydrogen peroxide. Stroke 31: 2224–2230. 10978056 10.1161/01.str.31.9.2224

[37] Zembowicz A, Hatchett RJ, Jakubowski AM, Gryglewski RJ (1993) Involvement of nitric oxide in the endothelium-dependent relaxation induced by hydrogen peroxide in the rabbit aorta. Br J Pharmacol 110: 151–158. 7693274 10.1111/j.1476-5381.1993.tb13785.x PMC2175976

[38] Thengchaisri N, Kuo L (2003) Hydrogen peroxide induces endothelium-dependent and -independent coronary arteriolar dilation: role of cyclooxygenase and potassium channels. Am J Physiol Heart Circ Physiol 285: H2255–2263. 14613908 10.1152/ajpheart.00487.2003

[39] Barlow RS, White RE (1998) Hydrogen peroxide relaxes porcine coronary arteries by stimulating BKCa channel activity. Am J Physiol 275: H1283–1289. 9746477 10.1152/ajpheart.1998.275.4.H1283

[40] Hayabuchi Y, Nakaya Y, Matsuoka S, Kuroda Y (1998) Hydrogen peroxide-induced vascular relaxation in porcine coronary arteries is mediated by Ca^2+^-activated K^+^ channels. Heart Vessels 13: 9–17. 9923560 10.1007/BF02750638

[41] Rogers PA, Chilian WM, Bratz IN, Bryan RM Jr, Dick GM (2007) H_2_O_2_ activates redox- and 4-aminopyridine-sensitive K_v_ channels in coronary vascular smooth muscle. Am J Physiol Heart Circ Physiol 292: H1404–1411. 17071731 10.1152/ajpheart.00696.2006

[42] Zhang DX, Borbouse L, Gebremedhin D, Mendoza SA, Zinkevich NS, et al (2012) H_2_O_2_-induced dilation in human coronary arterioles: role of protein kinase G dimerization and large-conductance Ca^2+^-activated K^+^ channel activation. Circ Res 110: 471–480. 22158710 10.1161/CIRCRESAHA.111.258871 PMC3272100

[43] Gao YJ, Lee RM (2001) Hydrogen peroxide induces a greater contraction in mesenteric arteries of spontaneously hypertensive rats through thromboxane A(2) production. Br J Pharmacol 134: 1639–1646. 11739239 10.1038/sj.bjp.0704420 PMC1572900

[44] Oeckler RA, Kaminski PM, Wolin MS (2003) Stretch enhances contraction of bovine coronary arteries via an NAD(P)H oxidase-mediated activation of the extracellular signal-regulated kinase mitogen-activated protein kinase cascade. Circ Res 92: 23–31. 12522117 10.1161/01.res.0000051860.84509.ce

[45] Ardanaz N, Beierwaltes WH, Pagano PJ (2007) Comparison of H_2_O_2_-induced vasoconstriction in the abdominal aorta and mesenteric artery of the mouse. Vascul Pharmacol 47: 288–294. 17900993 10.1016/j.vph.2007.08.007

[46] Pelaez NJ, Braun TR, Paul RJ, Meiss RA, Packer CS (2000) H_2_O_2_ mediates Ca^2+^- and MLC_20_ phosphorylation-independent contraction in intact and permeabilized vascular muscle. Am J Physiol Heart Circ Physiol 279: H1185–1193. 10993783 10.1152/ajpheart.2000.279.3.H1185

[47] Feher A, Rutkai I, Beleznai T, Ungvari Z, Csiszar A, et al (2010) Caveolin-1 limits the contribution of BK(Ca) channel to EDHF-mediated arteriolar dilation: implications in diet-induced obesity. Cardiovasc Res 87: 732–739. 20299334 10.1093/cvr/cvq088 PMC2920808

[48] Czikora A, Lizanecz E, Bako P, Rutkai I, Ruzsnavszky F, et al (2012) Structure-activity relationships of vanilloid receptor agonists for arteriolar TRPV1. Br J Pharmacol 165: 1801–1812. 21883148 10.1111/j.1476-5381.2011.01645.x PMC3372831

[49] Kandasamy K, Bezavada L, Escue RB, Parthasarathi K (2013) Lipopolysaccharide induces endoplasmic store Ca^2+^-dependent inflammatory responses in lung microvessels. PLoS One 8: e63465. 23675486 10.1371/journal.pone.0063465 PMC3651233

[50] Yang ZW, Zhang A, Altura BT, Altura BM (1998) Endothelium-dependent relaxation to hydrogen peroxide in canine basilar artery: a potential new cerebral dilator mechanism. Brain Res Bull 47: 257–263. 9865858 10.1016/s0361-9230(98)00120-8

[51] Rogers PA, Dick GM, Knudson JD, Focardi M, Bratz IN, et al (2006) H_2_O_2_-induced redox-sensitive coronary vasodilation is mediated by 4-aminopyridine-sensitive K^+^ channels. Am J Physiol Heart Circ Physiol 291: H2473–2482. 16751285 10.1152/ajpheart.00172.2006

[52] Wong MS, Vanhoutte PM (2010) COX-mediated endothelium-dependent contractions: from the past to recent discoveries. Acta Pharmacol Sin 31: 1095–1102. 20711228 10.1038/aps.2010.127 PMC4002305

[53] Akiba S, Sato T (2004) Cellular function of calcium-independent phospholipase A2. Biol Pharm Bull 27: 1174–1178. 15305016 10.1248/bpb.27.1174

[54] Meier M, King GL (2000) Protein kinase C activation and its pharmacological inhibition in vascular disease. Vasc Med 5: 173–185. 11104300 10.1177/1358836X0000500307

[55] Wang XT, McCullough KD, Wang XJ, Carpenter G, Holbrook NJ (2001) Oxidative stress-induced phospholipase C-gamma 1 activation enhances cell survival. J Biol Chem 276: 28364–28371. 11350969 10.1074/jbc.M102693200

[56] Gao Y, Vanhoutte PM (1993) Products of cyclooxygenase mediate the responses of the guinea pig trachea to hydrogen peroxide. J Appl Physiol 74: 2105–2111. 8335536 10.1152/jappl.1993.74.5.2105

[57] Gil-Longo J, Gonzalez-Vazquez C (2005) Characterization of four different effects elicited by H_2_O_2_ in rat aorta. Vascul Pharmacol 43: 128–138. 15994130 10.1016/j.vph.2005.06.001

[58] Sellers MM, Stallone JN (2008) Sympathy for the devil: the role of thromboxane in the regulation of vascular tone and blood pressure. Am J Physiol Heart Circ Physiol 294: H1978–1986. 18310512 10.1152/ajpheart.01318.2007

[59] Nakahata N (2008) Thromboxane A2: physiology/pathophysiology, cellular signal transduction and pharmacology. Pharmacol Ther 118: 18–35. 18374420 10.1016/j.pharmthera.2008.01.001

[60] Offermanns S, Laugwitz KL, Spicher K, Schultz G (1994) G proteins of the G12 family are activated via thromboxane A2 and thrombin receptors in human platelets. Proc Natl Acad Sci U S A 91: 504–508. 8290554 10.1073/pnas.91.2.504 PMC42977

[61] Wilson DP, Susnjar M, Kiss E, Sutherland C, Walsh MP (2005) Thromboxane A2-induced contraction of rat caudal arterial smooth muscle involves activation of Ca^2+^ entry and Ca^2+^ sensitization: Rho-associated kinase-mediated phosphorylation of MYPT1 at Thr-855, but not Thr-697. Biochem J 389: 763–774. 15823093 10.1042/BJ20050237 PMC1180727

[62] Somlyo AP, Somlyo AV (2003) Ca^2+^ sensitivity of smooth muscle and nonmuscle myosin II: modulated by G proteins, kinases, and myosin phosphatase. Physiol Rev 83: 1325–1358. 14506307 10.1152/physrev.00023.2003

[63] Walsh MP, Cole WC (2013) The role of actin filament dynamics in the myogenic response of cerebral resistance arteries. J Cereb Blood Flow Metab 33: 1–12. 23072746 10.1038/jcbfm.2012.144 PMC3597360

