# Supplementary figures and images for: Hydrogen Peroxide Elicits Constriction of Skeletal Muscle Arterioles by Activating the Arachidonic Acid Pathway
Source: PLoS One. 2014 Aug 5;9(8):e103858. doi: 10.1371/journal.pone.0103858 (PMC4122381; doi:10.1371/journal.pone.0103858)

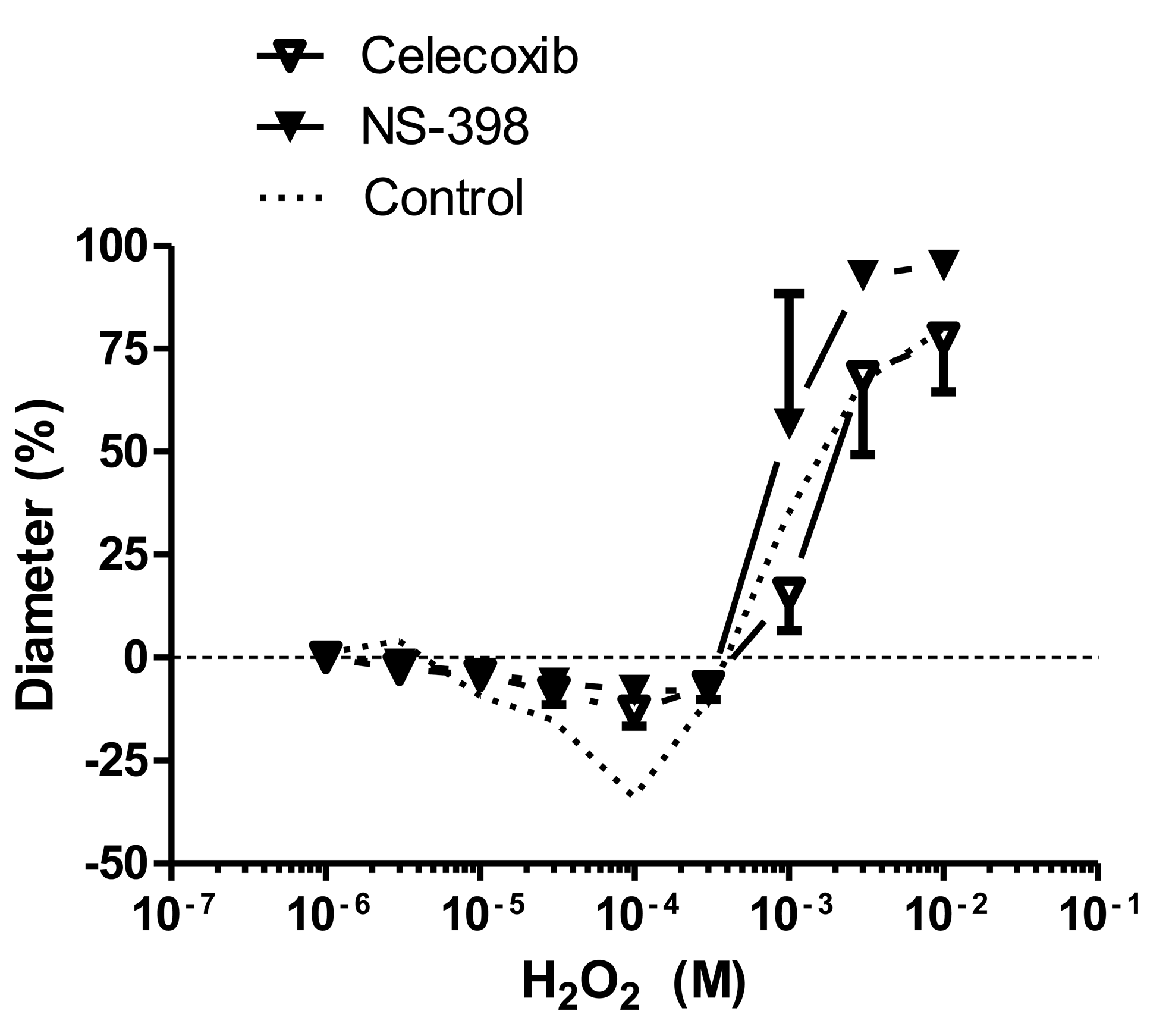

Supplement: Figure S1 — Effects of different COX-2 specific inhibitors on H2O2-induced vasoconstriction. The lack of the effects of COX-2 in the vasoconstriction evoked by H2O2 was confirmed by using another COX-2-specific inhibitor, NS-398 (10 µM, n = 3 arterioles from 3 different animals, id: 155±8 µm; closed triangles). The effects of celecoxib are indicated by open triangles (3 µM celecoxib, n = 4 arterioles from 4 different animals, id: 146±13 µm); the dotted line denotes the control. (TIF) [file pone.0103858.s001.tif]

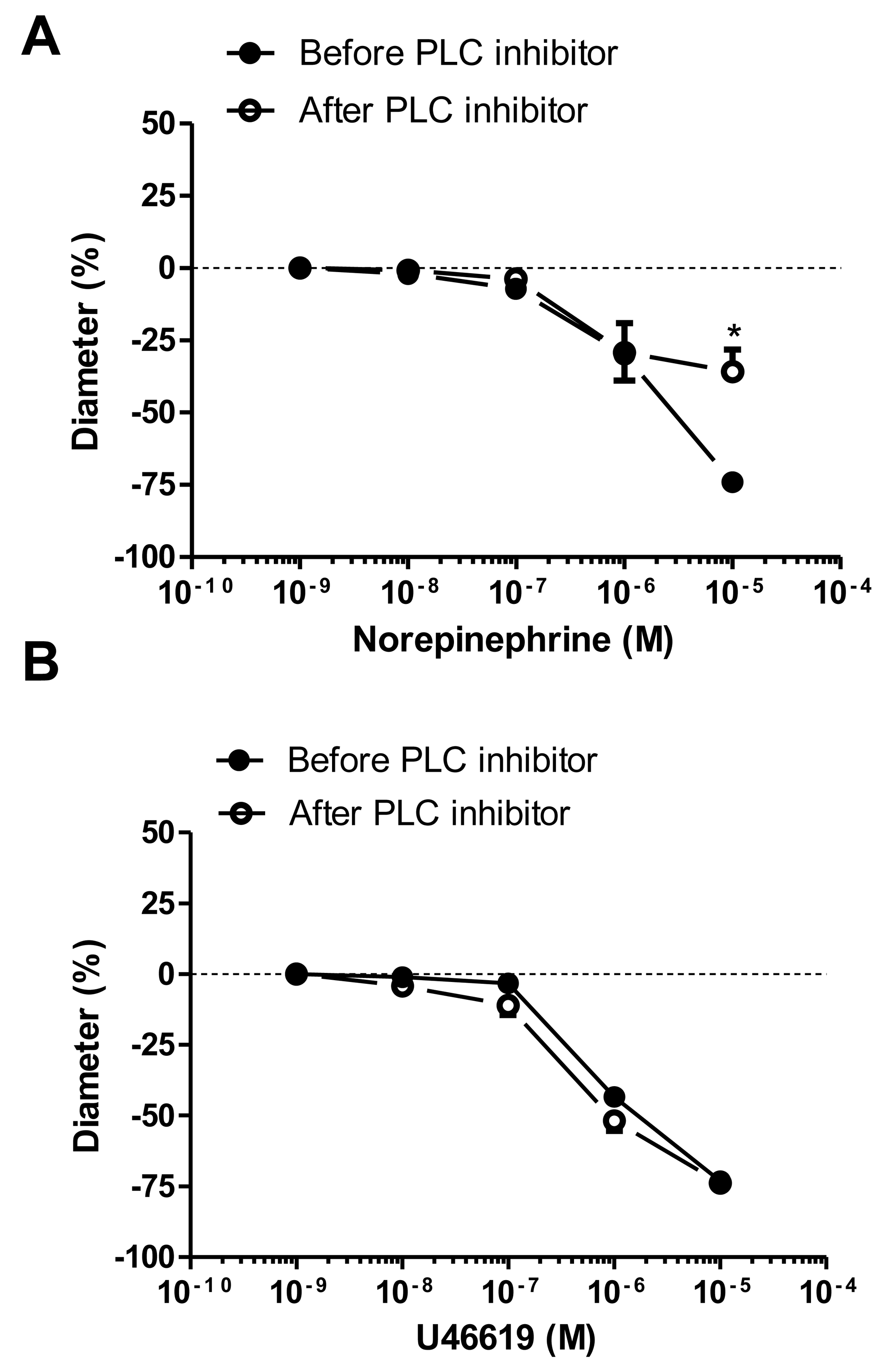

Supplement: Figure S2 — PLC inhibition had no effects on the constrictions evoked by the TXA2 receptor agonist. PLC inhibition (10 µM U73122) significantly decreased the constriction evoked by norepinephrine (n = 4 arterioles from 2 different animals, id: 170±10 µm and 154±8 µm; panel A), but did not influence the constrictions evoked by increasing concentrations of the TXA2 receptor agonist U46619 in skeletal muscle arterioles (n = 5 arterioles from 4 different animals, id: 171±10 µm and 154±8 µm; panel B). Means±SEM are plotted. Asterisks denote significant differences from the control. (TIF) [file pone.0103858.s002.tif]
