# Supplementary material for: Hydrogen Peroxide Elicits Constriction of Skeletal Muscle Arterioles by Activating the Arachidonic Acid Pathway
Source: PLoS One. 2014 Aug 5;9(8):e103858. doi: 10.1371/journal.pone.0103858 (PMC4122381; doi:10.1371/journal.pone.0103858)
Supplement: File S1 — Data in supporting information file. (PDF) [file pone.0103858.s003.pdf]

## Figure 1. Panel A

| **Date (number of experiments)** | | *20100818* | *20100823* | *20100902* | *20100906* | *20100908* | *20100914* | *Average* | *± SEM* |
|---|---|---|---|---|---|---|---|---|---|
| | **Concentration (M)** | **Diameter (μm)** | | | | | | **Diameter (μm)** | |
| | before incubation | | 258.1521 | 272.77082 | 265.16607 | 251.24986 | 234.78947 | 256.4257 | 6.486344 |
| | initial | 222.73592 | 218.92542 | 155.03428 | 203.57592 | 210.96635 | 96.812699 | 184.6751 | 20.22256 |
| **Achetylcholine** | 1.00E-009 | 222.73592 | 218.92542 | 170.84188 | 206.1009 | 210.96635 | 95.840219 | 187.5684 | 19.83664 |
| | 1.00E-008 | 222.73592 | 218.92542 | 170.84188 | 207.99472 | 210.96635 | 98.363303 | 188.3046 | 19.50965 |
| | 1.00E-007 | 232.83765 | 218.92542 | 258.62929 | 250.46074 | 210.59411 | 119.82272 | 215.2117 | 20.47147 |
| | 1.00E-006 | 265.10568 | 228.41133 | 283.50008 | 268.77237 | 257.71132 | 187.75249 | 248.5422 | 14.24825 |
| | 1.00E-005 | 266.37109 | 255.00985 | 272.68275 | 268.77237 | 262.63581 | 212.92972 | 256.4003 | 9.033473 |
| | initial | 229.68718 | 205.6926 | 196.2417 | 246.15293 | 224.61245 | 101.31244 | 200.6166 | 21.13503 |
| **Norepinephrine** | 1.00E-009 | 227.1565 | 206.32511 | 190.55038 | 243.62241 | 224.61245 | 101.31244 | 198.9299 | 20.90281 |
| | 1.00E-008 | 227.1565 | 206.32511 | 172.78623 | 243.62241 | 222.08164 | 90.391016 | 193.7272 | 22.86673 |
| | 1.00E-007 | 227.1565 | 203.79506 | 161.36055 | 218.3074 | 194.89994 | 57.241778 | 177.1269 | 25.72086 |
| | 1.00E-006 | 157.54953 | 189.82947 | 87.966976 | 123.37981 | 128.45388 | 15.394466 | 117.0957 | 24.68929 |
| | 1.00E-005 | 52.518633 | 42.82367 | 45.082433 | 51.882109 | 43.403997 | 12.904776 | 41.43594 | 5.958388 |
| | initial | 204.36588 | 216.38636 | 175.90323 | 236.21736 | 196.2519 | 118.48051 | 191.2675 | 16.71513 |
| **$H_2O_2$** | 1.00E-006 | | | 175.90323 | 236.21736 | 196.2519 | 122.95403 | 182.8316 | 23.56457 |
| | 3.00E-006 | | | 167.69764 | 230.45277 | 200.05704 | 135.64925 | 183.4642 | 20.44933 |
| | 1.00E-005 | 174.05124 | 210.70053 | 166.41321 | 202.50334 | 160.67561 | 117.85546 | 172.0332 | 13.56892 |
| | 3.00E-005 | 174.05124 | 172.75843 | 169.57065 | 179.06103 | 155.65791 | 108.54965 | 159.9415 | 10.77204 |
| | 1.00E-004 | 150.61789 | 144.25896 | 94.377567 | 172.02695 | 137.43766 | 70.908544 | 128.2713 | 15.48421 |
| | 3.00E-004 | 138.5762 | 137.39105 | 100.98989 | 189.10792 | 228.01402 | 128.62828 | 153.7846 | 18.86612 |
| | 1.00E-003 | 138.5762 | 131.7007 | 253.70275 | 248.08715 | 241.4279 | 203.0837 | 202.7631 | 22.59352 |
| | 3.00E-003 | 210.76892 | 235.39822 | 267.18741 | 260.1056 | 243.85564 | 220.89417 | 239.7017 | 8.941266 |
| | 1.00E-002 | 247.40202 | 236.66349 | 270.03542 | 265.16607 | 243.85564 | 226.09044 | 248.2022 | 6.846087 |
| | initial | 250.55341 | 250.05523 | 263.84026 | | 254.98473 | 212.98331 | 246.4834 | 8.732446 |
| **Norepinephrine** | 1.00E-009 | 250.55341 | 250.05523 | 264.47297 | | 254.98473 | 214.87236 | 246.9877 | 8.435657 |
| | 1.00E-008 | 250.55341 | 250.05523 | 264.47297 | | 254.98473 | 223.74997 | 248.7633 | 6.767717 |
| | 1.00E-007 | 255.61742 | 253.21707 | 264.47297 | | 254.98473 | 223.74997 | 250.4084 | 6.944239 |
| | 1.00E-006 | 255.61742 | 253.21707 | 264.47297 | | 248.11942 | 216.46035 | 247.5774 | 8.217277 |
| | 1.00E-005 | 255.61742 | 253.21707 | 241.06947 | | 248.75187 | 218.34683 | 243.4005 | 6.735635 |
| | without $Ca^{2+}$ | 279.66367 | 269.65935 | 274.85858 | 265.16607 | 250.01681 | 226.09044 | 260.9092 | 8.107592 |

## Figure 1. Panel A

| Date (number of experiments) | Concentration (M) | 20110603 | 20110602 | 20110607 | 20110627 | 20110629 | 20110922 | 20110922 | Average | ± SEM |
|---|---|---|---|---|---|---|---|---|---|---|
| | | Diameter (μm) | | | | | | | Diameter (μm) | |
| | before incubation | 289.9250522 | 199.5165487 | 202.5758187 | 171.6375765 | 237.3960979 | | 112.0427851 | 202.1823 | 24.49774 |
| | initial | 191.8658385 | 169.843471 | 120.3299514 | 76.59562062 | 94.18308252 | 48.12465423 | 96.216 | 113.8798 | 19.31517 |
| **5HT** | 1.00E-09 | 193.7641802 | 169.843471 | 110.8039408 | 76.59562062 | 81.73547934 | 48.12465423 | 96.216 | 111.0119 | 19.8353 |
| | 1.00E-08 | 193.7641802 | 169.843471 | 110.8039408 | 66.465 | 75.46517124 | 43.69534434 | 93.684 | 107.6744 | 20.88001 |
| | 1.00E-07 | 173.1437177 | 136.2421345 | 89.30909646 | 59.502 | 59.84444667 | 36.73581951 | 89.88822816 | 92.09506 | 18.05912 |
| | 0.000001 | 122.1755579 | 112.2571568 | 67.92004545 | 51.906 | 39.61690002 | 34.20543999 | 89.25524082 | 73.90519 | 13.19097 |
| | 0.00001 | 108.243 | 86.54293077 | 49.374 | 41.78279814 | 24.10392471 | 34.20543999 | 80.40096975 | 60.66472 | 11.79557 |
| | initial | 170.2781774 | 149.9782598 | 114.5747471 | 76.59562062 | 120.43646 | 53.89800669 | 89.26198227 | 110.7176 | 15.46987 |
| **Acetylcholine** | 1.00E-09 | 173.442 | 149.5006107 | 114.5747471 | 76.59562062 | 120.43646 | 53.89800669 | 89.26198227 | 111.1013 | 15.73584 |
| | 1.00E-08 | 197.6187071 | 214.4982914 | 131.1288175 | 81.024 | 120.43646 | 58.2256821 | 100.0320215 | 128.9949 | 21.95569 |
| | 1.00E-07 | 265.8871241 | 232.3247931 | 177.3123266 | 120.0131919 | 141.153322 | 82.90367451 | 108.8833618 | 161.2111 | 25.46861 |
| | 0.000001 | 278.5660318 | 240.6099465 | 199.4110719 | 156.2843514 | 181.1541555 | 96.46762383 | 116.5149934 | 181.2869 | 24.55596 |
| | 0.00001 | 286.7937215 | 240.6099465 | 200.0440339 | 169.843471 | 223.0900763 | 97.55595972 | 117.1323709 | 190.7242 | 25.53449 |
| | initial | 180.4594 | 161.4348572 | 117.7448111 | 79.78060443 | 93.07037613 | 48.17458527 | 93.15643248 | 110.5459 | 17.57561 |
| **$H_2O_2$** | 1.00E-06 | 180.4594 | 161.4348572 | 125.3355952 | 86.09731143 | 97.50049626 | 48.17458527 | 93.15643248 | 113.1655 | 17.32654 |
| | 3.00E-06 | 180.4594 | 183.0256896 | 125.3355952 | 86.09731143 | 126.6 | 48.17458527 | 93.15643248 | 120.407 | 18.72565 |
| | 1.00E-05 | 191.8157112 | 177.8775006 | 136.7411854 | 85.47609789 | 124.0744629 | 49.43887617 | 93.18869649 | 122.6589 | 19.29983 |
| | 3.00E-05 | 195.1653067 | 181.0424247 | 146.857361 | 86.7302418 | 124.0744629 | 52.37094483 | 90.6605388 | 125.2716 | 19.79704 |
| | 1.00E-04 | 195.1653067 | 181.0424247 | 164.58 | 91.16079237 | 124.0744629 | 49.63301094 | 90.55441002 | 128.0301 | 20.45836 |
| | 3.00E-04 | 194.9732545 | 181.0391014 | 184.8750245 | 117.1888029 | 124.0744629 | 47.3566923 | 113.3087661 | 137.5452 | 19.96844 |
| | 1.00E-03 | 201.4143903 | 221.5509052 | 201.9309626 | 162.6822344 | 175.7370301 | 89.30909646 | 128.8042263 | 168.7755 | 17.55809 |
| | 3.00E-03 | 223.2911361 | 246.2443238 | 201.9359253 | 172.1806526 | 176.5332428 | 96.2347368 | 128.8042263 | 177.8892 | 19.77445 |
| | 1.00E-02 | 276.8063551 | 258.2709757 | 205.7600619 | 179.1568886 | 245.0527709 | 102.5772575 | 129.6908694 | 199.6165 | 24.98839 |
| | initial | 292.8553801 | 243.7123998 | 208.3531844 | 165.9667574 | 235.0101563 | 103.8428714 | 121.6694491 | 195.9157 | 25.9357 |
| **5HT** | 1.00E-09 | 292.8553801 | 239.287388 | 212.3042754 | 165.9667574 | 235.0101563 | 103.8428714 | 121.6694491 | 195.848 | 25.80245 |
| | 1.00E-08 | 292.8553801 | 245.6561972 | 212.3042754 | 165.9667574 | 235.0101563 | 95.02592835 | 121.6694491 | 195.4983 | 26.83963 |
| | 1.00E-07 | 292.8553801 | 238.0618556 | 212.3042754 | 165.9667574 | 235.0101563 | 95.02592835 | 121.6694491 | 194.4134 | 26.52176 |
| | 0.000001 | 292.8553801 | 222.8169052 | 212.3042754 | 169.1294913 | 235.0101563 | 95.02592835 | 121.6694491 | 192.6874 | 25.93662 |
| | 0.00001 | 292.8553801 | 222.8169052 | 212.3042754 | 169.1294913 | 235.0101563 | 95.02592835 | 121.6694491 | 192.6874 | 25.93662 |
| | without $Ca^{2+}$ | 300.8128864 | 258.2709757 | 215.2209242 | 180.0225667 | 245.0527709 | 102.5772575 | 129.6908694 | 204.5212 | 26.95267 |

## Figure 1. Panel B / 10 µM

| Date (number of experiments) | | *20101015* | *20101020* | *20101029* | *20101028* | *20101026* | *20101025* | *Average* | *± SEM* |
|---|---|---|---|---|---|---|---|---|---|
| | **Concentration (M)** | **Diameter (µm)** | | | | | | **Diameter (µm)** | |
| | before incubation | 234.34916 | 257.66472 | 242.60478 | 229.729 | 261.99029 | 266.41842 | 248.79273 | 6.2553604 |
| | initial | 105.18388 | 227.79709 | 196.44559 | 184.11387 | 210.6074 | 247.50557 | 195.27557 | 20.218104 |
| **Achetylcholine** | 1.00E-009 | 112.13411 | 227.16443 | 196.44559 | 184.11387 | 210.6074 | 247.50557 | 196.3285 | 19.158758 |
| | 1.00E-008 | 169.05165 | 227.79709 | 196.44559 | 197.76575 | 212.86203 | 241.85697 | 207.62985 | 10.513888 |
| | 1.00E-007 | 200.10505 | 227.79709 | 203.5474 | 227.53068 | 234.37907 | 264.11323 | 226.24542 | 9.4915846 |
| | 1.00E-006 | 213.96875 | 251.82518 | 210.55133 | 230.55698 | 248.75187 | 264.05866 | 236.6188 | 8.8706903 |
| | 1.00E-005 | 221.60072 | 252.02144 | 235.70837 | 233.71713 | 252.58471 | 270.2266 | 244.30983 | 7.0746401 |
| | initial | 141.74933 | 229.77867 | 187.90062 | 233.77878 | 217.35178 | 228.49544 | 206.5091 | 14.635771 |
| **Norepinephrine** | 1.00E-009 | 141.74933 | 229.77867 | 187.90062 | 226.79583 | 214.89937 | 228.49544 | 204.93654 | 14.179899 |
| | 1.00E-008 | 138.58631 | 222.12489 | 187.90062 | 226.79583 | 214.86118 | 228.49544 | 203.12738 | 14.25918 |
| | 1.00E-007 | 117.05281 | 215.7991 | 187.90062 | 195.46499 | 214.86118 | 211.4847 | 190.42723 | 15.383688 |
| | 1.00E-006 | 55.136484 | 215.7991 | 158.76953 | 171.09476 | 162.19199 | 211.4847 | 162.41276 | 23.703063 |
| | 1.00E-005 | 35.572633 | 55.692742 | 69.936488 | 139.66103 | 67.655466 | 86.085585 | 75.767324 | 14.499742 |
| | **Time (sec)** | **Diameter (µm)** | | | | | | **Diameter (µm)** | |
| **$H_2O_2$ 10 µM** | initial | 136.78365 | 217.65546 | 198.92122 | 153.85362 | 219.70393 | 235.77885 | 193.78279 | 16.201979 |
| | 0 | 136.78365 | 217.65546 | 198.1833 | 153.8315 | 219.70393 | 235.77885 | 193.65611 | 16.196455 |
| | 10 | 140.5767 | 215.75736 | 196.93914 | 150.64978 | 208.19577 | 232.58389 | 190.78377 | 15.103514 |
| | 20 | 142.49998 | 213.22656 | 196.93914 | 147.48763 | 203.76756 | 230.6889 | 189.10163 | 14.709938 |
| | 30 | 142.49998 | 213.33637 | 196.93914 | 147.48763 | 201.23717 | 230.6889 | 188.6982 | 14.637832 |
| | 40 | 142.49998 | 210.17454 | 194.45165 | 146.22278 | 183.61746 | 226.89906 | 183.97758 | 13.885468 |
| | 50 | 142.49998 | 210.76892 | 194.45165 | 146.22278 | 167.11176 | 223.74101 | 180.79935 | 13.878374 |
| | 60 | 143.13206 | 208.23902 | 191.9653 | 146.22278 | 164.85551 | 223.74101 | 179.69262 | 13.641628 |
| | 70 | 145.02836 | 209.43038 | 187.61706 | 146.22278 | 157.75902 | 223.74101 | 178.29977 | 13.758241 |
| | 80 | 145.02836 | 208.16498 | 189.97809 | 146.22278 | 150.7454 | 220.58315 | 176.78713 | 13.781187 |
| | 90 | 146.29258 | 206.2669 | 190.59763 | 146.22278 | 146.9247 | 220.22715 | 176.08862 | 13.783753 |
| | 100 | 146.29258 | 206.2669 | 182.79258 | 146.22278 | 141.16352 | 220.22715 | 173.82759 | 13.991357 |
| | 110 | 146.29258 | 203.73218 | 182.79258 | 142.42832 | 134.20734 | 219.04882 | 171.41697 | 14.489771 |
| | 120 | 146.29258 | 202.46775 | 187.79833 | 142.41006 | 133.51502 | 218.72785 | 171.8686 | 14.581711 |
| | 130 | 146.32131 | 205.00639 | 187.16737 | 142.41006 | 132.88237 | 218.72785 | 172.08589 | 14.796472 |
| | 140 | 146.32131 | 201.84298 | 187.16737 | 141.14509 | 132.88237 | 218.72785 | 171.34783 | 14.655149 |
| | 150 | 146.32131 | 201.84298 | 187.16737 | 141.77757 | 123.41874 | 218.72785 | 169.87597 | 15.502693 |
| | 160 | 146.32131 | 201.84298 | 182.12011 | 141.77757 | 123.41874 | 218.72785 | 169.03476 | 15.336977 |
| | 170 | 146.67518 | 208.16979 | 179.59677 | 143.64715 | 125.40566 | 219.72853 | 170.53718 | 15.542845 |
| | 180 | 146.67518 | 210.06784 | 179.59677 | 143.64715 | 125.40566 | 220.51053 | 170.98385 | 15.779956 |
| | 190 | 149.4318 | 211.3332 | 175.46118 | 143.64715 | 125.40566 | 220.44517 | 170.95403 | 15.688309 |
| | 200 | 149.4318 | 212.59858 | 175.46118 | 139.21891 | 124.14157 | 223.63632 | 170.74806 | 16.524208 |
| | 210 | 149.4318 | 212.59858 | 171.68337 | 139.21891 | 124.14157 | 223.63632 | 170.11843 | 16.500269 |
| | 220 | 149.4318 | 214.49663 | 171.68337 | 139.21891 | 124.14157 | 223.63632 | 170.43477 | 16.665361 |
| | 230 | 149.4318 | 214.50316 | 171.68337 | 139.21891 | 121.0476 | 228.69158 | 170.76274 | 17.503752 |

| | | | | | | | | | | |
|---|---|---|---|---|---|---|---|---|---|---|
| | 240 | 149.4318 | 214.50316 | 170.42427 | 140.48412 | 120.41595 | 228.69158 | | 170.65848 | 17.48869 |
| | 250 | 149.4318 | 214.51156 | 158.90437 | 140.48412 | 120.41595 | 231.85124 | | 169.2665 | 17.962514 |
| | 260 | 150.14537 | 214.51156 | 158.90437 | 140.48412 | 118.52105 | 231.85124 | | 169.06962 | 18.111001 |
| | 270 | 150.14537 | 215.77684 | 167.84319 | 140.48412 | 118.52105 | 231.85124 | | 170.7703 | 18.108619 |
| | 280 | 150.14537 | 215.77684 | 167.84319 | 140.48412 | 137.19278 | 229.4256 | | 173.47798 | 16.22827 |
| | 290 | 150.14537 | 219.72853 | 167.84319 | 140.48412 | 144.7576 | 224.45111 | | 174.56832 | 15.513281 |
| | 300 | 150.14537 | 215.30324 | 167.84319 | 141.11672 | 160.49987 | 224.45111 | | 176.55992 | 14.238991 |
| | 310 | 150.14537 | 215.30324 | 167.84319 | 141.11672 | 172.04557 | 229.58608 | | 179.34003 | 14.510632 |
| | 320 | 150.23467 | 218.46413 | 167.84319 | 141.74933 | 172.04557 | 229.58608 | | 179.98716 | 14.717809 |
| | 340 | 151.48715 | 217.67477 | 173.97186 | 136.0559 | 172.04557 | 228.95487 | | 180.03169 | 14.899263 |
| | 350 | 153.8419 | 217.67477 | 171.45002 | 136.06914 | 176.51778 | 228.95487 | | 180.75142 | 14.733154 |
| | 360 | 153.8419 | 221.46249 | 171.45002 | 136.06914 | 176.51778 | 228.95487 | | 181.3827 | 15.059483 |
| | 370 | 153.8419 | 221.46249 | 171.45002 | 136.06914 | 178.18248 | 228.95487 | | 181.66015 | 15.044105 |
| | 380 | 153.8419 | 221.46249 | 177.12451 | 136.06914 | 180.68124 | 228.91203 | | 183.01522 | 14.921879 |
| | 390 | 153.7417 | 221.46249 | 177.12451 | 138.5993 | 181.20339 | 228.91203 | | 183.50724 | 14.663962 |
| | 400 | 153.7417 | 221.46249 | 177.12451 | 138.5993 | 183.8386 | 227.64941 | | 183.736 | 14.528567 |
| | 410 | 155.71575 | 221.46249 | 177.12451 | 140.49694 | 183.8386 | 228.20004 | | 184.47306 | 14.2543 |
| | 420 | 155.71575 | 222.72783 | 173.97186 | 141.76204 | 185.10161 | 223.77948 | | 183.8431 | 13.865936 |
| | 430 | 156.3405 | 222.72783 | 175.86342 | 144.29226 | 187.35015 | 223.74101 | | 185.05253 | 13.529876 |
| | 440 | 156.3405 | 222.72783 | 175.86342 | 144.29226 | 187.35015 | 223.74101 | | 185.05253 | 13.529876 |
| | 450 | 156.3405 | 220.21534 | 177.12451 | 144.29226 | 189.52238 | 223.74101 | | 185.206 | 13.293725 |
| | 460 | 156.3405 | 221.49232 | 177.12451 | 144.29226 | 193.94352 | 223.74101 | | 186.15569 | 13.472242 |
| | 470 | 156.3405 | 221.49232 | 177.7551 | 144.29226 | 193.94352 | 223.74101 | | 186.26079 | 13.458554 |
| | 480 | 156.3405 | 221.49232 | 181.64469 | 144.29226 | 194.61319 | 223.74101 | | 187.02066 | 13.404248 |
| | 490 | 156.3405 | 221.49232 | 181.64469 | 144.29226 | 194.73966 | 223.74101 | | 187.04174 | 13.406652 |
| | 500 | 156.44162 | 221.49232 | 185.90547 | 144.29226 | 195.37077 | 223.74101 | | 187.87391 | 13.371704 |
| | 510 | 156.54522 | 220.82982 | 185.90547 | 144.29226 | 195.37077 | 223.74101 | | 187.78076 | 13.308411 |
| | 520 | 156.54522 | 221.46249 | 189.06028 | 144.29226 | 196.58921 | 223.74101 | | 188.61508 | 13.378537 |
| | 530 | 155.92128 | 222.72783 | 189.06028 | 144.29226 | 197.22047 | 223.74101 | | 188.82719 | 13.546081 |
| | 540 | 155.92128 | 221.44893 | 191.58434 | 144.29226 | 199.11433 | 223.74101 | | 189.35036 | 13.492093 |
| | 550 | 155.92128 | 221.44893 | 193.52406 | 144.29226 | 202.90227 | 223.74101 | | 190.30497 | 13.609283 |
| | **Concentration (M)** | | | **Diameter (µm)** | | | | | **Diameter (µm)** | |
| | initial | 134.80406 | 205.01322 | 213.61297 | 162.70579 | 227.23051 | 224.36905 | | 194.6226 | 15.290156 |
| **Norepinephrine** | 1.00E-009 | 135.43661 | 202.48258 | 189.6459 | 162.70579 | 224.0682 | 219.34559 | | 188.94744 | 14.015749 |
| | 1.00E-008 | 114.58318 | 201.21726 | 189.6459 | 159.54423 | 224.0682 | 219.97342 | | 184.8387 | 16.970973 |
| | 1.00E-007 | 85.425044 | 198.68664 | 174.02592 | 150.06003 | 224.0682 | 217.46224 | | 174.95468 | 21.148994 |
| | 1.00E-006 | 83.527127 | 85.941309 | 150.80913 | 102.81272 | 175.98516 | 193.91977 | | 132.16587 | 19.532273 |
| | 1.00E-005 | 34.798975 | 50.84553 | 72.19813 | 71.675026 | 89.775542 | 90.539259 | | 68.305411 | 8.9616717 |
| | without $Ca^{2+}$ | | 254.03153 | 223.34704 | 223.41874 | 222.72783 | 245.67433 | | 233.83989 | 6.6705876 |

## Figure 1. Panel B/30 μM

| Date (number of experiments) | | 20101015 | 20101025 | 20101013 | 20101014 | Average | ± SEM |
|---|---|---|---|---|---|---|---|
| | **Concentration (M)** | **Diameter (μm)** | | | | **Diameter (μm)** | |
| | before incubation | 234.3491576 | 266.4184192 | 230.9603195 | 217.651774 | 237.3449 | 10.33935 |
| | initial | 105.1838832 | 247.5055683 | 143.0047511 | 139.8286106 | 158.8807 | 30.75812 |
| **Achetylcholine** | 1.00E-009 | 112.1341117 | 247.5055683 | 143.6374028 | 146.1556971 | 162.3582 | 29.41871 |
| | 1.00E-008 | 169.051645 | 241.8569733 | 157.5495333 | 155.0962372 | 180.8886 | 20.54916 |
| | 1.00E-007 | 200.1050549 | 264.1132275 | 198.6745513 | 170.2738736 | 208.2917 | 19.83454 |
| | 1.00E-006 | 213.9687517 | 264.0586564 | 232.2738922 | 183.6490687 | 223.4876 | 16.8348 |
| | 1.00E-005 | 221.6007174 | 270.226604 | 238.5991249 | 213.462996 | 235.9724 | 12.56141 |
| | initial | 134.8040625 | 224.3690519 | 153.7482008 | 117.7263261 | 157.6619 | 23.42095 |
| **Norepinephrine** | 1.00E-009 | 135.4366066 | 219.345592 | 155.0136181 | 122.1275233 | 157.9808 | 21.54093 |
| | 1.00E-008 | 114.583175 | 219.9734161 | 153.7482008 | 120.8738554 | 152.2947 | 24.1384 |
| | 1.00E-007 | 85.42504386 | 217.4622398 | 144.9027125 | 107.6274419 | 138.8544 | 28.93325 |
| | 1.00E-006 | 83.52712672 | 193.9197712 | 42.56112108 | 49.99999684 | 92.502 | 34.96024 |
| | 1.00E-005 | 34.79897549 | 90.53925948 | 36.89293741 | 22.77751123 | 46.25217 | 15.08635 |
| | **Time (sec)** | **Diameter (μm)** | | | | **Diameter (μm)** | |
| **$H_2O_2$ 30 μM** | initial | 151.5716894 | 231.3084548 | 128.4788109 | 157.5444527 | 167.2259 | 22.26113 |
| | 0 | 151.5716894 | 231.3084548 | 128.439855 | 157.5444527 | 167.2161 | 22.26678 |
| | 10 | 147.9307485 | 229.6043806 | 131.068462 | 145.5724979 | 163.544 | 22.33343 |
| | 20 | 142.1174025 | 220.6638784 | 136.6782467 | 139.2878978 | 159.6869 | 20.35599 |
| | 30 | 123.7151728 | 201.0231848 | 137.9435501 | 139.2878978 | 150.4925 | 17.20803 |
| | 40 | 119.4127603 | 180.2775541 | 139.8414926 | 153.3036154 | 148.2089 | 12.75926 |
| | 50 | 89.14234002 | 160.5260479 | 142.3720994 | 164.0473246 | 139.022 | 17.29136 |
| | 60 | 81.8740615 | 158.1328654 | 142.3720994 | 176.6390745 | 139.7545 | 20.52485 |
| | 70 | 79.9969921 | 155.2974259 | 143.0047511 | 181.0652511 | 139.8411 | 21.46636 |
| | 80 | 79.9969921 | 155.2974259 | 143.0047511 | 187.3886109 | 141.4219 | 22.5111 |
| | 90 | 79.37152797 | 155.2974259 | 143.0047511 | 190.5503825 | 142.056 | 23.19713 |
| | 100 | 69.61520495 | 153.841905 | 144.9027125 | 193.7122048 | 140.518 | 25.90621 |
| | 110 | 70.24205471 | 153.841905 | 146.8224961 | 198.1196532 | 142.2565 | 26.55473 |
| | 120 | 70.24205471 | 153.841905 | 146.1899329 | 200.6494691 | 142.7308 | 26.99477 |
| | 130 | 70.24205471 | 149.8932019 | 146.1899329 | 201.9143612 | 142.0599 | 27.109 |
| | 140 | 70.24205471 | 148.0186697 | 146.1899329 | 201.9143612 | 141.5913 | 27.06789 |
| | 150 | 70.24205471 | 145.2269694 | 146.8224961 | 205.6925999 | 141.996 | 27.7483 |

| | | | | | | |
|---|---|---|---|---|---|---|
| 160 | 85.12224216 | 145.2269694 | 146.8224961 | 206.9576312 | 146.0323 | 24.87168 |
| 170 | 85.12224216 | 145.2269694 | 146.8224961 | 205.6925999 | 145.7161 | 24.61412 |
| 180 | 88.27363105 | 145.2269694 | 148.075449 | 206.9576312 | 147.1334 | 24.23485 |
| 190 | 89.57018393 | 145.2269694 | 149.3406828 | 206.9576312 | 147.7739 | 23.97799 |
| 200 | 86.34791902 | 145.2269694 | 149.3406828 | 208.2226816 | 147.2846 | 24.89175 |
| 210 | 86.34791902 | 145.8527245 | 149.9733029 | 209.4877129 | 147.9154 | 25.14987 |
| 220 | 89.66622278 | 145.8527245 | 149.9733029 | 209.4877129 | 148.745 | 24.47763 |
| 230 | 89.66622278 | 155.0910743 | 148.075449 | 209.4877129 | 150.5801 | 24.50719 |
| 240 | 89.66622278 | 155.0910743 | 149.3406828 | 210.1202444 | 151.0546 | 24.62471 |
| 250 | 91.33823763 | 155.0910743 | 148.075449 | 210.1202444 | 151.1563 | 24.28978 |
| 260 | 93.82879398 | 155.0910743 | 148.075449 | 210.136448 | 151.7829 | 23.78463 |
| 270 | 98.81604395 | 158.2151238 | 148.075449 | 212.6663525 | 154.4432 | 23.34361 |
| 280 | 100.7577319 | 161.3407046 | 147.4428352 | 212.6663525 | 155.5519 | 23.02848 |
| 290 | 103.8586939 | 165.814328 | 147.4428352 | 212.6663525 | 157.4456 | 22.52957 |
| 300 | 103.8586939 | 170.587444 | 147.4428352 | 213.9313269 | 158.9551 | 22.95983 |
| 310 | 105.9706753 | 170.587444 | 149.3406828 | 213.9490934 | 159.962 | 22.46364 |
| 320 | 105.9706753 | 170.587444 | 149.3406828 | 213.9490934 | 159.962 | 22.46364 |
| 340 | 105.3512156 | 171.8360249 | 149.3406828 | 213.9490934 | 160.1193 | 22.63966 |
| 350 | 105.3512156 | 177.7956279 | 149.3406828 | 213.9490934 | 161.6092 | 22.94367 |
| 360 | 105.3512156 | 177.7956279 | 153.1364032 | 213.9490934 | 162.5581 | 22.79366 |
| 370 | 110.3110756 | 180.7820317 | 153.1364032 | 213.9490934 | 164.5447 | 21.93908 |
| 380 | 110.3110756 | 180.7820317 | 153.1364032 | 217.1112826 | 165.3352 | 22.53855 |
| 390 | 110.3110756 | 180.7820317 | 149.9733029 | 217.1112826 | 164.5444 | 22.69454 |
| 400 | 110.3110756 | 180.7820317 | 149.9733029 | 217.1112826 | 164.5444 | 22.69454 |
| 410 | 110.3110756 | 184.6837498 | 149.9733029 | 217.1306309 | 165.5247 | 22.95037 |
| 420 | 110.3110756 | 185.1372943 | 149.9733029 | 217.1306309 | 165.6381 | 22.98218 |
| 430 | 112.4478276 | 207.1819011 | 149.9733029 | 216.4982512 | 171.5253 | 24.57742 |
| 440 | 111.8248817 | 206.5539441 | 149.9733029 | 216.4982512 | 171.2126 | 24.6269 |
| 450 | 112.0537577 | 207.1055522 | 149.3406828 | 205.0220047 | 168.3805 | 23.05328 |
| 460 | 113.9191853 | 210.2468937 | 149.3406828 | 205.0220047 | 169.6322 | 23.12588 |
| 470 | 113.9191853 | 216.3234465 | 149.3406828 | 205.0220047 | 171.1513 | 24.04677 |
| 480 | 113.9191853 | 223.2457662 | 147.4428352 | 205.0327417 | 172.4101 | 25.31971 |
| 490 | 115.1634005 | 223.2457662 | 147.4428352 | 205.6653555 | 172.8793 | 25.1493 |
| 500 | 115.1634005 | 223.2457662 | 147.4428352 | 205.6925999 | 172.8862 | 25.15226 |

| | | | | | | | |
|---|---|---|---|---|---|---|---|
| | 510 | 114.5412328 | 223.2457662 | 147.4428352 | 205.6925999 | 172.7306 | 25.27144 |
| | 520 | 114.5412328 | 224.6899807 | 151.2385493 | 205.6925999 | 174.0406 | 25.2113 |
| | 530 | 114.5412328 | 224.6899807 | 153.1364032 | 205.6925999 | 174.5151 | 25.07234 |
| | 540 | 119.9312587 | 232.2006182 | 153.1364032 | 205.6925999 | 177.7402 | 25.32208 |
| | 550 | 119.9312587 | 239.6162167 | 153.1364032 | 205.6925999 | 179.5941 | 26.68247 |
| | 560 | 121.0790049 | 242.7656252 | 155.6669024 | 205.6925999 | 181.301 | 26.85865 |
| | 570 | 121.0790049 | 245.9153817 | 155.6669024 | 205.6925999 | 182.0885 | 27.46404 |
| | 580 | 121.0790049 | 246.5453823 | 155.6669024 | 205.6925999 | 182.246 | 27.58623 |
| | 590 | 121.7039375 | 249.6955691 | 151.8500749 | 205.6925999 | 182.2355 | 28.41426 |
| | 600 | 121.7039375 | 254.1063772 | 151.8500749 | 205.6925999 | 183.3382 | 29.29468 |
| | 610 | 121.7039375 | 254.1063772 | 151.8500749 | 205.6925999 | 183.3382 | 29.29468 |
| | 620 | 147.860366 | 254.1063772 | 150.5846576 | 205.6925999 | 189.561 | 25.30552 |
| | 630 | 113.6518089 | 259.1480591 | 150.5846576 | 205.6925999 | 182.2693 | 31.84765 |
| | 640 | 113.6518089 | 264.7672142 | 150.5846576 | 207.5901437 | 184.1485 | 33.09733 |
| | 650 | 113.6518089 | 264.7672142 | 150.5846576 | 207.5901437 | 184.1485 | 33.09733 |
| | 660 | 113.6518089 | 264.7672142 | 150.5846576 | 207.5901437 | 184.1485 | 33.09733 |
| | 670 | 122.7666793 | 264.7672142 | 150.5846576 | 207.5901437 | 186.4272 | 31.52033 |
| | 680 | 122.7666793 | 264.7672142 | 148.053823 | 207.5901437 | 185.7945 | 31.76555 |
| | initial | 110.180358 | 216.5943913 | 156.6512959 | 143.0383416 | 156.6161 | 22.24491 |
| | **Concentration (M)** | **Diameter (µm)** | | | | **Diameter (µm)** | |
| **Norepinephrine** | 1.00E-009 | 115.1529734 | 216.5943913 | 105.0315396 | 143.0383416 | 144.9543 | 25.19577 |
| | 1.00E-008 | 115.1529734 | 212.8310023 | 105.0315396 | 138.0668207 | 142.7706 | 24.35425 |
| | 1.00E-007 | 82.9234594 | 212.8310023 | 88.58146912 | 90.73139411 | 118.7668 | 31.39793 |
| | 1.00E-006 | 41.75877059 | 153.9927554 | 35.48812808 | 41.24755466 | 68.1218 | 28.65893 |
| | 1.00E-005 | 28.47188904 | 78.26173803 | 25.59148293 | 36.3518513 | 42.16924 | 12.24388 |
| | without $Ca^{2+}$ | 234.3491576 | 266.4184192 | 230.9603195 | 212.7745584 | 236.1256 | 11.15333 |

## Figure 1. Panel B/100 µM

| Date (number of experiments) | | 20101020 | 20101029 | 20101028 | 20101026 | 20101025 | Average | ± SEM |
|---|---|---|---|---|---|---|---|---|
| | **Concentration (M)** | **Diameter (µm)** | | | | | **Diameter (µm)** | |
| | before incubation | 257.664724 | 242.604784 | 229.728999 | 261.990288 | 266.418419 | 251.6814 | 6.795388 |
| | initial | 227.797086 | 196.445588 | 184.113869 | 210.607405 | 228.495445 | 209.4919 | 8.693991 |
| **Achetylcholine** | 1.00E-009 | 227.164435 | 196.445588 | 184.113869 | 210.607405 | 228.495445 | 209.3653 | 8.628059 |
| | 1.00E-008 | 227.797086 | 196.445588 | 197.765748 | 212.86203 | 228.495445 | 212.6732 | 6.945065 |
| | 1.00E-007 | 227.797086 | 203.547401 | 227.530684 | 234.379066 | 211.4847 | 220.9478 | 5.757474 |
| | 1.00E-006 | 251.825184 | 210.551334 | 230.55698 | 248.751871 | 211.4847 | 230.634 | 8.795847 |
| | 1.00E-005 | 252.021438 | 235.708374 | 233.717126 | 252.584713 | 86.0855854 | 212.0234 | 31.73089 |
| | initial | 229.778666 | 187.900624 | 233.778777 | 217.351775 | 247.505568 | 223.2631 | 10.06523 |
| **Norepinephrine** | 1.00E-009 | 229.778666 | 187.900624 | 226.795831 | 214.899371 | 247.505568 | 221.376 | 9.862254 |
| | 1.00E-008 | 222.124891 | 187.900624 | 226.795831 | 214.861181 | 241.856973 | 218.7079 | 8.879346 |
| | 1.00E-007 | 215.799102 | 187.900624 | 195.464991 | 214.861181 | 264.113228 | 215.6278 | 13.27929 |
| | 1.00E-006 | 215.799102 | 158.769535 | 171.094762 | 162.191989 | 264.058656 | 194.3828 | 20.19934 |
| | 1.00E-005 | 55.6927423 | 69.9364881 | 139.661031 | 67.6554658 | 270.226604 | 120.6345 | 40.2084 |
| | **Time (sec)** | **Diameter (µm)** | | | | | **Diameter (µm)** | |
| **$H_2O_2$ 100 µM** | initial | 191.000485 | 184.145384 | 168.991253 | 206.775721 | 231.308455 | 196.4443 | 10.62265 |
| | 0 | 191.000485 | 184.145384 | 168.991253 | 206.775721 | 231.308455 | 196.4443 | 10.62265 |
| | 10 | 189.561446 | 163.243734 | 163.931374 | 201.773565 | 229.604381 | 189.6229 | 12.45407 |
| | 20 | 189.561446 | 163.243734 | 163.931374 | 199.880861 | 220.663878 | 187.4563 | 10.95722 |
| | 30 | 187.334122 | 138.563193 | 163.931374 | 199.249987 | 201.023185 | 178.0204 | 11.87843 |
| | 40 | 183.669771 | 136.670926 | 163.931374 | 185.141616 | 180.277554 | 169.9382 | 9.13618 |
| | 50 | 176.662864 | 149.706142 | 161.351866 | 165.890367 | 160.526048 | 162.8275 | 4.361437 |
| | 60 | 169.831294 | 147.507985 | 156.382743 | 139.402817 | 158.132865 | 154.2515 | 5.139531 |
| | 70 | 163.680885 | 117.078458 | 156.382743 | 122.642694 | 155.297426 | 143.0164 | 9.603042 |
| | 80 | 163.680885 | 99.3674938 | 149.427781 | 118.521046 | 155.297426 | 137.2589 | 12.16235 |
| | 90 | 150.71752 | 98.71066 | 149.427781 | 123.02075 | 155.297426 | 135.4348 | 10.78578 |
| | 100 | 144.944149 | 98.71066 | 149.427781 | 117.435229 | 153.841905 | 132.8719 | 10.64157 |
| | 110 | 144.944149 | 96.1800342 | 149.427781 | 115.994672 | 153.841905 | 132.0777 | 11.15248 |
| | 120 | 140.616561 | 96.1800342 | 146.898813 | 113.579573 | 153.841905 | 130.2234 | 10.90846 |
| | 130 | 139.381273 | 96.1800342 | 148.720185 | 113.579573 | 149.893202 | 129.5509 | 10.5986 |
| | 140 | 134.445753 | 94.9400392 | 148.087616 | 135.352336 | 148.01867 | 132.1689 | 9.761985 |
| | 150 | 131.51363 | 80.9965958 | 164.110773 | 135.352336 | 145.226969 | 131.4401 | 13.81541 |
| | 160 | 131.51363 | 68.9768525 | 175.316252 | 135.352336 | 145.226969 | 131.2772 | 17.36668 |
| | 170 | 130.135116 | 68.9768525 | 184.778017 | 136.3733 | 145.226969 | 133.0981 | 18.63996 |
| | 180 | 129.845626 | 68.9768525 | 184.778017 | 172.811712 | 145.226969 | 140.3278 | 20.31731 |
| | 190 | 133.829476 | 68.9768525 | 187.941092 | 172.811712 | 145.226969 | 141.7572 | 20.57815 |

| | | | | | | | |
|---|---|---|---|---|---|---|---|
| 200 | 137.345875 | 68.9768525 | 183.290139 | 157.60034 | 145.226969 | 138.488 | 19.04079 |
| 210 | 139.339628 | 68.9768525 | 183.489873 | 157.60034 | 145.852725 | 139.0519 | 19.07305 |
| 220 | 151.005434 | 72.8054928 | 183.489873 | 159.97022 | 145.852725 | 142.6247 | 18.60942 |
| 230 | 165.939832 | 72.8054928 | 183.699198 | 166.041129 | 155.091074 | 148.7153 | 19.52395 |
| 240 | 177.073657 | 74.7025051 | 183.699198 | 167.452771 | 155.091074 | 151.6038 | 19.81917 |
| 250 | 180.299756 | 74.7025051 | 178.464211 | 169.003104 | 155.091074 | 151.5121 | 19.71497 |
| 260 | 193.116105 | 74.7025051 | 178.464211 | 179.261006 | 155.091074 | 156.127 | 21.25247 |
| 270 | 198.066113 | 74.7025051 | 178.464211 | 179.261006 | 158.215124 | 157.7418 | 21.69596 |
| 280 | 198.066113 | 74.7025051 | 179.729363 | 184.893885 | 161.340705 | 159.7465 | 22.06255 |
| 290 | 195.311338 | 75.3348531 | 186.687646 | 198.574779 | 165.814328 | 164.3446 | 22.97254 |
| 300 | 198.417475 | 75.9672075 | 186.687646 | 198.574779 | 170.587444 | 166.0469 | 23.09429 |
| 310 | 212.554325 | 77.2319351 | 186.687646 | 198.731957 | 170.587444 | 169.1587 | 23.99749 |
| 320 | 204.248303 | 77.8643084 | 187.952816 | 203.494279 | 170.587444 | 168.8294 | 23.55801 |
| 340 | 218.335827 | 78.4966817 | 188.153973 | 208.971125 | 171.836025 | 173.1587 | 25.01101 |
| 350 | 219.582725 | 78.4966817 | 193.209366 | 208.870531 | 177.795628 | 175.591 | 25.28376 |
| 360 | 219.582725 | 78.4966817 | 195.838403 | 218.66836 | 177.795628 | 178.0764 | 26.07889 |
| 370 | 228.526973 | 78.5196301 | 199.472871 | 221.347678 | 180.782032 | 181.7298 | 27.13633 |
| 380 | 237.22187 | 78.5196301 | 201.237167 | 217.969729 | 180.782032 | 183.1461 | 27.76369 |
| 390 | 244.146853 | 78.5196301 | 207.606537 | 217.880637 | 180.782032 | 185.7871 | 28.67342 |
| 400 | 244.146853 | 79.1113582 | 208.239018 | 217.880637 | 180.782032 | 186.032 | 28.58707 |
| 410 | 256.517902 | 78.478833 | 212.017833 | 217.010764 | 184.68375 | 189.7418 | 30.08646 |
| 420 | 256.517902 | 105.755124 | 215.813021 | 223.998519 | 185.137294 | 197.4444 | 25.58378 |
| 430 | 256.517902 | 109.090416 | 215.813021 | 223.998519 | 207.181901 | 202.5204 | 24.80386 |
| 440 | 252.104816 | 109.090416 | 217.078091 | 223.998519 | 206.553944 | 201.7652 | 24.3664 |
| 450 | 256.465615 | 114.140355 | 217.710629 | 223.998519 | 207.105552 | 203.8841 | 23.90073 |
| 460 | 256.465615 | 114.140355 | 221.740685 | 223.998519 | 210.246894 | 205.3184 | 24.05452 |
| 470 | 259.618788 | 124.243099 | 221.740685 | 223.998519 | 216.323446 | 209.1849 | 22.56849 |
| 480 | 259.618788 | 134.772876 | 224.410969 | 223.998519 | 223.245766 | 213.2094 | 20.79511 |
| 490 | 259.618788 | 133.507522 | 224.410969 | 219.181278 | 223.245766 | 211.9929 | 20.92927 |
| 500 | 259.618788 | 133.507522 | 226.936995 | 219.181278 | 223.245766 | 212.4981 | 21.01015 |
| 510 | 259.618788 | 132.874839 | 225.673975 | 219.728526 | 223.245766 | 212.2284 | 21.09651 |
| 520 | 260.104057 | 135.40556 | 225.042463 | 219.728526 | 224.689981 | 212.9941 | 20.69618 |
| 530 | 260.104057 | 152.547092 | 225.042463 | 219.728526 | 224.689981 | 216.4224 | 17.52389 |
| 540 | 261.320932 | 155.076876 | 225.042463 | 219.728526 | 232.200618 | 218.6739 | 17.44705 |
| 550 | 261.320932 | 155.8314 | 225.042463 | 219.728526 | 239.616217 | 220.3079 | 17.65623 |
| 560 | 261.320932 | 157.727299 | 225.042463 | 219.728526 | 242.765625 | 221.317 | 17.49344 |
| 570 | 265.323036 | 157.727299 | 225.042463 | 219.728526 | 245.915382 | 222.7473 | 18.15411 |
| 580 | 264.620508 | 160.887249 | 225.042463 | 219.728526 | 246.545382 | 223.3648 | 17.5483 |

| | | | | | | | | |
|---|---|---|---|---|---|---|---|---|
| | 590 | 265.252869 | 160.887249 | 225.042463 | 211.666338 | 249.695569 | 222.5089 | 18.01023 |
| | 600 | 265.252869 | 160.887249 | 225.042463 | 211.666338 | 254.106377 | 223.3911 | 18.36132 |
| | 610 | 265.252869 | 162.78328 | 225.042463 | 211.666338 | 254.106377 | 223.7703 | 18.03971 |
| | 620 | 265.252869 | 164.679356 | 225.042463 | 211.371084 | 254.106377 | 224.0904 | 17.73086 |
| | initial | 222.189769 | 165.446958 | 146.189933 | 211.439265 | 216.594391 | 192.3721 | 15.32502 |
| | **Concentration (M)** | | | **Diameter (μm)** | | | **Diameter (μm)** | |
| **Norepinephrine** | 1.00E-009 | 202.348096 | 165.446958 | 146.189933 | 208.909822 | 216.594391 | 187.8978 | 13.63341 |
| | 1.00E-008 | 202.348096 | 157.237962 | 143.027143 | 179.939707 | 212.831002 | 179.0768 | 13.14944 |
| | 1.00E-007 | 198.440664 | 152.777847 | 136.666535 | 179.939707 | 212.831002 | 176.1312 | 14.07468 |
| | 1.00E-006 | 142.920753 | 122.204524 | 87.3160835 | 150.532813 | 153.992755 | 131.3934 | 12.32309 |
| | 1.00E-005 | 71.9898997 | 56.9613484 | 49.35533 | 114.647781 | 78.261738 | 74.24322 | 11.33981 |
| | without $Ca^{2+}$ | 254.031534 | 223.347038 | 229.728999 | 222.727831 | 256.31026 | 237.2291 | 7.435262 |

# Figure 1. Panel B/300 μM

| Date (number of experiments) | | 20120301 | 20120301A | Average | ± SEM |
|---|---|---|---|---|---|
| | **Concentration (M)** | **Diameter (μm)** | | **Diameter (μm)** | |
| | before incubation | 175.597903 | 95.583 | 135.5905 | 40.00745 |
| | initial | 180.991519 | 97.482 | 139.2368 | 41.75476 |
| **Norepinephrine** | 1.00E-009 | 180.991519 | 96.85728 | 138.9244 | 42.06712 |
| | 1.00E-008 | 180.991519 | 96.85728 | 138.9244 | 42.06712 |
| | 1.00E-007 | 180.991519 | 96.21809 | 138.6048 | 42.38671 |
| | 1.00E-006 | 180.991519 | 65.832 | 123.4118 | 57.57976 |
| | 1.00E-005 | 96.2680389 | 42.41572 | 69.34188 | 26.92616 |
| | initial | 130.496299 | 73.56157 | 102.0289 | 28.46736 |
| **Achetylcholine** | 1.00E-009 | 130.496299 | 79.24897 | 104.8726 | 25.62366 |
| | 1.00E-008 | 130.496299 | 79.24897 | 104.8726 | 25.62366 |
| | 1.00E-007 | 130.496299 | 83.90056 | 107.1984 | 23.29787 |
| | 1.00E-006 | 148.316648 | 88.17124 | 118.2439 | 30.0727 |
| | 1.00E-005 | 156.535406 | 94.16393 | 125.3497 | 31.18574 |
| | **Time (sec)** | **Diameter (μm)** | | **Diameter (μm)** | |
| **$H_2O_2$ 300 μM** | initial | 124.715458 | 74.69668 | 99.70607 | 25.00939 |
| | 0 | 124.715458 | 74.69668 | 99.70607 | 25.00939 |
| | 10 | 112.048147 | 62.03723 | 87.04269 | 25.00546 |
| | 20 | 106.360952 | 53.805 | 80.08298 | 26.27798 |
| | 30 | 106.360952 | 53.805 | 80.08298 | 26.27798 |
| | 40 | 106.360952 | 45.576 | 75.96848 | 30.39248 |
| | 50 | 103.210061 | 42.411 | 72.81053 | 30.39953 |
| | 60 | 103.210061 | 41.7828 | 72.49643 | 30.71363 |
| | 70 | 106.391089 | 44.31452 | 75.3528 | 31.03828 |
| | 80 | 118.413303 | 57.60648 | 88.00989 | 30.40341 |
| | 90 | 119.67886 | 65.832 | 92.75543 | 26.92343 |
| | 100 | 122.209993 | 73.43073 | 97.82036 | 24.38963 |
| | 110 | 129.170778 | 75.96 | 102.5654 | 26.60539 |
| | 120 | 133.744373 | 77.23638 | 105.4904 | 28.254 |
| | 130 | 133.744373 | 85.455 | 109.5997 | 24.14469 |
| | 140 | 144.436402 | 85.455 | 114.9457 | 29.4907 |
| | 150 | 155.1315 | 84.822 | 119.9768 | 35.15475 |

| | | | | |
|---|---|---|---|---|
| 160 | 159.561203 | 89.88823 | 124.7247 | 34.83649 |
| 170 | 169.053671 | 89.88823 | 129.4709 | 39.58272 |
| 180 | 169.053671 | 89.88823 | 129.4709 | 39.58272 |
| 190 | 170.352283 | 89.88823 | 130.1203 | 40.23203 |
| 200 | 176.046846 | 89.88823 | 132.9675 | 43.07931 |
| 210 | 179.229563 | 89.88823 | 134.5589 | 44.67067 |
| 220 | 179.229563 | 89.88823 | 134.5589 | 44.67067 |
| 230 | 179.229563 | 89.88823 | 134.5589 | 44.67067 |
| 240 | 179.229563 | 89.88823 | 134.5589 | 44.67067 |
| 250 | 179.229563 | 89.88823 | 134.5589 | 44.67067 |
| 260 | 179.229563 | 89.88823 | 134.5589 | 44.67067 |
| 270 | 179.229563 | 89.88823 | 134.5589 | 44.67067 |
| 280 | 179.229563 | 89.88823 | 134.5589 | 44.67067 |
| 290 | 179.229563 | 89.88823 | 134.5589 | 44.67067 |
| 300 | 179.229563 | 89.88823 | 134.5589 | 44.67067 |
| 310 | 179.229563 | 89.88823 | 134.5589 | 44.67067 |
| 320 | 179.229563 | 94.95 | 137.0898 | 42.13978 |
| 340 | 179.229563 | 94.95 | 137.0898 | 42.13978 |
| 350 | 179.229563 | 94.95 | 137.0898 | 42.13978 |
| 360 | 179.229563 | 94.95 | 137.0898 | 42.13978 |
| 370 | 179.229563 | 94.95 | 137.0898 | 42.13978 |
| 380 | 183.02569 | 94.95 | 138.9878 | 44.03784 |
| 390 | 183.02569 | 94.95 | 138.9878 | 44.03784 |
| 400 | 183.02569 | 94.95 | 138.9878 | 44.03784 |
| 410 | 183.02569 | 94.95 | 138.9878 | 44.03784 |
| 420 | 183.02569 | 94.95 | 138.9878 | 44.03784 |
| 430 | 183.02569 | 94.95 | 138.9878 | 44.03784 |
| 440 | 183.02569 | 94.95 | 138.9878 | 44.03784 |
| 450 | 183.02569 | 94.95 | 138.9878 | 44.03784 |
| 460 | 183.02569 | 94.95 | 138.9878 | 44.03784 |
| 470 | 183.02569 | 94.95 | 138.9878 | 44.03784 |
| 480 | 183.02569 | 94.95 | 138.9878 | 44.03784 |
| 490 | 183.02569 | 94.95 | 138.9878 | 44.03784 |
| 500 | 183.02569 | 94.95 | 138.9878 | 44.03784 |

| | | | | | |
|---|---|---|---|---|---|
| | 510 | 183.02569 | 94.95 | 138.9878 | 44.03784 |
| | 520 | 183.02569 | 94.95 | 138.9878 | 44.03784 |
| | 530 | 183.02569 | 94.95 | 138.9878 | 44.03784 |
| | 540 | 183.02569 | 94.95 | 138.9878 | 44.03784 |
| | 550 | 183.02569 | 94.95 | 138.9878 | 44.03784 |
| | 560 | 183.02569 | 94.95 | 138.9878 | 44.03784 |
| | 570 | 183.02569 | 94.95 | 138.9878 | 44.03784 |
| | initial | 175.414118 | 80.391 | 127.9026 | 47.51156 |
| | **Concentration (M)** | **Diameter (μm)** | | **Diameter (μm)** | |
| **Norepinephrine** | 1.00E-009 | 175.414118 | 80.391 | 127.9026 | 47.51156 |
| | 1.00E-008 | 175.414118 | 81.657 | 128.5356 | 46.87856 |
| | 1.00E-007 | 169.719568 | 73.428 | 121.5738 | 48.14578 |
| | 1.00E-006 | 167.803515 | 67.10098 | 117.4522 | 50.35127 |
| | 1.00E-005 | 120.909628 | 41.7828 | 81.34621 | 39.56341 |
| | without $Ca^{2+}$ | 194.989693 | 87.99611 | 141.4929 | 53.49679 |

## Figure 1. Panel B/3 mM

| Date (number of experiments) | | *20120301* | *20130302* | *Average* | *± SEM* |
|---|---|---|---|---|---|
| | **Concentration (M)** | **Diameter (μm)** | | **Diameter (μm)** | |
| | before incubation | 175.597903 | 95.583 | 135.5905 | 40.00745 |
| | initial | 175.414118 | 97.482 | 136.4481 | 38.96606 |
| **Norepinephrine** | 1.00E-009 | 175.414118 | 96.8572796 | 136.1357 | 39.27842 |
| | 1.00E-008 | 175.414118 | 96.8572796 | 136.1357 | 39.27842 |
| | 1.00E-007 | 169.719568 | 96.2180889 | 132.9688 | 36.75074 |
| | 1.00E-006 | 167.803515 | 65.832 | 116.8178 | 50.98576 |
| | 1.00E-005 | 120.909628 | 42.4157222 | 81.66267 | 39.24695 |
| | initial | 130.496299 | 73.5615693 | 102.0289 | 28.46736 |
| **Achetylcholine** | 1.00E-009 | 130.496299 | 79.2489731 | 104.8726 | 25.62366 |
| | 1.00E-008 | 130.496299 | 79.2489731 | 104.8726 | 25.62366 |
| | 1.00E-007 | 130.496299 | 83.9005609 | 107.1984 | 23.29787 |
| | 1.00E-006 | 148.316648 | 88.171241 | 118.2439 | 30.0727 |
| | 1.00E-005 | 156.535406 | 94.1639343 | 125.3497 | 31.18574 |
| | **Time (sec)** | **Diameter (μm)** | | **Diameter (μm)** | |
| **$H_2O_2$ 3mM** | initial | 150.033014 | 70.2658485 | 110.1494 | 39.88358 |
| | 0 | 150.033014 | 70.2658485 | 110.1494 | 39.88358 |
| | 10 | 150.033014 | 84.1913738 | 117.1122 | 32.92082 |
| | 20 | 184.845755 | 87.3631785 | 136.1045 | 48.74129 |
| | 30 | 184.845755 | 86.721 | 135.7834 | 49.06238 |
| | 40 | 184.845755 | 90.5278557 | 137.6868 | 47.15895 |
| | 50 | 184.845755 | 94.3191269 | 139.5824 | 45.26331 |
| | 60 | 184.845755 | 98.115 | 141.4804 | 43.36538 |
| | 70 | 184.845755 | 98.115 | 141.4804 | 43.36538 |
| | 80 | 184.845755 | 98.115 | 141.4804 | 43.36538 |
| | 90 | 184.25628 | 98.115 | 141.1856 | 43.07064 |
| | 100 | 184.25628 | 98.115 | 141.1856 | 43.07064 |
| | 110 | 184.25628 | 98.115 | 141.1856 | 43.07064 |
| | 120 | 184.25628 | 98.115 | 141.1856 | 43.07064 |
| | 130 | 184.25628 | 98.115 | 141.1856 | 43.07064 |
| | 140 | 184.25628 | 98.115 | 141.1856 | 43.07064 |

| | | | | |
|---|---|---|---|---|
| 150 | 184.25628 | 98.115 | 141.1856 | 43.07064 |
| 160 | 184.25628 | 98.115 | 141.1856 | 43.07064 |
| 170 | 184.25628 | 98.115 | 141.1856 | 43.07064 |
| 180 | 184.25628 | 98.115 | 141.1856 | 43.07064 |
| 190 | 184.25628 | 98.115 | 141.1856 | 43.07064 |
| 200 | 184.207349 | 98.115 | 141.1612 | 43.04617 |
| 210 | 184.207349 | 98.115 | 141.1612 | 43.04617 |
| 220 | 184.207349 | 98.115 | 141.1612 | 43.04617 |
| 230 | 184.207349 | 98.115 | 141.1612 | 43.04617 |
| 240 | 184.207349 | 98.115 | 141.1612 | 43.04617 |
| 250 | 184.207349 | 98.115 | 141.1612 | 43.04617 |
| 260 | 184.207349 | 98.115 | 141.1612 | 43.04617 |
| 270 | 184.207349 | 98.115 | 141.1612 | 43.04617 |
| 280 | 184.207349 | 98.115 | 141.1612 | 43.04617 |
| 290 | 184.207349 | 98.115 | 141.1612 | 43.04617 |
| 300 | 184.207349 | 98.115 | 141.1612 | 43.04617 |
| 310 | 184.207349 | 98.115 | 141.1612 | 43.04617 |
| 320 | 184.207349 | 98.115 | 141.1612 | 43.04617 |
| 340 | 184.207349 | 98.115 | 141.1612 | 43.04617 |
| 350 | 184.207349 | 98.115 | 141.1612 | 43.04617 |
| 360 | 184.207349 | 98.115 | 141.1612 | 43.04617 |
| 370 | 184.207349 | 98.115 | 141.1612 | 43.04617 |
| 380 | 184.207349 | 98.115 | 141.1612 | 43.04617 |
| 390 | 184.207349 | 98.115 | 141.1612 | 43.04617 |
| 400 | 184.207349 | 98.115 | 141.1612 | 43.04617 |
| 410 | 184.207349 | 98.115 | 141.1612 | 43.04617 |
| 420 | 184.207349 | 98.115 | 141.1612 | 43.04617 |
| 430 | 184.207349 | 98.115 | 141.1612 | 43.04617 |
| 440 | 184.207349 | 98.115 | 141.1612 | 43.04617 |
| 450 | 184.207349 | 98.115 | 141.1612 | 43.04617 |
| 460 | 184.207349 | 98.115 | 141.1612 | 43.04617 |
| 470 | 184.207349 | 98.115 | 141.1612 | 43.04617 |
| 480 | 184.207349 | 98.115 | 141.1612 | 43.04617 |
| **Concentration (M)** | **Diameter (µm)** | | **Diameter (µm)** | |

| | | | | | |
|---|---|---|---|---|---|
| | initial | 185.496004 | 71.5318042 | 128.5139 | 56.9821 |
| **Norepinephrine** | 1.00E-009 | 185.496004 | 71.5318042 | 128.5139 | 56.9821 |
| | 1.00E-008 | 185.496004 | 74.694 | 130.095 | 55.401 |
| | 1.00E-007 | 179.166953 | 86.088 | 132.6275 | 46.53948 |
| | 1.00E-006 | 179.166953 | 74.6966776 | 126.9318 | 52.23514 |
| | 1.00E-005 | 179.166953 | 80.393494 | 129.7802 | 49.38673 |
| | without $Ca^{2+}$ | 194.989693 | 98.116 | 146.5528 | 48.43685 |

## Figure 2.

| Date (number of experiments) | | *20100831* | *20111212* | *20120223* | *20120228* | *20120229* | *Average* | *± SEM* |
|---|---|---|---|---|---|---|---|---|
| | **Concentration (M)** | **Diameter (μm)** | | | | | **Diameter (μm)** | |
| | before incubation | 173.391 | 163.7563 | 137.4543 | 196.4912 | 200.2382 | 174.2662 | 11.47788 |
| | initial | | 167.5 | 141.3306 | 184.8631 | 159.9224 | 163.404 | 9.021389 |
| **Norepinephrine** | 1.00E-009 | | 167.5 | 141.3306 | 184.8631 | 159.9224 | 163.404 | 9.021389 |
| | 1.00E-008 | | 167.5 | 143.4161 | 181.0557 | 155.3754 | 161.8368 | 8.075227 |
| | 1.00E-007 | | 162.4049 | 143.4161 | 149.7751 | 143.8304 | 149.8566 | 4.427781 |
| | 1.00E-006 | | 157.3524 | 128.5551 | 105.0856 | 46.27831 | 109.3179 | 23.57485 |
| | 1.00E-005 | | 74.91094 | 65.39843 | 77.226 | 32.38214 | 62.47938 | 10.35363 |
| | initial | | 137.6407 | 121.4601 | 117.738 | 127.2724 | 126.0278 | 4.339679 |
| **Achetylcholine** | 1.00E-009 | | 137.6407 | 121.4601 | 117.738 | 127.2724 | 126.0278 | 4.339679 |
| | 1.00E-008 | | 137.6407 | 123.7349 | 129.8638 | 127.2724 | 129.6279 | 2.951515 |
| | 1.00E-007 | | 152.9321 | 124.7845 | 151.4829 | 153.2461 | 145.6114 | 6.952905 |
| | 1.00E-006 | | 169.8387 | 128.1118 | 185.9426 | 182.0015 | 166.4737 | 13.23856 |
| | 1.00E-005 | | 169.5176 | 136.8481 | 190.3478 | 182.0015 | 169.6788 | 11.75067 |
| **endothelium removal** | | | | | | | | |
| | initial | 151.8712 | 144.8173 | 92.83544 | 167.4581 | 143.0594 | 140.0083 | 12.55375 |
| **Norepinephrine** | 1.00E-009 | 151.8712 | 144.8173 | 92.83544 | 167.4581 | 143.0594 | 140.0083 | 12.55375 |
| | 1.00E-008 | 151.8712 | 144.231 | 91.68235 | 165.5631 | 143.0594 | 139.2814 | 12.55721 |
| | 1.00E-007 | 145.545 | 144.231 | 98.45745 | 165.5631 | 137.3668 | 138.2327 | 10.99822 |
| | 1.00E-006 | 118.0167 | 91.3496 | 98.45745 | 165.5631 | 122.8281 | 119.243 | 12.98073 |
| | 1.00E-005 | 56.59118 | 75.23119 | 51.94073 | 106.4118 | 55.071 | 69.04918 | 10.19184 |
| | initial | 168.9628 | 140.429 | 106.5435 | 145.7564 | 119.719 | 136.2822 | 10.8021 |
| **Achetylcholine** | 1.00E-009 | 168.9628 | 140.429 | 106.9526 | 145.7564 | 119.719 | 136.364 | 10.74595 |
| | 1.00E-008 | 171.4932 | 140.429 | 110.2384 | 145.7564 | 118.4539 | 137.2742 | 10.81199 |
| | 1.00E-007 | 171.4932 | 140.429 | 108.7619 | 145.7564 | 118.4539 | 136.9789 | 10.99901 |
| | 1.00E-006 | 171.4932 | 142.9866 | 99.19536 | 145.788 | 118.4539 | 135.5834 | 12.38137 |
| | 1.00E-005 | 171.4932 | 142.9866 | 107.1342 | 147.0523 | 118.4539 | 137.424 | 11.31745 |
| | initial | 162.0179 | 142.0475 | 115.5758 | 130.4226 | 106.977 | 131.4082 | 9.743377 |
| **$H_2O_2$** | 1.00E-006 | 162.0179 | 142.0475 | 117.3374 | 130.4226 | 106.977 | 131.7605 | 9.605648 |
| | 3.00E-006 | 153.7495 | 140.5616 | 117.3374 | 130.4226 | 106.3459 | 129.6834 | 8.352012 |

| | | | | | | | | |
|---|---|---|---|---|---|---|---|---|
| | 1.00E-005 | 158.8905 | 136.8993 | 122.287 | 125.9924 | 102.5948 | 129.3328 | 9.237507 |
| | 3.00E-005 | 156.361 | 134.9062 | 122.287 | 128.538 | 89.96621 | 126.4117 | 10.76783 |
| | 1.00E-004 | 154.5105 | 128.84 | 122.287 | 129.1382 | 88.64034 | 124.6832 | 10.5632 |
| | 3.00E-004 | 147.7263 | 123.7786 | 119.0595 | 163.991 | 115.979 | 134.1069 | 9.328085 |
| | 1.00E-003 | 138.2566 | 155.8685 | 125.4075 | 171.5477 | 174.1061 | 153.0373 | 9.42448 |
| | 3.00E-003 | 172.5115 | 160.9277 | 122.8036 | 176.6172 | 179.782 | 162.5284 | 10.43183 |
| | 1.00E-002 | 174.0823 | 167.2905 | 127.1684 | 176.6172 | 179.782 | 164.9881 | 9.675821 |
| | initial | 168.9379 | 159.5524 | 104.891 | 173.4709 | 175.8442 | 156.5393 | 13.2093 |
| **Norepinephrine** | 1.00E-009 | 168.9379 | 159.5524 | 105.6503 | 173.4709 | 175.8442 | 156.6911 | 13.06088 |
| | 1.00E-008 | 168.9379 | 159.5524 | 105.6503 | 170.9393 | 175.8442 | 156.1848 | 12.90717 |
| | 1.00E-007 | 155.0343 | 154.1846 | 105.3142 | 170.9393 | 174.5818 | 152.0108 | 12.37373 |
| | 1.00E-006 | 100.13 | 154.1846 | 107.1865 | 169.6865 | 174.5818 | 141.1539 | 15.71317 |
| | 1.00E-005 | 62.24065 | 154.1846 | 98.5632 | 169.6865 | 174.5818 | 131.8513 | 22.03397 |
| | without $Ca^{2+}$ | 175.893 | 175.1489 | 137.4543 | 184.9054 | 185.7809 | 171.8365 | 8.873567 |

## Figure 3. Panel A

| Date (number of experiments) | | *20111121* | *201111212* | *20111123* | *20111124* | *20111129* | *Average* | *± SEM* |
|---|---|---|---|---|---|---|---|---|
| | **Concentration (M)** | **Diameter (μm)** | | | | | **Diameter (μm)** | |
| | before incubation | 166.479 | 158.5737605 | 196.280013 | 184.684181 | 176.475349 | 176.4985 | 6.633253 |
| | initial | 141.171768 | 150.4530605 | 178.793078 | 116.858383 | 141.228522 | 145.701 | 9.975172 |
| **Norepinephrine** | 1.00E-09 | 141.171768 | 150.4530605 | 178.793078 | 116.858383 | 137.413495 | 144.938 | 10.08923 |
| | 1.00E-08 | 141.171768 | 146.5733339 | 178.793078 | 109.866993 | 136.096469 | 142.5003 | 11.04867 |
| | 1.00E-07 | 128.513034 | 146.5733339 | 178.793078 | 73.8198523 | 134.1975 | 132.3794 | 17.03404 |
| | 0.000001 | 113.308766 | 144.0474866 | 178.793078 | 53.2322489 | 75.9837312 | 113.0731 | 22.62204 |
| | 0.00001 | 68.364 | 66.29900841 | 98.1333697 | 34.2347099 | 36.7194565 | 60.75011 | 11.76182 |
| | initial | 118.479275 | 133.7129134 | 151.308193 | 105.165671 | 110.83466 | 123.9001 | 8.362311 |
| **Achetylcholine** | 1.00E-09 | 118.479275 | 133.7129134 | 151.308193 | 105.165671 | 110.83466 | 123.9001 | 8.362311 |
| | 1.00E-08 | 118.479275 | 142.7411519 | 151.308193 | 105.165671 | 110.83466 | 125.7058 | 9.058024 |
| | 1.00E-07 | 140.890507 | 156.3394667 | 163.392486 | 123.869219 | 110.83466 | 139.0653 | 9.801607 |
| | 0.000001 | 152.659331 | 161.5155014 | 181.829731 | 174.56115 | 141.978387 | 162.5088 | 7.204353 |
| | 0.00001 | 165.223913 | 161.5155014 | 183.679104 | 181.263608 | 152.637024 | 168.8638 | 5.932129 |
| **PLA inhibitor** | | 120.376566 | 130.6987826 | 170.616699 | 124.527362 | 105.399729 | 130.3238 | 10.90278 |
| **$H_2O_2$** | initial | 123.734909 | 130.6987826 | 170.616699 | 124.527362 | 105.399729 | 130.9955 | 10.76946 |
| | 1.00E-06 | 123.734909 | 130.6987826 | 170.616699 | 124.527362 | 105.399729 | 130.9955 | 10.76946 |
| | 3.00E-06 | 123.734909 | 128.8493275 | 174.581818 | 125.742551 | 105.399729 | 131.6617 | 11.48392 |
| | 1.00E-05 | 116.349806 | 126.0957585 | 168.175611 | 124.082534 | 101.944447 | 127.3296 | 11.05763 |
| | 3.00E-05 | 112.617093 | 124.8952487 | 156.362533 | 110.95029 | 98.1476691 | 120.5946 | 9.895349 |
| | 1.00E-04 | 116.17749 | 126.8276774 | 155.846607 | 103.460166 | 95.6165237 | 119.5857 | 10.52015 |
| | 3.00E-04 | 134.916645 | 149.8713588 | 160.329038 | 103.460166 | 110.776804 | 131.8708 | 10.9437 |
| | 1.00E-03 | 166.194739 | 156.6390467 | 192.807477 | 188.912058 | 141.164678 | 169.1436 | 9.743333 |
| | 3.00E-03 | 166.50908 | 162.8188674 | 197.062357 | 188.912058 | 151.308193 | 173.3221 | 8.508801 |
| | 1.00E-02 | 169.686519 | 162.8188674 | 197.062357 | 188.912058 | 154.534999 | 174.603 | 7.984153 |
| | initial | 164.449703 | 149.4657641 | 187.40648 | 184.93678 | 134.1975 | 164.0912 | 10.21226 |
| **Norepinephrine** | 1.00E-09 | 164.449703 | 150.8785757 | 187.40648 | 184.93678 | 134.1975 | 164.3738 | 10.11454 |
| | 1.00E-08 | 161.319398 | 150.8785757 | 187.40648 | 183.675844 | 130.399538 | 162.736 | 10.57204 |
| | 1.00E-07 | 161.319398 | 149.0577259 | 185.507885 | 183.675844 | 125.340393 | 160.9802 | 11.24535 |
| | 0.000001 | 161.319398 | 149.0577259 | 185.507885 | 183.675844 | 125.340393 | 160.9802 | 11.24535 |
| | 0.00001 | 159.973773 | 149.0577259 | 185.507885 | 183.045376 | 125.340393 | 160.585 | 11.18269 |
| | without $Ca^{2+}$ | 172.738256 | 164.0691627 | 197.549761 | 190.106662 | 156.454755 | 176.1837 | 7.740225 |

## Figure 3. Panel B

| Date (number of experiments) | Concentration (M) | *20111201* | *20111202* | *20111206* | *20111207* | *20111208* | *Average* | *± SEM* |
|---|---|---|---|---|---|---|---|---|
| | | Diameter (μm) | | | | | Diameter (μm) | |
| | before incubation | 166.479 | 186.8648 | 177.8741 | 190.094 | 167.8596 | 177.8343 | 4.797339 |
| | initial | 98.31898 | 183.3571 | 145.788 | 159.4255 | 156.8576 | 148.7494 | 14.01679 |
| **Norepinephrine** | 1.00E-09 | 98.31898 | 180.1427 | 145.788 | 159.4255 | 156.8576 | 148.1066 | 13.62937 |
| | 1.00E-08 | 93.89761 | 180.1427 | 145.788 | 159.4255 | 156.8576 | 147.2223 | 14.44142 |
| | 1.00E-07 | 83.84562 | 177.625 | 143.8916 | 149.3464 | 149.85 | 140.9117 | 15.43425 |
| | 0.000001 | 53.98715 | 168.1172 | 143.8916 | 146.8683 | 144.8575 | 131.5443 | 19.89624 |
| | 0.00001 | 37.53962 | 65.39536 | 48.212 | 62.63822 | 78.75955 | 58.50895 | 7.142479 |
| | initial | 85.53936 | 139.411 | 118.7512 | 146.9051 | 134.1975 | 124.9608 | 10.88183 |
| **Achetylcholine** | 1.00E-09 | 85.53936 | 139.411 | 118.7512 | 146.9051 | 134.1975 | 124.9608 | 10.88183 |
| | 1.00E-08 | 85.53936 | 139.411 | 138.6284 | 153.9492 | 134.1975 | 130.3451 | 11.68408 |
| | 1.00E-07 | 109.025 | 151.8475 | 138.6284 | 175.1215 | 134.1975 | 141.764 | 10.84741 |
| | 0.000001 | 135.7516 | 163.2858 | 169.5223 | 183.3319 | 149.3679 | 160.2519 | 8.205366 |
| | 0.00001 | 143.6924 | 180.9683 | 176.8156 | 185.7119 | 162.7992 | 169.9975 | 7.607761 |
| **cheleryrine** | | 75.49703 | 130.616 | 117.7992 | 146.9392 | 134.7636 | 121.123 | 12.31957 |
| **$H_2O_2$** | initial | 127.2582 | 192.1079 | 163.5898 | 172.7731 | 163.1876 | 163.7833 | 10.5299 |
| | 1.00E-06 | 127.2582 | 192.1079 | 163.5898 | 172.7731 | 163.1876 | 163.7833 | 10.5299 |
| | 3.00E-06 | 127.2582 | 185.8747 | 160.394 | 169.3426 | 156.1163 | 159.7972 | 9.601675 |
| | 1.00E-05 | 127.2582 | 190.2373 | 160.394 | 168.1125 | 153.288 | 159.858 | 10.24021 |
| | 3.00E-05 | 124.0938 | 190.2373 | 160.394 | 168.1125 | 153.3402 | 159.2356 | 10.74929 |
| | 1.00E-04 | 119.0646 | 190.2373 | 160.394 | 168.1125 | 149.1316 | 157.388 | 11.70465 |
| | 3.00E-04 | 121.536 | 193.3553 | 164.2217 | 182.6948 | 165.7445 | 165.5105 | 12.25991 |
| | 1.00E-03 | 135.4635 | 193.3553 | 176.3425 | 186.4129 | 176.1515 | 173.5451 | 10.05697 |
| | 3.00E-03 | 158.9448 | 193.3553 | 175.8783 | 185.1522 | 176.1515 | 177.8964 | 5.735447 |
| | 1.00E-02 | 160.8131 | 193.3553 | 177.7817 | 185.1522 | 176.1515 | 178.6508 | 5.402202 |
| | initial | 161.694 | 185.4431 | 169.4608 | 178.7673 | 175.1764 | 174.1083 | 4.043745 |
| **Norepinephrine** | 1.00E-09 | 161.694 | 185.4431 | 169.4608 | 178.7673 | 168.699 | 172.8128 | 4.163931 |
| | 1.00E-08 | 161.694 | 185.4431 | 169.9991 | 176.9594 | 168.699 | 172.5589 | 4.030286 |
| | 1.00E-07 | 161.694 | 179.7196 | 169.9991 | 176.9594 | 162.5677 | 170.188 | 3.653471 |
| | 0.000001 | 161.694 | 179.7196 | 168.7453 | 176.9594 | 162.5677 | 169.9372 | 3.665299 |
| | 0.00001 | 161.0621 | 178.0026 | 165.6115 | 176.9594 | 162.5677 | 168.8407 | 3.606515 |
| | without $Ca^{2+}$ | 166.0018 | 195.7854 | 179.026 | 189.4458 | 176.6989 | 185.239 | 4.476405 |

# Figure 3. Panel C

| Date (number of experiments) | | *20140508* | *20140508d* | *20140513* | *20140513d* | *Average* | *± SEM* |
|---|---|---|---|---|---|---|---|
| | **Concentration (M)** | **Diameter (µm)** | | | | **Diameter (µm)** | |
| | initial | 113.314071 | 124.8311 | 182.964371 | 148.1612 | 142.3177 | 15.36579 |
| **U46619** | 0.000000001 | 113.314071 | 124.8311 | 182.964371 | 148.1612 | 142.3177 | 15.36579 |
| | 0.00000001 | 108.259654 | 124.8311 | 182.964371 | 148.1612 | 141.0541 | 16.19065 |
| | 0.0000001 | 104.462262 | 124.8311 | 182.964371 | 141.0113 | 138.3173 | 16.65498 |
| | 0.000001 | 73.4716454 | 70.33425 | 102.563578 | 73.07793 | 79.86185 | 7.599342 |
| | 0.000001 | 29.751 | 29.77792 | 43.7182653 | 50.04304 | 38.32256 | 5.106906 |
| | initial | 127.258187 | 121.0686 | 127.966242 | 136.7177 | 128.2527 | 3.218948 |
| **Achetylcholine** | 0.000000001 | 127.258187 | 121.0686 | 133.026425 | 135.1199 | 129.1183 | 3.156352 |
| | 0.00000001 | 127.258187 | 127.3904 | 133.026425 | 135.1199 | 130.6987 | 1.994737 |
| | 0.0000001 | 127.258187 | 136.273 | 133.026425 | 169.9579 | 141.6289 | 9.625207 |
| | 0.000001 | 146.868274 | 141.5686 | 147.65327 | 179.3178 | 153.852 | 8.595484 |
| | 0.000001 | 155.800322 | 148.7765 | 183.727092 | 188.9566 | 169.3151 | 9.991561 |
| **U46619** | | 123.441488 | 132.4484 | 168.013512 | 140.1377 | 141.0103 | 9.625941 |
| | initial | 117.105 | 132.4196 | 178.596892 | 140.1377 | 142.0648 | 13.08412 |
| | 0.000000001 | 117.105 | 132.4196 | 178.596892 | 140.1377 | 142.0648 | 13.08412 |
| | 0.00000001 | 110.775 | 131.7613 | 169.739621 | 131.9088 | 136.0462 | 12.27925 |
| | 0.0000001 | 105.078 | 130.0257 | 149.522057 | 117.8978 | 125.6309 | 9.453056 |
| | 0.000001 | 53.86454 | 75.98373 | 87.0530338 | 56.90314 | 68.45111 | 7.899871 |
| | 0.000001 | 27.2263618 | 39.879 | 40.1244141 | 40.2789 | 36.87717 | 3.217989 |
| **U73122** | | 134.1975 | 123.5664 | 134.830488 | 138.1188 | 132.6783 | 3.15655 |
| **$H_2O_2$** | initial | 119.005684 | 119.4342 | 134.830488 | 130.8168 | 126.0218 | 4.012553 |
| | 1.00E-06 | 119.005684 | 119.4342 | 134.830488 | 130.8168 | 126.0218 | 4.012553 |
| | 3.00E-06 | 113.94176 | 119.4342 | 153.851562 | 140.0433 | 131.8177 | 9.246854 |
| | 1.00E-05 | 113.94176 | 116.2275 | 157.637332 | 148.295 | 134.0254 | 11.1103 |
| | 3.00E-05 | 107.611861 | 113.653 | 162.067781 | 153.3402 | 134.1682 | 13.76004 |
| | 1.00E-04 | 97.5004963 | 102.7412 | 169.029971 | 157.5191 | 131.6977 | 18.41279 |
| | 3.00E-04 | 95.5850952 | 138.6328 | 189.318868 | 180.1071 | 150.911 | 21.48505 |
| | 1.00E-03 | 128.500557 | 145.0951 | 195.088309 | 192.1736 | 165.2144 | 16.76291 |
| | 3.00E-03 | 151.288323 | 145.0951 | 195.088309 | 193.3336 | 171.2013 | 13.34942 |
| | 1.00E-02 | 155.086291 | 153.3167 | 195.088309 | 195.6666 | 174.7895 | 11.89255 |
| | without $Ca^{2+}$ | 155.719285 | 158.9448 | 200.044034 | 200.1662 | 178.7186 | 12.36509 |

## Figure 3. Panel D

| Date (number of experiments) | Concentration (M) | 20111214 | 20111215 | 20111219 | 20111220 | 20120103 | Average | ± SEM |
|---|---|---|---|---|---|---|---|---|
| | | Diameter (µm) | | | | | Diameter (µm) | |
| | before incubation | 189.927428 | 199.294492 | 188.209744 | 201.14365 | 182.849374 | 192.2849 | 3.455471 |
| | initial | 176.066203 | 183.606005 | 154.457184 | 187.991416 | 169.829317 | 174.39 | 5.877329 |
| **Norepinephrine** | 1.00E-09 | 176.066203 | 183.606005 | 154.457184 | 187.991416 | 169.829317 | 174.39 | 5.877329 |
| | 1.00E-08 | 176.066203 | 183.606005 | 152.554317 | 187.991416 | 169.829317 | 174.0095 | 6.203288 |
| | 1.00E-07 | 176.066203 | 178.596892 | 150.022336 | 187.991416 | 169.829317 | 172.5012 | 6.33345 |
| | 0.000001 | 158.075197 | 174.121032 | 110.149273 | 164.014206 | 108.324404 | 142.9368 | 13.99808 |
| | 0.00001 | 70.9214276 | 99.77534 | 68.364 | 101.684702 | 94.52282 | 87.05366 | 7.215432 |
| | initial | 141.300862 | 145.404107 | 125.99244 | 154.339111 | 114.824523 | 136.3722 | 7.073609 |
| **Achetylcholine** | 1.00E-09 | 141.300862 | 145.404107 | 125.99244 | 154.339111 | 114.824523 | 136.3722 | 7.073609 |
| | 1.00E-08 | 141.300862 | 145.404107 | 134.901789 | 154.339111 | 114.824523 | 138.1541 | 6.630483 |
| | 1.00E-07 | 157.844361 | 155.969972 | 137.871571 | 181.210543 | 114.824523 | 149.5442 | 11.07846 |
| | 0.000001 | 166.777187 | 183.849178 | 184.863105 | 197.098963 | 142.336348 | 174.985 | 9.482828 |
| | 0.00001 | 185.053727 | 185.900573 | 195.622599 | 197.098963 | 158.595245 | 184.4542 | 6.912887 |
| **Src inhibitor-1** | | 103.674886 | 158.352508 | 126.601583 | 160.364024 | 139.329041 | 137.6644 | 10.54446 |
| **$H_2O_2$** | initial | 112.888948 | 177.69157 | 129.766545 | 167.389906 | 129.32114 | 143.4116 | 12.38232 |
| | 1.00E-06 | 112.888948 | 177.69157 | 129.766545 | 167.389906 | 129.32114 | 143.4116 | 12.38232 |
| | 3.00E-06 | 116.011828 | 171.659757 | 129.766545 | 163.407209 | 133.717414 | 142.9126 | 10.55332 |
| | 1.00E-05 | 116.011828 | 172.924883 | 117.132371 | 162.552868 | 130.913211 | 139.907 | 11.77618 |
| | 3.00E-05 | 111.587683 | 170.394624 | 117.132371 | 157.865934 | 124.707425 | 136.3376 | 11.70472 |
| | 1.00E-04 | 98.9506803 | 170.394624 | 107.611861 | 160.262801 | 128.424141 | 133.1288 | 14.08259 |
| | 3.00E-04 | 84.9069866 | 167.841703 | 115.840728 | 179.763075 | 147.668202 | 139.2041 | 17.36492 |
| | 1.00E-03 | 93.7887299 | 191.925359 | 182.313894 | 202.501644 | 173.036076 | 168.7131 | 19.36208 |
| | 3.00E-03 | 168.578959 | 191.925359 | 185.576983 | 196.994246 | 174.987583 | 183.6126 | 5.255618 |
| | 1.00E-02 | 181.804386 | 191.808406 | 184.93678 | 202.041073 | 174.987583 | 187.1156 | 4.608889 |
| | initial | 181.760304 | 177.258078 | 165.232395 | 206.4638 | 177.552829 | 181.6535 | 6.787305 |
| **Norepinephrine** | 1.00E-09 | 181.760304 | 177.258078 | 165.232395 | 206.4638 | 177.552829 | 181.6535 | 6.787305 |
| | 1.00E-08 | 181.760304 | 177.258078 | 165.232395 | 196.432041 | 177.552829 | 179.6471 | 5.020344 |
| | 1.00E-07 | 181.760304 | 174.726351 | 165.232395 | 196.432041 | 177.552829 | 179.1408 | 5.105397 |
| | 0.000001 | 181.042425 | 174.726351 | 165.232395 | 196.432041 | 174.369383 | 178.3605 | 5.17328 |
| | 0.00001 | 181.042425 | 174.726351 | 165.232395 | 196.432041 | 174.369383 | 178.3605 | 5.17328 |
| | without $Ca^{2+}$ | 184.291069 | 193.069152 | 186.106311 | 204.501132 | 179.776463 | 189.5488 | 4.306728 |

## Figure 4. Panel A

| Date (number of experiments) | | 20111026 | 20111108 | 20111109 | 20111108 | 2011110 | Average | ± SEM |
|---|---|---|---|---|---|---|---|---|
| | Concentration (M) | Diameter (μm) | | | | | Diameter (μm) | |
| | before incubation | 160.81315 | 137.014895 | 154.743579 | 141.996731 | 152.45973 | 149.4056 | 0.946642 |
| | initial | 118.540125 | 90.5986441 | 145.006741 | 87.8297438 | 144.07531 | 117.2101 | 2.702528 |
| Norepinephrine | 1.00E-09 | 118.540125 | 90.5986441 | 145.006741 | 86.6077183 | 145.32555 | 117.2158 | 2.764389 |
| | 1.00E-08 | 118.540125 | 90.5986441 | 145.006741 | 80.7788328 | 147.72924 | 116.5307 | 2.983661 |
| | 1.00E-07 | 114.334941 | 92.6345303 | 137.397461 | 56.8573513 | 145.36967 | 109.3188 | 3.516379 |
| | 0.000001 | 91.3298603 | 101.376881 | 117.765219 | 41.9598293 | 134.54488 | 97.39533 | 3.45595 |
| | 0.00001 | 39.1284139 | 110.258352 | 39.6169 | 21.0418948 | 76.351973 | 57.27951 | 3.544227 |
| | initial | 97.6873072 | 90.55441 | 106.351533 | 60.8799904 | 122.35253 | 95.56515 | 2.261703 |
| Achetylcholine | 1.00E-09 | 97.6873072 | 90.55441 | 98.2802636 | 59.9080252 | 122.42455 | 93.77091 | 2.243738 |
| | 1.00E-08 | 100.887572 | 90.55441 | 98.2802636 | 58.1016774 | 125.4714 | 94.65906 | 2.438433 |
| | 1.00E-07 | 124.758825 | 88.6561633 | 99.4313931 | 85.2319878 | 137.25449 | 107.0666 | 2.362334 |
| | 0.000001 | 151.925273 | 88.6561633 | 145.822374 | 120.843327 | 145.73442 | 130.5963 | 2.789435 |
| | 0.00001 | 167.116792 | 31.017 | 154.49869 | 131.618342 | 159.55869 | 128.7619 | 6.134443 |
| indomethacin | | 134.763598 | 88.62 | 106.345886 | 96.6025161 | 129.9933 | 111.2651 | 2.219311 |
| $H_2O_2$ | initial | 148.144991 | 88.62 | 91.3298603 | 96.6025161 | 129.9933 | 110.9381 | 2.995536 |
| | 1.00E-06 | 148.144991 | 88.62 | 91.3298603 | 90.5809517 | 130.31347 | 109.7979 | 3.210125 |
| | 3.00E-06 | 150.09577 | 98.7500256 | 87.5830511 | 93.6604714 | 130.78001 | 112.1739 | 3.248139 |
| | 1.00E-05 | 150.09577 | 98.7500256 | 84.7227393 | 94.1660612 | 130.78001 | 111.7029 | 3.44669 |
| | 3.00E-05 | 156.871649 | 106.984495 | 84.7227393 | 116.721149 | 130.78001 | 119.216 | 3.503807 |
| | 1.00E-04 | 168.04927 | 111.424186 | 84.7227393 | 137.388713 | 146.60476 | 129.6379 | 4.395122 |
| | 3.00E-04 | 174.345253 | 111.424186 | 97.2536073 | 140.967264 | 158.00294 | 136.3987 | 4.564515 |
| | 1.00E-03 | 172.392302 | 115.840728 | 147.522948 | 140.967264 | 162.95786 | 147.9362 | 3.287897 |
| | 3.00E-03 | 172.392302 | 121.537652 | 155.723146 | 139.640724 | 162.54178 | 150.3671 | 3.210771 |
| | 1.00E-02 | 173.022188 | 121.537652 | 155.723146 | 140.249151 | 162.54178 | 150.6148 | 3.454926 |
| | initial | 164.342454 | | 150.654 | 134.358624 | 158.14869 | 151.8759 | 2.404763 |
| Norepinephrine | 1.00E-09 | 164.342454 | | 150.654 | 127.439111 | 163.14339 | 151.3947 | 3.42558 |
| | 1.00E-08 | 164.342454 | | 150.654 | 129.135102 | 163.14339 | 151.8187 | 3.565699 |
| | 1.00E-07 | 161.899574 | | 149.388 | 127.2078 | 160.94639 | 149.8604 | 3.913787 |
| | 0.000001 | 161.899574 | | 147.489 | 128.729545 | 156.33947 | 148.6144 | 4.028254 |
| | 0.00001 | 161.16671 | | 147.489 | 127.302263 | 154.14557 | 147.5259 | 4.864416 |
| | without $Ca^{2+}$ | 173.815856 | 137.014895 | 155.800322 | 141.126356 | 171.95361 | 155.9422 | 7.594752 |

# Figure 4. Panel B

| Date (number of experiments) | | *20120704* | *20120704d* | *20120425* | *20120704* | *20120704d* | *Average* | *± SEM* |
|---|---|---|---|---|---|---|---|---|
| | **Concentration (M)** | **Diameter (μm)** | | | | | **Diameter (μm)** | |
| | initial | 142.0700769 | 148.1436 | 143.7133006 | 142.0700769 | 148.1436 | 144.8281 | 1.386393121 |
| **Achetylcholine** | 1.00E-09 | 142.0700769 | 148.1436 | 143.7133006 | 142.0700769 | 148.1436 | 144.8281 | 1.386393121 |
| | 1.00E-08 | 142.0700769 | 148.1436 | 143.7133006 | 142.0700769 | 148.1436 | 144.8281 | 1.386393121 |
| | 1.00E-07 | 142.0700769 | 172.8102 | 143.7133006 | 142.0700769 | 172.8102 | 154.6948 | 7.401665404 |
| | 0.000001 | 164.3424541 | 195.6011 | 149.4000713 | 164.3424541 | 195.6011 | 173.8574 | 9.286565315 |
| | 0.00001 | 180.3605697 | 204.5775 | 184.2910693 | 180.3605697 | 204.5775 | 190.8335 | 5.656699411 |
| | initial | 150.154487 | 160.843 | 181.7415668 | 150.154487 | 160.843 | 160.7473 | 5.767117771 |
| **Norepinephrine** | 1.00E-09 | 150.154487 | 160.843 | 178.5778202 | 150.154487 | 160.843 | 160.1146 | 5.197881628 |
| | 1.00E-08 | 150.154487 | 158.9851 | 176.6625774 | 150.154487 | 158.9851 | 158.9884 | 4.839693197 |
| | 1.00E-07 | 143.1979753 | 131.1288 | 176.6625774 | 143.1979753 | 131.1288 | 145.0632 | 8.348092029 |
| | 0.000001 | 54.49685001 | 79.758 | 176.6625774 | 54.49685001 | 79.758 | 89.03446 | 22.62353353 |
| | 0.00001 | 27.85919088 | 34.18786 | 75.92306445 | 27.85919088 | 34.18786 | 40.00343 | 9.090728515 |
| **SC-560** | | 101.4637979 | 136.1185 | 137.3741284 | 101.4637979 | 136.1185 | 122.5078 | 8.594220941 |
| **$H_2O_2$** | initial | 79.45095702 | 131.251 | 137.3741284 | 79.45095702 | 136.1185 | 112.7291 | 13.62419459 |
| | 0.000001 | 79.45095702 | 131.251 | 137.3741284 | 79.45095702 | 131.251 | 111.7556 | 13.23561314 |
| | 0.000003 | 83.4264249 | 131.251 | 136.7411854 | 83.4264249 | 131.251 | 113.2192 | 12.20408403 |
| | 0.00001 | 87.02541603 | 131.251 | 138.6400082 | 87.02541603 | 131.251 | 115.0386 | 11.51561195 |
| | 0.00003 | 91.04864376 | 129.9871 | 138.6400082 | 91.04864376 | 129.9871 | 116.1423 | 10.365541 |
| | 0.0001 | 91.04864376 | 167.2905 | 139.9058879 | 91.04864376 | 167.2905 | 131.3168 | 17.18289838 |
| | 0.0003 | 138.6674614 | 187.0223 | 141.8047233 | 138.6674614 | 187.0223 | 158.6369 | 11.60246181 |
| | 0.001 | 168.8888563 | 203.3694 | 172.2178793 | 168.8888563 | 203.3694 | 183.3469 | 8.196728101 |
| | 0.003 | 175.1214756 | 204.8261 | 201.294 | 175.1214756 | 204.8261 | 192.2378 | 7.017421783 |
| | 0.01 | 175.1214756 | 204.8261 | 196.9647484 | 175.1214756 | 204.8261 | 191.372 | 6.787729175 |
| | initial | 172.1597129 | 198.0794 | 191.1701905 | 172.1597129 | 198.0794 | 186.3297 | 5.920813323 |
| **Norepinephrine** | 1.00E-09 | 172.5224029 | 198.0794 | 187.368 | 172.5224029 | 198.0794 | 185.7143 | 5.729663291 |
| | 1.00E-08 | 172.5224029 | 198.0794 | 187.368 | 172.5224029 | 198.0794 | 185.7143 | 5.729663291 |
| | 1.00E-07 | 172.5224029 | 198.0794 | 187.368 | 172.5224029 | 198.0794 | 185.7143 | 5.729663291 |
| | 0.000001 | 172.5224029 | 198.0794 | 187.368 | 172.5224029 | 198.0794 | 185.7143 | 5.729663291 |
| | 0.00001 | 172.5224029 | 198.0794 | 184.8370824 | 172.5224029 | 198.0794 | 185.2082 | 5.7154821 |
| | without $Ca^{2+}$ | 191.417358 | 207.7484 | 200.0640494 | 191.417358 | 207.7484 | 199.6791 | 3.653008981 |

## Figure 4. Panel B

| Date (number of experiments) | | *20120327* | *20120404* | *20120417* | *20120418* | *Average* | *± SEM* |
|---|---|---|---|---|---|---|---|
| | **Concentration (M)** | **Diameter (μm)** | | | | **Diameter (μm)** | |
| | before incubation | 184.33455 | 201.11575 | 170.44634 | 209.5316 | 191.3571 | 8.71837 |
| | initial | 162.54178 | 160.16902 | 86.72331 | 175.97514 | 146.3523 | 20.17864 |
| **Norepinephrine** | 1.00E-09 | 162.54178 | 160.16902 | 86.72331 | 198.1381 | 151.8931 | 23.39442 |
| | 1.00E-08 | 162.54178 | 155.80032 | 88.62226 | 198.1381 | 151.2756 | 22.85632 |
| | 1.00E-07 | 139.77838 | 113.96285 | 114.57475 | 195.7209 | 141.0092 | 19.20323 |
| | 0.000001 | 112.76109 | 68.05854 | 138.64001 | 191.27078 | 127.6826 | 25.72483 |
| | 0.00001 | 53.266114 | 24.654514 | 137.94318 | 49.374 | 66.30945 | 24.70402 |
| | initial | 132.29852 | 142.52765 | 101.94445 | 133.60049 | 127.5928 | 8.846487 |
| **Achetylcholine** | 1.00E-09 | 132.29852 | 142.52765 | 101.94445 | 133.60049 | 127.5928 | 8.846487 |
| | 1.00E-08 | 132.29852 | 142.52765 | 101.94445 | 133.60049 | 127.5928 | 8.846487 |
| | 1.00E-07 | 160.154 | 179.65159 | 101.94445 | 133.60049 | 143.8376 | 16.85403 |
| | 0.000001 | 168.40775 | 185.53057 | 69.043443 | 177.25808 | 150.06 | 27.23083 |
| | 0.00001 | 178.506 | 190.71063 | 25.327913 | 197.5457 | 148.0226 | 41.08734 |
| **celecoxib** | | 143.19798 | 168.54925 | 114.08233 | 165.94382 | 147.9433 | 12.64156 |
| **$H_2O_2$** | initial | 173.45239 | 147.20483 | 109.51632 | 154.45718 | 146.1577 | 13.40885 |
| | 0.000001 | 173.45239 | 147.20483 | 109.51632 | 154.45718 | 146.1577 | 13.40885 |
| | 0.000003 | 173.45239 | 136.74119 | 105.08563 | 154.45718 | 142.4341 | 14.53159 |
| | 0.00001 | 173.45239 | 132.30306 | 101.92086 | 154.45718 | 140.5334 | 15.37367 |
| | 0.00003 | 171.55351 | 131.03253 | 93.053152 | 144.33649 | 134.9939 | 16.32638 |
| | 0.0001 | 167.13118 | 128.59875 | 86.730242 | 131.03711 | 128.3743 | 16.44046 |
| | 0.0003 | 167.13118 | 124.7797 | 108.89255 | 141.17177 | 135.4938 | 12.4351 |
| | 0.001 | 163.96655 | 162.681 | 117.3511 | 161.45967 | 151.3646 | 11.34937 |
| | 0.003 | 174.8467 | 176.22998 | 153.92446 | 206.36673 | 177.842 | 10.79073 |
| | 0.01 | 177.37671 | 182.55109 | 152.553 | 206.36673 | 179.7119 | 11.0363 |
| | initial | 161.72747 | 165.85687 | 139.26 | 199.49545 | 166.5849 | 12.4294 |
| **Norepinephrine** | 1.00E-09 | 161.72747 | 165.85687 | 139.26 | 196.3535 | 165.7995 | 11.74194 |
| | 1.00E-08 | 159.11863 | 165.85687 | 139.26 | 196.3535 | 165.1473 | 11.83507 |
| | 1.00E-07 | 159.11863 | 154.48442 | 139.26 | 196.3535 | 162.3041 | 12.1164 |
| | 0.000001 | 159.11863 | 154.48442 | 139.26 | 196.3535 | 162.3041 | 12.1164 |
| | 0.00001 | 159.6428 | 154.48442 | 135.462 | 211.60764 | 165.2992 | 16.28825 |
| | without $Ca^{2+}$ | 183.61474 | 187.50695 | 155.67811 | 211.60764 | 184.6019 | 11.45756 |

## Figure 5. Panel A

| Date (number of experiments) | | 20110927 | 20110928 | 20110929 | 20111004 | 20111005 | Average | ± SEM |
|---|---|---|---|---|---|---|---|---|
| | Concentration (M) | Diameter (µm) | | | | | Diameter (µm) | |
| | before incubation | 186.189183 | 191.808406 | 176.934552 | 215.279578 | 196.37697 | 193.3177 | 6.376459 |
| | initial | 160.194032 | 148.208544 | 138.04045 | 149.614468 | 177.402694 | 154.692 | 6.675098 |
| **Norepinephrine** | 1.00E-09 | 151.447155 | 148.841164 | 138.04045 | 149.614468 | 177.402694 | 153.0692 | 6.520831 |
| | 1.00E-08 | 147.65327 | 148.841164 | 127.121149 | 144.462747 | 169.181612 | 147.452 | 6.694869 |
| | 1.00E-07 | 145.155882 | 148.841164 | 117.4586 | 138.130399 | 169.181612 | 143.7535 | 8.35907 |
| | 0.000001 | 145.155882 | 91.231087 | 111.184785 | 104.031778 | 163.854101 | 123.0915 | 13.54355 |
| | 0.00001 | 114.152561 | 89.3337771 | 48.3737904 | 61.5183519 | 67.7783104 | 76.23136 | 11.56214 |
| | initial | 130.894848 | 144.358701 | 124.445293 | 146.573334 | 163.069687 | 141.8684 | 6.714969 |
| **Acethylcholine** | 1.00E-09 | 134.103411 | 144.358701 | 124.445293 | 146.573334 | 163.069687 | 142.5101 | 6.479327 |
| | 1.00E-08 | 137.255954 | 144.358701 | 138.128956 | 180.494924 | 163.069687 | 152.6616 | 8.36807 |
| | 1.00E-07 | 177.135973 | 184.905351 | 154.85229 | 196.239197 | 163.069687 | 175.2405 | 7.422113 |
| | 0.000001 | 185.115432 | 185.179276 | 179.514382 | 202.5956 | 163.069687 | 183.0949 | 6.334563 |
| | 0.00001 | 193.661799 | 186.283836 | 181.966307 | 202.679637 | 189.904209 | 190.8992 | 3.524903 |
| **SQ-29548** | | 144.462747 | 145.602381 | 123.189675 | 127.880103 | 91.9245892 | 126.6119 | 9.736069 |
| **$H_2O_2$** | initial | 144.462747 | 153.218694 | 123.189675 | 127.880103 | 117.106709 | 133.1716 | 6.764228 |
| | 1.00E-06 | 144.462747 | 153.218694 | 123.189675 | 127.880103 | 117.106709 | 133.1716 | 6.764228 |
| | 3.00E-06 | 144.462747 | 153.218694 | 129.469781 | 138.640008 | 124.069614 | 137.9722 | 5.199047 |
| | 1.00E-05 | 143.859606 | 150.054378 | 129.469781 | 138.640008 | 124.069614 | 137.2187 | 4.710735 |
| | 3.00E-05 | 177.605855 | 150.054378 | 126.251375 | 138.640008 | 119.005684 | 142.3115 | 10.29615 |
| | 1.00E-04 | 182.788004 | 174.300437 | 145.987151 | 134.866138 | 150.362485 | 157.6608 | 8.992747 |
| | 3.00E-04 | 182.788004 | 188.470363 | 188.265094 | 135.534453 | 173.421212 | 173.6958 | 9.923669 |
| | 1.00E-03 | 194.111279 | 190.409926 | 188.265094 | 174.148637 | 184.555062 | 186.298 | 3.409322 |
| | 3.00E-03 | 197.942854 | 190.996141 | 188.265094 | 198.763006 | 189.195003 | 193.0324 | 2.219815 |
| | 1.00E-02 | 197.942854 | 191.975449 | 188.265094 | 203.193 | 189.195003 | 194.1143 | 2.829028 |
| | initial | 185.599664 | 192.803324 | 183.606005 | 214.587924 | 177.461417 | 190.8117 | 6.428813 |
| **Norepinephrine** | 1.00E-09 | 185.599664 | 192.803324 | 183.606005 | 214.587924 | 177.461417 | 190.8117 | 6.428813 |
| | 1.00E-08 | 185.599664 | 182.99285 | 177.135973 | 211.42295 | 179.954665 | 187.4212 | 6.166633 |
| | 1.00E-07 | 185.599664 | 182.99285 | 177.135973 | 200.662006 | 179.954665 | 185.269 | 4.102579 |
| | 0.000001 | 185.599664 | 190.069783 | 177.135973 | 200.662006 | 179.954665 | 186.6844 | 4.150137 |
| | 0.00001 | 185.599664 | 190.069783 | 177.135973 | 200.662006 | 179.954665 | 186.6844 | 4.150137 |
| | without $Ca^{2+}$ | 192.25909 | 194.88692 | 188.265094 | 203.850573 | 193.07433 | 194.4672 | 2.583658 |

## Figure 5. Panel B

| Date (number of experiments) | | *20111025* | *20111102* | *20111117* | *20111116* | *20111115* | *Average* | *± SEM* |
|---|---|---|---|---|---|---|---|---|
| | **Concentration (M)** | **Diameter (μm)** | | | | | **Diameter (μm)** | |
| | before incubation | | 130.111916 | 96.216 | 137.99545 | 166.43086 | 132.6886 | 14.44412406 |
| | initial | 159.326246 | 119.474452 | 82.3119081 | 113.322907 | 151.873842 | 125.2619 | 13.9450905 |
| **5HT** | 1.00E-09 | 159.326246 | 119.474452 | 82.3119081 | 113.322907 | 151.873842 | 125.2619 | 13.9450905 |
| | 1.00E-08 | 159.326246 | 119.474452 | 78.492 | 110.158369 | 148.107125 | 123.1116 | 14.33213266 |
| | 1.00E-07 | 150.489009 | 114.599225 | 78.492 | 106.360952 | 146.118827 | 119.212 | 13.31685801 |
| | 0.000001 | 130.810646 | 117.644379 | 73.4389129 | 98.2150077 | 140.91325 | 112.2044 | 12.03837605 |
| | 0.00001 | 126.884527 | 71.12354 | 51.0537161 | 69.1825829 | 131.773509 | 90.00358 | 16.44991331 |
| | initial | 134.521064 | 105.085628 | 73.428 | 102.54795 | 121.917845 | 107.5001 | 10.31810387 |
| **Acethylcholine** | 1.00E-09 | 134.521064 | 105.085628 | 73.428 | 102.54795 | 121.917845 | 107.5001 | 10.31810387 |
| | 1.00E-08 | 134.521064 | 105.085628 | 73.428 | 102.54795 | 138.736794 | 110.8639 | 11.91752851 |
| | 1.00E-07 | 147.335428 | 131.665526 | 84.9730275 | 136.75144 | 162.784407 | 132.702 | 13.06639866 |
| | 0.000001 | 149.131629 | 133.569001 | 93.7032432 | 144.957 | 159.980027 | 136.2682 | 11.45049127 |
| | 0.00001 | 156.339467 | 133.569001 | 93.7032432 | 155.723146 | 166.574045 | 141.1818 | 13.03483752 |
| **SQ-29548** | | 134.521064 | 89.273199 | 74.1285981 | 110.820203 | 134.977514 | 108.7441 | 12.11260313 |
| **$H_2O_2$** | initial | 134.521064 | 85.6446911 | 74.1285981 | 109.65709 | 137.570865 | 108.3045 | 12.7025924 |
| | 0.000001 | 134.521064 | 85.6446911 | 74.1285981 | 109.65709 | 137.570865 | 108.3045 | 12.7025924 |
| | 0.000003 | 136.645919 | 85.6446911 | 75.2178771 | 109.65709 | 137.570865 | 108.9473 | 12.78223631 |
| | 0.00001 | 138.024485 | 81.679079 | 75.2178771 | 114.714551 | 135.674802 | 109.0622 | 13.18003629 |
| | 0.00003 | 140.169127 | 81.679079 | 75.2178771 | 114.714551 | 149.689441 | 112.294 | 14.98868417 |
| | 0.0001 | 142.318055 | 72.1731028 | 78.3617159 | 127.390364 | 163.511388 | 116.7509 | 17.90855985 |
| | 0.0003 | 142.318055 | 72.1731028 | 78.4358213 | 135.581738 | 164.809918 | 118.6637 | 18.37781248 |
| | 0.001 | 158.591447 | 135.674802 | 78.4358213 | 143.093005 | 166.088629 | 136.3767 | 15.45807317 |
| | 0.003 | 158.591447 | 138.697801 | 88.0939517 | 149.473809 | 165.795252 | 140.1305 | 13.77795065 |
| | 0.01 | 158.591447 | 138.697801 | 93.2252333 | 155.822179 | 165.795252 | 142.4264 | 13.07959508 |
| | initial | 160.002562 | 138.697801 | 98.1660388 | 143.171396 | | 135.0094 | 13.10951905 |
| **5HT** | 1.00E-09 | 160.002562 | 138.697801 | 98.1660388 | 145.068908 | | 135.4838 | 13.21611731 |
| | 1.00E-08 | 164.124107 | 138.697801 | 98.1660388 | 145.068908 | | 136.5142 | 13.87695806 |
| | 1.00E-07 | 157.066929 | 138.697801 | 96.224324 | 141.906402 | | 133.4739 | 13.0465774 |
| | 0.000001 | 157.066929 | 138.697801 | 94.9584442 | 141.906402 | | 133.1574 | 13.34811752 |
| | 0.00001 | 157.066929 | 138.697801 | 94.9584442 | 141.906402 | | 133.1574 | 13.34811752 |
| | without $Ca^{2+}$ | 159.769504 | 138.697801 | 95.5578509 | 157.618272 | 166.43086 | 143.6149 | 12.86718994 |

## Figure 5. Panel C

| Date (number of experiments) | | *20111006* | *20111010* | *20111013* | *20111012* | *20111019* | *Average* | *± SEM* |
|---|---|---|---|---|---|---|---|---|
| | **Concentration (M)** | **Diameter (µm)** | | | | | **Diameter (µm)** | |
| | before incubation | 171.641077 | 106.4588515 | 200.9961988 | 133.33631 | 117.3374376 | 145.954 | 17.64575 |
| | initial | 134.53149 | 91.66267908 | 171.8277234 | 101.2997813 | 109.510823 | 121.7665 | 14.39514 |
| **5HT** | 1.00E-09 | 134.53149 | 84.45273312 | 171.8277234 | 101.2997813 | 109.510823 | 120.3245 | 15.19882 |
| | 1.00E-08 | 132.5934782 | 79.37779488 | 171.8277234 | 96.95651505 | 109.510823 | 118.0533 | 15.99523 |
| | 1.00E-07 | 125.4937502 | 73.39798314 | 160.4502004 | 89.39429826 | 83.56798902 | 106.4608 | 16.10218 |
| | 0.000001 | 83.21481933 | 60.42747132 | 111.5607556 | 68.79635166 | 76.63222701 | 80.12632 | 8.7358 |
| | 0.00001 | 74.27979012 | 60.42747132 | 107.3191745 | 63.44226675 | 66.78974799 | 74.45169 | 8.534906 |
| | initial | 108.7157877 | 69.87414177 | 140.3491082 | 99.04376925 | 92.85917568 | 102.1684 | 11.48908 |
| **Acethylcholine** | 1.00E-09 | 108.7157877 | 69.87414177 | 140.3491082 | 98.43302553 | 92.85917568 | 102.0462 | 11.49803 |
| | 1.00E-08 | 108.7157877 | 87.47546637 | 140.3491082 | 98.43302553 | 92.85917568 | 105.5665 | 9.379221 |
| | 1.00E-07 | 147.5148074 | 95.91777471 | 140.3491082 | 113.518979 | 92.85917568 | 118.032 | 11.2035 |
| | 0.000001 | 167.5908645 | 95.91777471 | 146.3448968 | 131.3135585 | 96.08681103 | 127.4508 | 14.07332 |
| | 0.00001 | 168.6110453 | 94.91201367 | 168.4886104 | 135.9182348 | 95.98459419 | 132.7829 | 16.36567 |
| **U46619** | initial | 138.1391026 | 87.34941075 | 155.8016066 | 116.0998656 | 99.28418898 | 119.3348 | 12.48797 |
| | 1.00E-10 | 138.1391026 | 87.34941075 | 155.8016066 | 116.0998656 | 99.28418898 | 119.3348 | 12.48797 |
| | 1.00E-09 | 138.1391026 | 87.08064528 | 155.8016066 | 115.702291 | 100.0580631 | 119.3563 | 12.46705 |
| | 1.00E-08 | 123.4934196 | 79.81324191 | 151.4656706 | 115.702291 | 94.47830106 | 112.9906 | 12.33174 |
| | 1.00E-07 | 79.79818284 | 66.65763456 | 130.0487676 | 109.6972795 | 83.21964279 | 93.8843 | 11.42664 |
| | 0.000001 | 50.70326202 | 63.85774896 | 91.81118721 | 79.67255133 | 66.8107446 | 70.5711 | 7.028222 |
| | 0.00001 | 48.80672439 | 59.65667988 | 89.96620743 | 71.35232337 | 66.8107446 | 67.31854 | 6.822161 |
| | without $Ca^{2+}$ | 151.8474519 | 106.4588515 | 175.2804409 | 137.640748 | 111.3648357 | 136.5185 | 12.79651 |

## Figure 5. Panel C

| Date (number of experiments) | | *20111004* | *20111005* | *20101207* | *20101208* | *20101209* | *Average* | *± SEM* |
|---|---|---|---|---|---|---|---|---|
| | **Concentration (M)** | **Diameter (µm)** | | | | | **Diameter (µm)** | |
| | before incubation | 215.2796 | 196.37697 | 259.37811 | 262.00763 | 251.03188 | 236.8148 | 13.1242 |
| | initial | 149.6145 | 177.402694 | 230.0087 | 269.02972 | 239 | 213.0111 | 21.66739 |
| **Norepinephrine** | 1.00E-09 | 149.6145 | 177.402694 | 230.0087 | 269.02972 | 238 | 212.8111 | 21.60826 |
| | 1.00E-08 | 144.4627 | 169.181612 | 220.00227 | 269.02972 | 238 | 208.1353 | 22.70732 |
| | 1.00E-07 | 138.1304 | 169.181612 | 210.00952 | 269.02972 | 224.00223 | 202.0707 | 22.5805 |
| | 0.000001 | 104.0318 | 163.854101 | 175 | 257.03113 | 205.00244 | 180.9839 | 25.11069 |
| | 0.00001 | 61.51835 | 67.7783104 | 170.01176 | 127.67537 | 70 | 99.39676 | 21.31294 |
| | initial | 146.5733 | 163.069687 | 218.02065 | 215.00232 | 230.00217 | 194.5336 | 16.61144 |
| **Acethylcholine** | 1.00E-09 | 146.5733 | 163.069687 | 218.02065 | 215.00232 | 230.00217 | 194.5336 | 16.61144 |
| | 1.00E-08 | 180.4949 | 163.069687 | 218.02065 | 215.00232 | 230.00217 | 201.3179 | 12.62053 |
| | 1.00E-07 | 196.2392 | 163.069687 | 218.02065 | 240.00209 | 232.00215 | 209.8668 | 13.8569 |
| | 0.000001 | 202.5956 | 163.069687 | 243.01852 | 262.00192 | 237.01898 | 221.5409 | 17.4868 |
| | 0.00001 | 202.6796 | 189.904209 | 249 | 265.00189 | 237.01898 | 228.7209 | 14.10903 |
| **U46619** | initial | 190.533 | 165.311191 | 187.00267 | 200.02249 | 203.00246 | 189.1744 | 6.651734 |
| | 1.00E-10 | 190.533 | 165.311191 | 187.00267 | 200.02249 | 203.00246 | 189.1744 | 6.651734 |
| | 1.00E-09 | 190.533 | 165.311191 | 182.01099 | 194.04123 | 197.00253 | 185.7798 | 5.700709 |
| | 1.00E-08 | 190.533 | 158.985103 | 175.02571 | 173.07224 | 190.00262 | 177.5237 | 5.893642 |
| | 1.00E-07 | 140.5773 | 119.744129 | 143.03146 | 149.27156 | 160.00313 | 142.5255 | 6.61067 |
| | 0.000001 | 72.81976 | 60.1483247 | 114.00439 | 70.34202 | 95.7549 | 82.61388 | 9.770555 |
| | 0.00001 | 44.31 | 58.236 | 66.0303 | 57.56735 | 65.96969 | 58.42267 | 3.967162 |
| | without $Ca^{2+}$ | 203.8506 | 193.07433 | 257.01752 | 224.0558 | 223.02017 | 220.2037 | 10.91205 |

## Figure 6. Panel A,B

| Date (number of experiments) | | 20121002 | 20121017 | 20121016 | 20121107 | 20121106 | Average | ± SEM |
|---|---|---|---|---|---|---|---|---|
| | Concentration (M) | Diameter (μm) | | | | | Diameter (μm) | |
| Norepinephrine | 1.00E-09 | 352.103351 | 372.3595 | 316.810012 | 316.68288 | | 339.4889 | 13.76604 |
| | 1.00E-08 | 347.110905 | 372.3595 | 321.555271 | 314.152368 | | 338.7945 | 13.22938 |
| | 1.00E-07 | 273.74304 | 364.9782 | 308.15204 | 286.84959 | | 308.4307 | 20.13842 |
| | 0.000001 | 174.939482 | 364.9782 | 156.985272 | 185.683834 | | 220.6467 | 48.47328 |
| | 0.00001 | 160.364024 | 190.5961 | 101.944447 | 181.784548 | | 158.6723 | 19.94621 |
| Acethylcholine | 1.00E-09 | 235.946034 | 315.6697 | 250.323294 | 268.633743 | 217.5992 | 257.6344 | 16.75669 |
| | 1.00E-08 | 240.297504 | 320.0859 | 258.502825 | 268.633743 | 217.5992 | 261.0238 | 17.12873 |
| | 1.00E-07 | 347.698544 | 336.1796 | 326.658061 | 324.513609 | 279.0331 | 322.8166 | 11.68827 |
| | 0.000001 | 360.230938 | 355.8794 | 324.21094 | 343.480505 | 313.5823 | 339.4768 | 8.998278 |
| | 0.00001 | 359.965546 | 349.8864 | 320.995287 | 342.979137 | 374.8579 | 349.7369 | 8.965098 |
| $H_2O_2$ | 1.00E-06 | 250.787055 | 354.4941 | 241.691642 | 305.529888 | 216.4943 | 273.7994 | 24.86243 |
| | 3.00E-06 | 250.787055 | 354.4941 | 241.691642 | 305.529888 | 216.4943 | 273.7994 | 24.86243 |
| | 1.00E-05 | 250.787055 | 348.8111 | 222.791718 | 302.508434 | 209.5842 | 266.8965 | 25.94963 |
| | 3.00E-05 | 233.75619 | 339.2886 | 208.049104 | 292.358994 | 210.217 | 256.734 | 25.63828 |
| | 1.00E-04 | 210.042528 | 312.0748 | 192.981976 | 273.515344 | 180.8941 | 233.9017 | 25.23268 |
| | 3.00E-04 | 197.321444 | 287.6336 | 173.319508 | 256.270423 | 183.4193 | 219.5929 | 22.27167 |
| | 1.00E-03 | 229.2439 | 295.5432 | 218.003959 | 310.394702 | 204.459 | 251.529 | 21.49251 |
| | 3.00E-03 | 390.368093 | 355.2399 | 313.340121 | 351.342934 | 311.9052 | 344.4393 | 14.66184 |

## Figure 6. Panel A, B

| Date (number of experiments) | | 20121017 | 20121016 | 20121002 | 20121106 | 20121107 | Average | ± SEM |
|---|---|---|---|---|---|---|---|---|
| | Concentration (M) | | | $F_{340/380}$ | | | $F_{340/380}$ | |
| Norepinephrine | 1.00E-09 | 0.8556701 | 0.90243902 | 0.90322581 | 1.05 | 1.06593407 | 0.955454 | 0.042802 |
| | 1.00E-08 | 0.85416667 | 0.91358025 | 0.91489362 | 1.16216216 | 1.06593407 | 0.982147 | 0.057022 |
| | 1.00E-07 | 0.88421053 | 1.06493506 | 0.96261682 | 1.20547945 | 1.07692308 | 1.038833 | 0.054599 |
| | 0.000001 | 0.9139785 | 1.40495868 | 1.14049587 | 1.45454545 | 1.11702128 | 1.2062 | 0.099711 |
| | 0.00001 | 1.10619469 | 1.53448276 | 1.32407407 | 1.44776119 | 1.36666667 | 1.355836 | 0.072046 |
| Acethylcholine | 1.00E-09 | 1.03409091 | 1.03488372 | 0.96212121 | 1.27160494 | 0.94117647 | 1.048775 | 0.0588 |
| | 1.00E-08 | 1.02272727 | 0.82291667 | 0.96923077 | 1.275 | 0.93984962 | 1.005945 | 0.074796 |
| | 1.00E-07 | 1.02298851 | 0.80851064 | 0.94117647 | 1.11235955 | 0.93939394 | 0.964886 | 0.050393 |
| | 0.000001 | 1.02439024 | 0.80851064 | 0.85981308 | 0.9893617 | 0.93577982 | 0.923571 | 0.039966 |
| | 0.00001 | 1.01204819 | 0.79787234 | 0.85849057 | 0.9893617 | 0.92938976 | 0.917433 | 0.040024 |
| $H_2O_2$ | 1.00E-06 | 1.03846154 | 1.1625 | 1.16666667 | 1.2173913 | 1.0625 | 1.129504 | 0.033891 |
| | 3.00E-06 | 1.03846154 | 1.17721519 | 1.19277108 | 1.23529412 | 1.08510638 | 1.14577 | 0.036337 |
| | 1.00E-05 | 1.05128205 | 1.17857143 | 1.21428571 | 1.23880597 | 1.08080808 | 1.152751 | 0.036966 |
| | 3.00E-05 | 1.0625 | 1.16363636 | 1.22352941 | 1.20289855 | 1.05769231 | 1.142051 | 0.034822 |
| | 1.00E-04 | 1.03529412 | 1.16326531 | 1.21590909 | 1.17391304 | 1.05660377 | 1.128997 | 0.03519 |
| | 3.00E-04 | 1.02298851 | 1.14285714 | 1.20430108 | 1.1 | 1.04545455 | 1.10312 | 0.032843 |
| | 1.00E-03 | 1.01162791 | 0.98734177 | 1.01149425 | 0.98780488 | 1.02884615 | 1.005423 | 0.007941 |

## Figure 6. Panel C

| Date (number of experiments) | | *20140415* | *20140415* | *20140416* | *20140422* | *20140422* | *Average* | *± SEM* |
|---|---|---|---|---|---|---|---|---|
| | **Concentration (M)** | **Diameter (μm)** | | | | | **Diameter (μm)** | |
| | initial | 302.234801 | 247.648653 | 280.533282 | 286.927804 | 274.488524 | 278.3666 | 8.961896 |
| **U46619** | 1.00E-09 | 302.234801 | 247.648653 | 280.533282 | 286.927804 | 274.488524 | 278.3666 | 8.961896 |
| | 1.00E-08 | 275.256037 | 243.231851 | 280.533282 | 286.927804 | 270.559924 | 271.3018 | 7.528282 |
| | 1.00E-07 | 207.632691 | 219.635485 | 268.383037 | 286.927804 | 243.303501 | 245.1765 | 14.74297 |
| | 0.000001 | 141.568583 | 143.446795 | 196.52382 | 228.754857 | 208.86794 | 183.8324 | 17.63959 |
| | 0.00001 | 135.940345 | 127.687253 | 183.045389 | 146.015965 | 191.690339 | 156.8759 | 12.85515 |

## Figure 6. Panel C

| Date (number of experiments) | | 20140415 | 20140415d | 20140416 | 20140422 | 20140422d | Average | ± SEM |
|---|---|---|---|---|---|---|---|---|
| | Concentration (M) | | | $F_{340/380}$ | | | $F_{340/380}$ | |
| **U46619** | 0.000000001 | 0.92105263 | 0.872881 | 0.6875 | 0.90804598 | 0.97 | 0.871896 | 0.048657 |
| | 0.00000001 | 0.92207792 | 0.867769 | 0.67708333 | 0.90909091 | 0.979592 | 0.871123 | 0.051704 |
| | 0.0000001 | 0.96103896 | 0.866667 | 0.6875 | 0.92134831 | 0.979592 | 0.883229 | 0.05262 |
| | 0.000001 | 0.98863636 | 0.896 | 0.73958333 | 0.93859649 | 0.989691 | 0.910501 | 0.046146 |
| | 0.00001 | 1 | 0.917293 | 0.8 | 0.94890511 | 0.989247 | 0.931089 | 0.035933 |

## Figure 6. Panel D

| Date (number of experiments) | | *20130227* | *20130226* | *20130306* | *20130321* | *20130321* | *Average* | *± SEM* |
|---|---|---|---|---|---|---|---|---|
| | **Concentration (M)** | **Diameter (µm)** | | | | | **Diameter (µm)** | |
| **Norepinephrine** | 1.00E-09 | 296.0342871 | 307.2926605 | 267.8809348 | 340.8386285 | 280.2875069 | 298.4668 | 12.54027 |
| | 1.00E-08 | 291.0127487 | 307.2926605 | 243.115677 | 340.8386285 | 270.3510401 | 290.5222 | 16.53403 |
| | 1.00E-07 | 279.1537153 | 281.7561112 | 229.8731524 | 303.3021399 | 259.5925088 | 270.7355 | 12.34262 |
| | 0.000001 | 200.8296629 | 178.5239645 | 178.2544268 | 213.7347668 | 168.7963054 | 188.0278 | 8.307912 |
| | 0.00001 | 156.4854872 | 111.3216525 | 141.1263562 | 200.1281406 | 159.3815571 | 153.6886 | 14.40422 |
| **Acethylcholine** | 1.00E-09 | 265.559901 | 283.1102755 | 209.302007 | 262.5088537 | 249.1391847 | 253.924 | 12.39949 |
| | 1.00E-08 | 265.559901 | 283.1102755 | 209.302007 | 262.5088537 | 249.1391847 | 253.924 | 12.39949 |
| | 1.00E-07 | 265.559901 | 283.1102755 | 218.2537728 | 261.9136438 | 249.1391847 | 255.5954 | 10.79898 |
| | 0.000001 | 286.3182941 | 295.6930052 | 259.1104982 | 320.4462043 | 287.4551938 | 289.8046 | 9.832047 |
| | 0.00001 | 313.4270699 | 304.5526061 | 259.1104982 | 343.5347464 | 305.1243887 | 305.1499 | 13.53125 |
| **$H_2O_2$** | 1.00E-06 | | 285.4008809 | 206.8602982 | 334.4427142 | 284.5600797 | 277.816 | 26.36966 |
| | 3.00E-06 | | 285.4008809 | 203.6411324 | 337.9893942 | 284.5600797 | 277.8979 | 27.72745 |
| | 1.00E-05 | | 280.3468317 | 201.0320709 | 333.1529134 | 297.5773385 | 278.0273 | 27.92055 |
| | 3.00E-05 | | 293.6935924 | 218.5134041 | 335.9769226 | 284.6227277 | 283.2017 | 24.29317 |
| | 1.00E-04 | | 293.6935924 | 232.649674 | 329.7808654 | 282.7477184 | 284.718 | 20.05454 |
| | 3.00E-04 | | 299.7427113 | 232.649674 | 329.7808654 | 304.2050828 | 291.5946 | 20.73265 |
| | 1.00E-03 | | 299.7427113 | 246.4354518 | 340.7804051 | 304.2050828 | 297.7909 | 19.43028 |

## Figure 6. Panel D

| Date (number of experiments) | | *20130221* | *20130221* | *20130227* | *20130226* | *20130306* | *Average* | *± SEM* |
|---|---|---|---|---|---|---|---|---|
| | **Concentration (M)** | $F_{340/380}$ | | | | | $F_{340/380}$ | |
| | | 0.99065421 | 1.01869159 | 0.88571429 | 0.95 | 0.89411765 | 0.947836 | 0.02608 |
| **Norepinephrine** | 1.00E-09 | 0.99056604 | 1.01869159 | 0.90196078 | 0.96402878 | 0.91666667 | 0.958383 | 0.021941 |
| | 1.00E-08 | 0.99074074 | 1.01869159 | 0.94392523 | 0.97142857 | 0.94047619 | 0.973052 | 0.014677 |
| | 1.00E-07 | 1.04504505 | 1.09482759 | 1.06338028 | 1.00649351 | 0.97752809 | 1.037455 | 0.020702 |
| | 0.000001 | 1.0610687 | 1.14655172 | 1.1942446 | 1.10691824 | 1.09183673 | 1.120124 | 0.023081 |
| **Acethylcholine** | 0.00001 | 1.03921569 | 1.04587156 | 1.03092784 | 0.97777778 | 0.95959596 | 1.010678 | 0.017543 |
| | 1.00E-09 | 1.02912621 | 1.0462963 | 1.04123711 | 0.97744361 | 0.95 | 1.008821 | 0.019121 |
| | 1.00E-08 | 1.03 | 1.04716981 | 1.04494382 | 0.97744361 | 0.9375 | 1.007411 | 0.021547 |
| | 1.00E-07 | 1.02040816 | 1.03809524 | 1.02352941 | 0.96153846 | 0.92783505 | 0.994281 | 0.021148 |
| | 0.000001 | 0.91964286 | 1.01886792 | 1.01176471 | 0.93333333 | 0.92708333 | 0.962138 | 0.021847 |
| **$H_2O_2$** | 0.00001 | 1.1047619 | 1.10655738 | 0.96296296 | 0.9921875 | 0.96907216 | 1.027108 | 0.032438 |
| | 1.00E-06 | 1.10576923 | 1.10743802 | 0.96296296 | 1.00787402 | 0.96938776 | 1.030686 | 0.031932 |
| | 3.00E-06 | 1.1047619 | 1.11570248 | 0.96296296 | 1.00787402 | 0.96969697 | 1.0322 | 0.03281 |
| | 1.00E-05 | 1.1047619 | 1.125 | 0.96296296 | 1.00787402 | 0.97916667 | 1.035953 | 0.03317 |
| | 3.00E-05 | 1.125 | 1.125 | 0.975 | 1.00793651 | 0.96875 | 1.040337 | 0.035199 |
| | 1.00E-04 | 1.11538462 | 1.13333333 | 0.9875 | 1.01587302 | 0.9893617 | 1.048291 | 0.031586 |
| | 3.00E-04 | 1.13592233 | 1.125 | 1.06493506 | 1.032 | 1.02150538 | 1.075873 | 0.023473 |
| | 1.00E-03 | 1.14545455 | 1.15625 | 1.13333333 | 1.06451613 | 1.06593407 | 1.113098 | 0.019879 |

## Supporting Fig. 1.

| Date (number of experiments) | | 20120425 | 20120515 | 20120517 | Average | ± SEM |
|---|---|---|---|---|---|---|
| | Concentration (M) | Diameter (μm) | | | Diameter (μm) | |
| | before incubation | 208.985893 | 225.370237 | 210.1684 | 214.8415 | 5.275419 |
| | initial | 181.042425 | 193.10236 | 174.300437 | 182.8151 | 5.499539 |
| **Norepinephrine** | 1.00E-09 | 181.042425 | 193.10236 | 174.300437 | 182.8151 | 5.499539 |
| | 1.00E-08 | 176.744215 | 188.027637 | 168.578959 | 177.7836 | 5.638351 |
| | 1.00E-07 | 176.744215 | 183.574368 | 166.682263 | 175.6669 | 4.905989 |
| | 0.000001 | 169.181612 | 172.205093 | 126.606324 | 155.9977 | 14.72157 |
| | 0.00001 | 62.063061 | 93.1886965 | 62.0372283 | 72.42966 | 10.37952 |
| | initial | 156.284351 | 163.436631 | 173.515909 | 164.4123 | 4.998186 |
| **Acethylcholine** | 1.00E-09 | 156.284351 | 169.484497 | 173.515909 | 166.4283 | 5.203752 |
| | 1.00E-08 | 156.284351 | 169.484497 | 173.515909 | 166.4283 | 5.203752 |
| | 1.00E-07 | 165.066207 | 180.523782 | 179.193786 | 174.9279 | 4.945784 |
| | 0.000001 | 190.457274 | 224.843347 | 203.669662 | 206.3234 | 10.0147 |
| | 0.00001 | 199.249245 | 234.912098 | 207.340106 | 213.8338 | 10.79484 |
| **NS-398** | | 156.720887 | 143.059399 | 169.701856 | 156.494 | 7.691851 |
| **$H_2O_2$** | initial | 151.133308 | 143.059399 | 169.701856 | 154.6315 | 7.8874 |
| | 1,00E-06 | 151.133308 | 143.059399 | 169.701856 | 154.6315 | 7.8874 |
| | 3,00E-06 | 143.859606 | 143.059399 | 169.701856 | 152.207 | 8.750501 |
| | 1,00E-05 | 140.698398 | 138.62845 | 167.170723 | 148.8325 | 9.18855 |
| | 3,00E-05 | 136.975408 | 134.842369 | 164.122866 | 145.3135 | 9.424796 |
| | 1,00E-04 | 136.975408 | 129.771172 | 160.961335 | 142.5693 | 9.428244 |
| | 3,00E-04 | 146.454368 | 124.707425 | 154.60887 | 141.9236 | 8.92413 |
| | 1,00E-03 | 184.44756 | 134.924064 | 219.856136 | 179.7426 | 24.63038 |
| | 3,00E-03 | 187.608426 | 234.213418 | 219.651 | 213.8243 | 13.76553 |
| | 1,00E-02 | 190.738985 | 234.213418 | 219.651 | 214.8678 | 12.77583 |
| | initial | 187.369063 | 217.752 | 218.393248 | 207.8381 | 10.23619 |
| **Norepinephrine** | 1.00E-09 | 187.369063 | 208.257 | 218.393248 | 204.6731 | 9.133423 |
| | 1.00E-08 | 184.837082 | 213.32195 | 218.393248 | 205.5174 | 10.44329 |
| | 1.00E-07 | 184.837082 | 206.358968 | 214.590728 | 201.9289 | 8.870153 |
| | 0.000001 | 184.837082 | 206.991 | 214.590728 | 202.1396 | 8.925094 |
| | 0.00001 | 184.837082 | 206.991 | 208.893842 | 200.2406 | 7.721343 |
| | without $Ca^{2+}$ | 193.090947 | 238.641829 | 221.623232 | 217.7853 | 13.28869 |

## Supporting Fig. 2. Panel A

| Date (number of experiments) | | *20140505* | *20140505d* | *20140506* | *20140506d* | *20140507* | *20140507d* | *Average* | *± SEM* |
|---|---|---|---|---|---|---|---|---|---|
| | **Concentration (M)** | **Diameter (μm)** | | | | | | **Diameter (μm)** | |
| | initial | 196.4911948 | 166.556 | 170.9287558 | 125.3915 | 176.1663054 | 189.9 | 170.9056 | 10.22014 |
| **Norepinephrine** | 1.00E-09 | 196.4911948 | 166.556 | 170.9287558 | 125.3915 | 176.1663054 | 189.9 | 170.9056 | 10.22014 |
| | 1.00E-08 | 196.4911948 | 162.1086 | 165.2323951 | 122.228 | 171.7402491 | 186.735 | 167.4226 | 10.50743 |
| | 1.00E-07 | 196.4911948 | 148.2572 | 165.2323951 | 117.1888 | 146.890106 | 179.772 | 158.9719 | 11.38147 |
| | 0.000001 | 190.1698859 | 117.8469 | 150.1291291 | 63.93613 | 67.30073091 | 143.7412 | 122.1873 | 20.24148 |
| | 0.00001 | 49.41050511 | 44.35067 | 52.55424897 | 31.04283 | 39.64217571 | 48.74511 | 44.29092 | 3.222106 |
| **U73122** | | | | | | | | | |
| | initial | 187.3947379 | 142.425 | 152.5858274 | 144.9584 | 164.6104283 | 133.6005 | 154.2625 | 7.876648 |
| **Norepinephrine** | 1.00E-09 | 187.3947379 | 142.425 | 152.5858274 | 144.9584 | 164.6104283 | 133.6005 | 154.2625 | 7.876648 |
| | 1.00E-08 | 187.3947379 | 142.425 | 148.1882624 | 144.9584 | 161.4460233 | 133.6005 | 153.0021 | 7.810944 |
| | 1.00E-07 | 187.3947379 | 143.058 | 145.0247057 | 128.5006 | 161.4460233 | 127.2724 | 148.7827 | 9.259597 |
| | 0.000001 | 187.3947379 | 125.9734 | 67.65997107 | 56.80448 | 140.0990732 | 90.52786 | 111.4099 | 20.11693 |
| | 0.00001 | 184.230181 | 96.26804 | 73.74381636 | 67.40187 | 102.4150196 | 82.94474 | 101.1673 | 17.4635 |

## Supporting Fig. 2. Panel B

| Date (number of experiments) | | *20140508* | *20140508* | *20140513* | *20140513* | *Average* | *± SEM* |
|---|---|---|---|---|---|---|---|
| | **Concentration (M)** | **Diameter (µm)** | | | | **Diameter (µm)** | |
| | initial | 113.3140706 | 124.8310625 | 182.9643709 | 148.1612144 | 142.3177 | 15.36579406 |
| **U46619** | 1.00E-09 | 113.3140706 | 124.8310625 | 182.9643709 | 148.1612144 | 142.3177 | 15.36579406 |
| | 1.00E-08 | 108.2596542 | 124.8310625 | 182.9643709 | 148.1612144 | 141.0541 | 16.19064674 |
| | 1.00E-07 | 104.4622619 | 124.8310625 | 182.9643709 | 141.0113148 | 138.3173 | 16.65498139 |
| | 0.000001 | 73.47164535 | 70.33425048 | 102.5635784 | 73.07792568 | 79.86185 | 7.599341913 |
| | 0.00001 | 29.751 | 29.77792149 | 43.71826527 | 50.04304302 | 38.32256 | 5.106905828 |
| | initial | 127.2581871 | 121.0685928 | 127.9662419 | 136.7177454 | 128.2527 | 3.218947863 |
| **Acethylcholine** | 1.00E-09 | 127.2581871 | 121.0685928 | 133.0264249 | 135.1199205 | 129.1183 | 3.156351989 |
| | 1.00E-08 | 127.2581871 | 127.3903638 | 133.0264249 | 135.1199205 | 130.6987 | 1.99473706 |
| | 1.00E-07 | 127.2581871 | 136.2729996 | 133.0264249 | 169.9578541 | 141.6289 | 9.625206818 |
| | 0.000001 | 146.8682739 | 141.5685827 | 147.6532698 | 179.3178415 | 153.852 | 8.595483848 |
| | 0.00001 | 155.8003217 | 148.7765473 | 183.7270916 | 188.9566021 | 169.3151 | 9.991560971 |
| **U73122 10 µM** | | 123.4414883 | 132.4483566 | 168.0135123 | 140.1376798 | 141.0103 | 9.625941337 |
| | initial | 117.105 | 132.4196121 | 178.5968925 | 140.1376798 | 142.0648 | 13.08411517 |
| **U46619** | 1.00E-09 | 117.105 | 132.4196121 | 178.5968925 | 140.1376798 | 142.0648 | 13.08411517 |
| | 1.00E-08 | 110.775 | 131.7613491 | 169.739621 | 131.9087558 | 136.0462 | 12.2792459 |
| | 1.00E-07 | 105.078 | 130.0256631 | 149.5220567 | 117.8978452 | 125.6309 | 9.453055716 |
| | 0.000001 | 53.86453998 | 75.98373117 | 87.05303382 | 56.90314254 | 68.45111 | 7.899870932 |
| | 0.00001 | 27.22636179 | 39.879 | 40.1244141 | 40.27889775 | 36.87717 | 3.21798887 |
| | Without Ca2+ | 155.719285 | 158.9447808 | 200.0440339 | 200.1661776 | 178.7186 | 12.36508906 |
